# Shifts in the immunoepigenomic landscape of monocytes in response to a diabetes-specific social support intervention: a pilot study among Native Hawaiian adults with diabetes

**DOI:** 10.1186/s13148-022-01307-6

**Published:** 2022-07-18

**Authors:** Christian K. Dye, Michael J. Corley, Claire Ing, Annette Lum-Jones, Dongmei Li, Marjorie K. L. M. Mau, Alika K. Maunakea

**Affiliations:** 1 Department of Molecular Biosciences and Bioengineering, University of Hawaii, Honolulu, HI 96822 USA; 2 Cornell Center for Immunology, Weill Cornell Medical Center, Cornell University, New York, NY 10065 USA; 3 Department of Native Hawaiian Health, John A. Burns School of Medicine, University of Hawaii, Honolulu, HI 96813 USA; 4 University of Hawaii Cancer Center, University of Hawaii, Honolulu, HI 96813 USA; 5 Department of Clinical and Translational Research, School of Medicine and Dentistry, University of Rochester, Rochester, NY 14642 USA; 6 Department of Anatomy, Biochemistry and Physiology, John A. Burns School of Medicine, University of Hawaii, 651 Ilalo St. BSB222-K, Honolulu, HI 96813 USA

**Keywords:** Diabetes, DNA methylation, Epigenetics, Native Hawaiians, Intervention, Monocyte, Inflammation, Immune response

## Abstract

**Background:**

Native Hawaiians are disproportionately affected by type 2 diabetes mellitus (DM), a chronic metabolic, non-communicable disease characterized by hyperglycemia and systemic inflammation. Unrelenting systemic inflammation  frequently leads to a cascade of multiple comorbidities associated with DM, including cardiovascular disease, microvascular complications, and renal dysfunction. Yet few studies have examined the link between chronic inflammation at a cellular level and its relationship to standard DM therapies such as diabetes-specific lifestyle and social support education, well recognized as the cornerstone of clinical standards of diabetes care. This pilot study was initiated to explore the association of monocyte inflammation using epigenetic, immunologic, and clinical measures following a 3-month diabetes-specific social support program among high-risk Native Hawaiian adults with DM.

**Results:**

From a sample of 16 Native Hawaiian adults with DM, monocytes enriched from peripheral blood mononuclear cells (PBMCs) of 8 individuals were randomly selected for epigenomic analysis. Using the Illumina HumanMethylation450 BeadChip microarray, 1,061 differentially methylated loci (DML) were identified in monocytes of participants at baseline and 3 months following a DM-specific social support program (DM-SSP). Gene ontology analysis showed that these DML were enriched within genes involved in immune, metabolic, and cardiometabolic pathways, a subset of which were also significantly differentially expressed. Ex vivo analysis of immune function showed improvement post-DM-SSP compared with baseline, characterized by attenuated interleukin 1β and IL-6 secretion from monocytes. Altered cytokine secretion in response to the DM-SSP was significantly associated with changes in the methylation and gene expression states of immune-related genes in monocytes between intervention time points.

**Conclusions:**

Our pilot study provides preliminary evidence of changes to inflammatory monocyte activity, potentially driven by epigenetic modifications, 3 months following a DM-specific SSP intervention. These novel alterations in the trajectory of monocyte inflammatory states were identified at loci that regulate transcription of immune and metabolic genes in high-risk Native Hawaiians with DM, suggesting a relationship between improvements in psychosocial behaviors and shifts in the immunoepigenetic patterns following a diabetes-specific SSP. Further research is warranted to investigate how social support influences systemic inflammation via immunoepigenetic modifications in chronic inflammatory diseases such as DM.

**Supplementary Information:**

The online version contains supplementary material available at 10.1186/s13148-022-01307-6.

## Background

The prevalence of diabetes has reached epidemic proportions, affecting more than 34 million people in the USA (10.5% of the population), of which 90–95% are inflicted with type 2 diabetes mellitus (DM), according to a 2020 report by the Centers for Disease Control and Prevention (CDC). Native Hawaiians and Pacific Islanders (NH/PIs) disproportionately experience a higher prevalence and earlier onset of cardiometabolic outcomes, including obesity, DM, and cardiovascular diseases (CVD), compared with Whites and the general US population [1–4]. A study of Native Hawaiian (NH) adults found the prevalence of DM to be 22.4% with an additional 15% at increased risk for DM based on impaired glucose tolerance [5]. Moreover, a 2015 CDC report for the USA has confirmed the age-adjusted prevalence of diagnosed diabetes is significantly higher for NH/PIs (19.8%) than among Whites (8.0%) and has indicated NH/PIs were younger, had lower education levels, and higher body mass indexes (BMIs) [6]. Earlier onset (on average by 10 years compared to other ethnic groups) results in a longer exposure of abnormal glucose homeostasis that contributes to the development of DM-related complications, such as macrovascular disease [7]. Compounded by unhealthy lifestyle behaviors (*e.g.*, poor diet, smoking, limited exercise, etc.), such prolonged exposure may underlie glucose intolerance, insulin resistance, and DM by promoting a chronic pro-inflammatory milieu [8].

Inflammation, primarily driven by innate immune cells, is a fundamental cellular process involved in host defense. Among these cells, monocytes are key determinants of inflammation [9]. Monocytes play a central role in acute inflammation, forming one of the first lines of defense against pathogens, foreign bodies, and injury through phagocytosis, antigen presentation, and cytokine production. These cells display pro-inflammatory features, secreting a variety of inflammatory cytokines (*i.e.,* TNF-α, IL-6, IL-1β, and IL-8) and chemokines (*i.e*., MCP-1) after stimulation of cognate cytokine receptors and toll-like receptors (TLR), propagating and sustaining an inflammatory response [10]. However, a lack to resolve such processes and a return to homeostasis results in sustained, low-grade sterile inflammation that appears to be involved in the pathogenesis of DM and cardiometabolic complications, such as CVD [11, 12]. DM phenotypes (*e.g.*, hyperglycemia, insulin resistance, etc*.*) induces inflammation via increasing TLR expression in human monocytes [13]. Monocytes from DM patients also show significantly higher expression levels of *TNF-*α*, IL-6, IL-1, IL-8, COX-2,* and *ICAM-1* compared to healthy individuals [14]. Monocytes are a major source of TNF-α, an inflammatory cytokine involved in systemic inflammation that is induced by lipopolysaccharide (LPS), other bacterial products, and IL-1s [15], suggesting that these immune cells are integral to the inflammatory response and thus a potential target for evaluating its role in DM pathogenesis. Monocyte *TNF*-α gene expression can be induced by high glucose treatment [16], and neutralization of TNF-α improves insulin sensitivity in an animal model of DM [17]. Additionally, the inflammatory states of monocytes from DM patients are modifiable by nutritional factors [18], suggesting a cross talk between lifestyle behaviors that promote DM-related phenotypes and immune activation, wherein inflammation may contribute to DM pathogenesis and exacerbate cardiometabolic complications [19]. However, little is known about the molecular processes and reversibility of inflammatory monocyte phenotypes that might either underlie or is maintained by a DM microenvironment.

Epigenetic modifications, including DNA methylation, are responsive to environmental conditions and influence cellular phenotypes relevant to health and disease [20]. DNA methylation, a reversible epigenetic modification, preferentially occurs at the 5-position carbon of cytosine at a cytosine-guanine dinucleotide (CpG) [21]. Recently, the analysis of DNA methylation states genome-wide in blood cells has helped to facilitate the development of remarkably accurate epigenetic biomarkers relevant to disease [22, 23]. In peripheral blood mononuclear cells (PBMCs), altered global DNA methylation level has been associated with insulin resistance, independent of other risk factors for DM [24], and is the strongest risk factor of CVD mortality [25] and coronary heart disease [26–28]. Inflammation, a potential mechanism involved in DM pathogenesis, was shown to associate with global DNA hypermethylation [29]. Studies examining differential methylation at specific genomic loci in PBMCs have identified changes at genes related to immune function and inflammatory pathways, which were associated with clinical indicators (*i.e.,* C-reactive protein [CRP]) of inflammation [30]. Perturbations to DNA methylation in PBMCs are suspected to influence inflammation due to its role in regulating the expression of genes involved in inflammatory pathways of immune cells [31], which was similarly observed in monocytes [32]. Together, these studies suggest a role for DNA methylation in regulating the pro-inflammatory response of monocytes that may be relevant to DM and its complications.

To better understand the epigenetic processes underlying monocyte inflammation relevant to DM, we examined the genome-wide DNA methylation states of monocytes and their inflammatory activity from NH DM participants undergoing a diabetes-specific social support program (DM-SSP) to maintain diabetes self-management educational goals provided by a priori community-based DM education programs [33, 34]. We identified changes in monocyte-specific DNA methylation and gene transcription states and determined whether these alterations were linked to the improvements in monocyte inflammatory phenotypes in response to the DM-SSP designed as a maintenance program for DM management. By measuring DNA methylation at single-nucleotide resolution, we observed robust differences in methylation in monocytes of participants at baseline compared to post-intervention (after 3 months), particularly at genes relevant to DM progression. DM-SSP-associated alterations in DNA methylation were reflected in changes to the transcriptome, in which DNA methylation was associated with altered transcription, notably at genes involved in the inflammatory response. Further, we observed an attenuated inflammatory response in monocytes following the DM-SSP. Altogether, our results may explain the beneficial effects of a DM-SSP maintenance intervention on inflammation and may have implications for understanding the molecular and cellular processes that underlie DM and its associated comorbidities.

## Results

### Clinical and immunological changes from participants enrolled in a diabetes-specific social support program

**Fig. 1:**
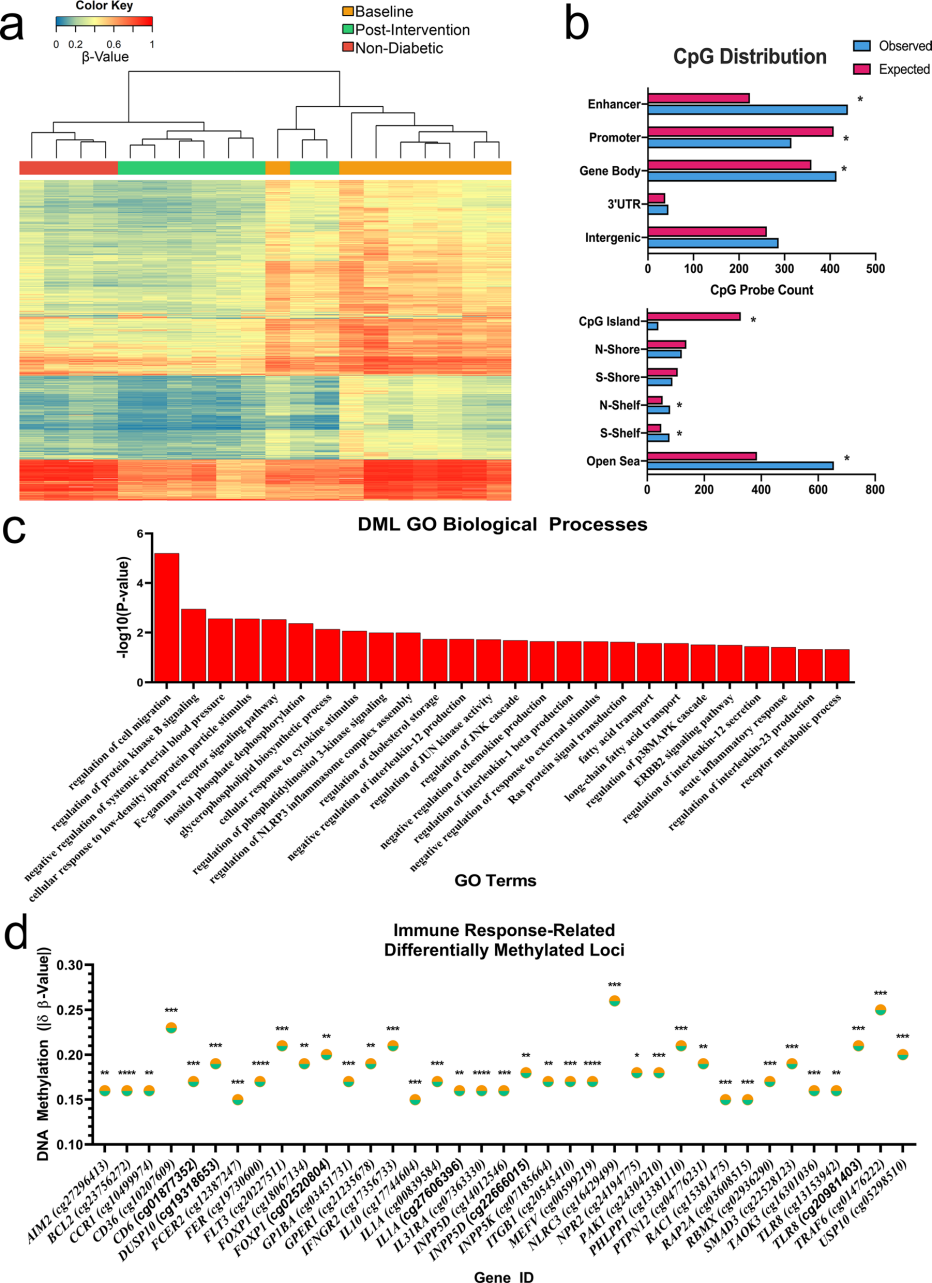
DM-SSP intervention-associated DNA methylation signatures in monocytes. **a** Differentially methylated loci (DML) in monocytes from participants at intervention timepoints, baseline (orange) and post-intervention (green), and among non-diabetic controls (red) identify distinct clusters of methylation patterns from unsupervised hierarchical clustering analysis (Manhattan distance, complete linkage method). Methylation values (β-value) are displayed as a range from low (0, blue) to high methylation (1, red). **b** Plot shows the expected (fuchsia) and observed (blue) CpG probe count for the DML at specific genomic regions (enhancer, promoter, gene body, 3’UTR, and intergenic) and the distribution around CpG Islands (CpG Island, N-shore, S-Shore, N-Shelf, S-Shelf, Open Sea). **c** Gene ontology analysis of DML enriched at the most significant biological processes indicated. **d** Differential DNA methylation between intervention timepoints (δβ-value =|Baseline β-value - post-intervention β-value|) at genes annotated to immune response-related functions. *P*  value of < 0.05, < 0.01, < 0.001, and < 0.0001 indicated by *, **, ***, and ****, respectively. Significance taken at *P* value < 0.05

To address the health disparity of DM in NHs, a DM-SSP for NHs was previously developed and validated [35]. For this study, we recruited NH individuals enrolled in the 3-month DM-SSP intervention by our community partners. From 16 participants, we randomly chose 8 participants for our epigenetic study (Fig. 1). Demographic characteristics of study participants (*n* = 8) at baseline are shown in Table 1. Clinical data collected from participants at baseline and post-intervention are shown in Table 2. To ensure those chosen for our study were representative of the larger enrollment (*n* = 16), we compared clinical characteristics at baseline between our epigenetic study participants (*n* = 8) and the remaining participants from the DM-SSP enrollment (*n* = 8; Additional file 2: Table S1). To ensure homogeneity of monocyte populations for downstream analyses, samples enriched by magnetic cell separation were immunophenotyped to confirm the effectiveness of the enrichment of monocytes from PBMCs pre- and post-intervention, which exhibited robust enrichment (> 70% total monocytes with debris exclusion) at both timepoints (Mean ± Standard Deviation; Baseline = 80.3 ± 10.5, Post-Intervention = 75.7 ± 6.8). Glycemic status (*i.e.*, HbA1c) was not significantly different between baseline and 3 months post-intervention (Baseline = 8.9 ± 1.3, Post-Intervention = 8.7 ± 2.4, Table 2). However, we observed significant, but clinically modest, changes in weight (Baseline = 224.9 ± 37.0 lbs., Post-Intervention = 220.5 ± 35.9, *P* < 0.05), BMI (Baseline = 36.2 ± 5.2 kg/m^2^, Post-Intervention = 35.5 ± 4.9, *P* < 0.05), and Diabetes Care Profile (Baseline = 2.7 ± 0.8, Post-Intervention = 4.0 ± 1.0, *P* < 0.01). Reductions in weight has been shown to have a significant impact on DM risk and management, delaying the onset of DM and improving glycemic control in those at-risk or known to have DM [36]. The Diabetes Care Profile, a validated survey instrument, used to assess the psychosocial factors related to diabetes care, such as diabetes self-management comprehension, has been associated with improved glycemic control [37]. The nominal changes to glycemic parameters (*i.e.*, HbA1c) are not unexpected, given the short timeframe of the study, wherein improvements to the clinical features associated with diabetes may follow the cellular and molecular changes that are linked to long-term improvement in diabetic phenotypes, such as improved inflammation. To investigate this further, we sought to characterize the epigenetic modifications that may underlie improved monocyte inflammatory phenotypes.

**Table 1:** DM-SSP participants demographic characteristics (*n* = 8)

	Baseline
Gender (% Male)	62.5
Ethnicity (% Hawaiian)	100
Education, ≥ High School (%)	100
Age, years, mean (SD)	48.7 (8.5)
Marital status (%)	
Currently married	87.5
Never	12.5
Married/divorced/widow	–
Employment status (%)	
Working	87.5
Looking for work	–
Other (retired)	12.5

**Table 2:** Clinical and immunological characteristics across DM-SSP intervention timepoints (*n* = 8)

	Baseline	Post-intervention
Hemoglobin A1c, mean % (SD)	8.9 (1.3)	8.7 (2.4)
Weight, lbs, mean (SD)	224.9 (37.0)	220.5 (35.9)*
Body mass index, kg/m^2^, mean (SD)	36.2 (5.2)	35.5 (4.9)*
Systolic blood pressure, mmHg, mean (SD)	121.9 (10.2)	132.3 (24.4)
Diastolic blood pressure, mmHg, mean (SD)	76.2 (7.5)	84.9 (10.0)
Lipids, mg/dL, mean (SD)		
Total cholesterol	196.8 (49.0)	176.9 (33.7)
High-density lipoprotein cholesterol	37.7 (7.0)	37.3 (9.2)
Low-density lipoprotein cholesterol	85.3 (23.9)	87.0 (23.8)
Triglycerides	413.4 (258.0)	405.9 (199.6)
Problem areas in diabetes score, mean (SD)	46.7 (20.7)	33.4 (26.8)
Diabetes care profile, mean (SD)	2.7 (0.8)	4.0 (1.0)**
Summary of diabetes self-care activities, mean (SD)	14.3 (4.1)	18.1 (5.1)

Significance taken at *P* < 0.05; indicated by *for < 0.05, **for < 0.01

### Distinct changes in monocyte DNA methylation following a diabetes-specific social support program intervention

To determine the extent to which genome-wide DNA methylation states in monocytes may be modified over the course of the DM-SSP intervention, we first isolated homogenous populations of total monocytes (CD14^+^CD16^−/+^) from each NH participant. To corroborate our immunophenotyping of magnetic bead-enriched monocytes, we compared monocyte-specific methylation patterns [38] with that of the corresponding methylation states of enriched monocytes from participants, resulting in a significant positive correlation at baseline (*r* = 0.85, *P* < 0.0001) and post-intervention (*r* = 0.85, *P* < 0.0001), indicating the sufficient homogeneity of the monocyte populations that we used for downstream DNA methylomic and transcriptomic analyses. Characterization of DM-SSP-associated DNA methylation patterns in monocytes between baseline and post-intervention was performed by filtering for CpG sites with absolute average differences in β-values between pre- and post-intervention timepoints at ≥ 0.15 β-units (δ of the β-value) after applying a resampling-based empirical Bayes approach on our dataset to exclude insignificant differences in DNA methylation [39]. This resulted in 1,061 differentially methylated loci (DML) that exhibited statistically significant and biologically relevant differences in DNA methylation. Unsupervised hierarchical clustering of the DML revealed a strong degree by which the methylation states in monocytes distinguished between baseline and post-intervention (Fig. 1a), indicating robust differential methylation of the 1,061 CpGs across all participants. These findings were consistent with previous reports supporting differential methylation in monocytes as a measure to identify and stratify study populations (*e.g.*, case vs control) [40, 41]. Further, by incorporating DNA methylation data from monocytes of non-diabetic donors (clinical characteristics, Additional file 3: Table S2) at the same 1,061 CpGs into our hierarchical clustering analysis, we observed that the methylation states from non-diabetics clustered with DM-SSP participants post-intervention (Fig. 1a), suggesting a “normalization” of methylation levels to non-diabetic-like states after the intervention. Among DM-SSP-associated DML, we identified 13 hypermethylated (1.23%) and 1,048 hypomethylated (98.77%) loci post-intervention, indicating that a general hypomethylated state was associated with improved outcomes of the intervention. Furthermore, we observed significant mean differences in DNA methylation levels ranging from 15 to 33%, some of which were at CpGs within potential *cis*-regulatory regions. The maximum mean hypomethylated CpG locus post-intervention observed was 33% hypomethylated (in comparison with baseline) at one CpG locus within the 5’UTR of *TRIM34*. The maximum mean hypermethylated locus post-intervention observed was 21% hypermethylated (in comparison with baseline) at one CpG within the 3’UTR of *FNBP1*.

The genomic distribution of methylation associates with its distinct transcriptional regulatory functions. For instance, promoter methylation has been associated with transcriptional silencing [46], whereas gene body methylation is more nuanced and involved with alternative promoter usage and mRNA splicing [47, 48]. The localization of the DM-SSP-associated DML provided insight into the potential regulatory roles. We observed that the DML were significantly enriched at regulatory regions of the genome (Fig. 1b), including enhancers (Observed = 439, Expected = 224, *P* < 0.05) and gene bodies (Observed = 414, Expected = 359, *P* < 0.05); and significantly depleted in promoters (Observed = 315, Expected = 408, *P* < 0.05). In agreement with these observations, we found the distribution of the DML at CpG islands (Fig. 1b), regions dense in clusters of CpG content and typically located at gene promoters [21], to be de-enriched (Observed = 39, Expected = 328, *P* < 0.05). Further, there were significantly more CpGs than expected in open sea regions (Observed = 655, Expected = 385, *P* < 0.5), 5 kb or more from CpG islands, and both the north (N)-Shelf (Observed = 80, Expected = 54, *P* < 0.05) and south (S)-Shelf regions (Observed = 78, Expected = 49, *P* < 0.05) that flank CpG shores (≤ 2 kb from CpG islands) and extending outwards. These data suggested that the DM-SSP-associated DML were enriched at regions of the genome that may have *cis*-regulatory functions.

GO analysis, using Enrichr (https://amp.pharm.mssm.edu/Enrichr/) [51, 52], was applied to the DML to infer the potential cellular and biological processes. Our results revealed that the DML were enriched at genes annotated to biological processes related to DM and DM-related complications, including immune-related functions (*e.g.*, cell migration, cytokine responses, etc.), metabolic processes (*e.g.*, regulation of protein kinase B, regulation of PI3K, etc.), and cardiovascular pathways (*e.g.*, regulation of cholesterol storage, regulation of arterial blood pressure, etc*.*) (Fig. 1c; full list in Additional file 4: Table S3). The enrichment of GO terms at cellular processes linked to DM and DM complications led us to suspect that the DML may contain a subset of CpGs that were localized at genes annotated to cardiometabolic diseases. Indeed, a subset of DML were located at genes associated with hypertension, myocardial infarction, stroke, insulin resistance, coronary artery disease, and DM (Additional file 4: Table S3). Finally, due to the contribution of inflammation in DM and its related cardiometabolic complications, we focused on a subset of 37 DML enriched at genes annotated to the immune response (Fig. 1d). Differential methylation analysis (δ β-Value =|Baseline β-Value - Post-intervention β-Value|) at this subset of CpGs revealed an absolute difference in methylation levels between pre- and post-intervention that ranged from 15 to 26% (Fig. 1d; Additional file 4: Table S3). An intragenic CpG (cg16429499) of *NLRC3,* a gene previously identified as having a role in regulating inflammation [53], exhibited the most robust differential methylation states between timepoints. Thirty-six of the 37 DML (97.2%) were characterized by DNA hypomethylation post-intervention compared to baseline (Additional file 4: Table S3). These results indicated DM-SSP-associated DML may functionally contribute to cellular processes relevant to DM (*e.g.*, inflammation, insulin signaling pathways, etc.).

### Diabetes-specific social support program-associated changes in monocyte gene expression may be epigenetically regulated

**Fig. 2:**
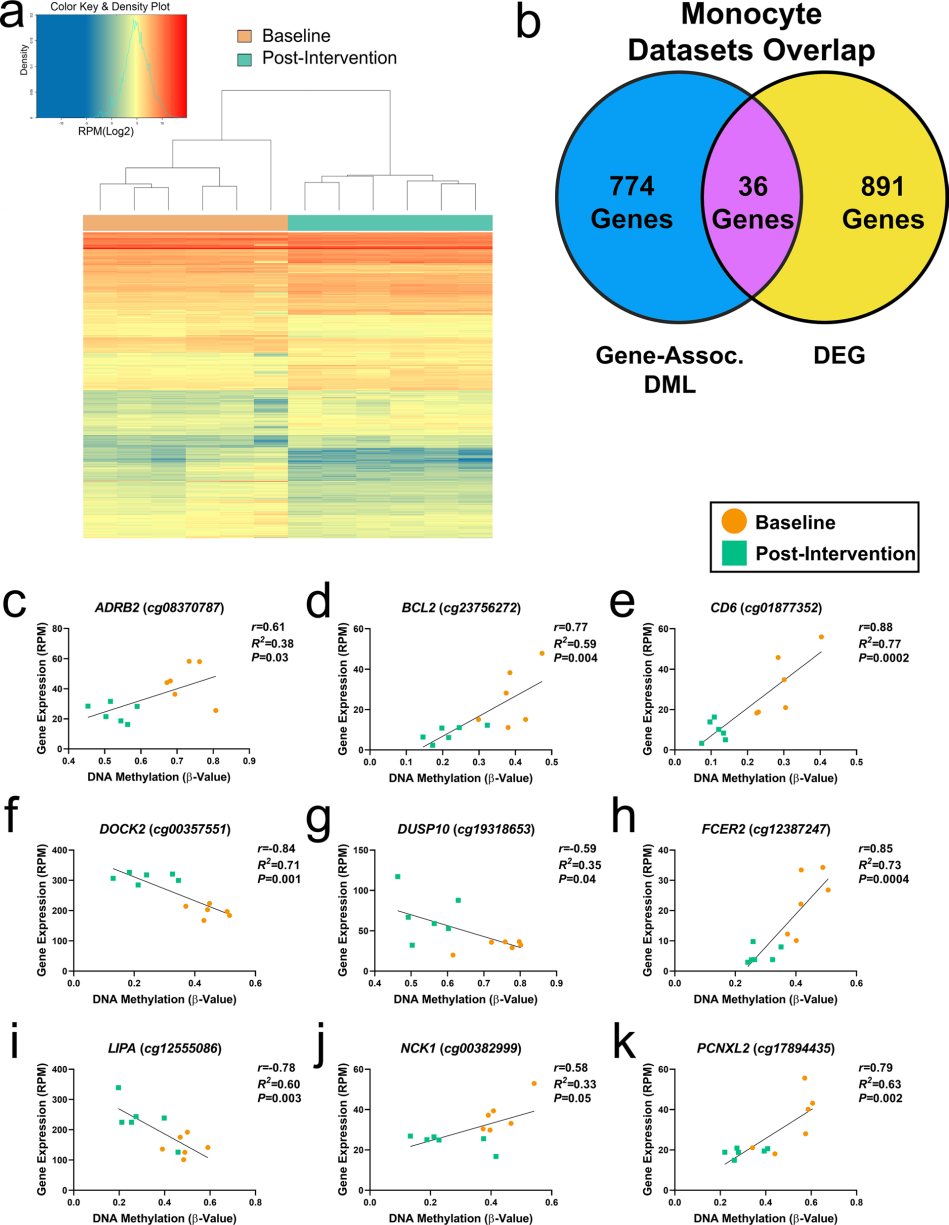
DM-SSP intervention-associated differential gene expression overlaps DML. **a** Heatmap of Log_2_ (RPM) of the differentially expressed genes (DEGs) by unsupervised hierarchal clustering show two main clusters between baseline (orange) and post-intervention (green). RPM, reads per million reads. **b** Venn diagram of DML dataset at known genes (blue), DEGs (yellow), and overlap between CpGs-enriched at DEGs in both (purple). **c**–**k** Plots display correlation between methylation (x-axis) and gene expression (y-axis) from baseline (orange) and post-intervention (green) for a subset of overlapping CpG-enriched genes (Fig. 3b) annotated to the immune response, metabolic processes, cardiovascular processes, and cardiometabolic diseases, including **c** *ADRB2*, **d** *BCL2*, **e** *CD6*, **f** *DOCK2*, **g** *DUSP10*, and **h** *FCER2*, **i** *LIPA*, **j** *NCK1*, and **k** *PCNXL2*. *ADRB2*: adrenoceptor beta 2; *BCL2*: BCL2 apoptosis regulator; *CD6*: cluster of differentiation 6; *DOCK2*: dedicator of cytokinesis 2; *DUSP10*: dual specificity phosphatase 10; *FCER2*: Fc fragment of IgE receptor II; *LIPA*: lipase A; *NCK1*: NCK adaptor protein 1; *PCNXL2*: pecanex-like protein 2. Significance taken at *P* value < 0.05

To examine whether DM-SSP-associated methylation differences may relate to transcription [54], we first identified differentially expressed genes (DEGs) in monocytes pre- and post-intervention in a subset of participants, which revealed 891 significant DEGs (*P*_*FDR*_ < 0.05). Unsupervised hierarchical clustering of log-transformed gene expression data (Reads Per Million reads [RPM]) of the 891 DEGs revealed a strong degree by which gene expression partitioned both timepoints (Fig. 2a), consistent with a previous report examining differential expression in monocytes stratifying disease states [40]. GO pathway analysis of the DEGs revealed significant enrichment of genes relevant to the immune response, metabolic processes, and cardiovascular pathways (Additional file 5: Table S4). That these pathways were also revealed by the DML suggest these robust differences in gene expression may be epigenetically regulated. To explore this further, we integrated the gene-enriched DML (774 CpGs) with DEGs (891 genes) and observed 36 genes that overlapped both datasets (Fig. 2b). Twelve of the 36 DML-enriched genes were involved in the immune response, metabolic processes, or cardiovascular pathways, and cardiometabolic diseases (*ADRB2, BACH2, BCL2, CD6, DOCK2, DUSP10, FCER2, GP1BA, ITGB1, LIPA, NCK1,* and *PCNXL2*). To examine whether these DML-enriched genes may be epigenetically regulated, we performed correlation analyses between the methylation and expression states pre- and post-intervention. We observed significant associations between DNA methylation and gene expression at 9 out of the 12 DML-enriched genes, including *ADRB2* (*cg08370787*, *r* = 0.61, *P* = 0.03, Fig. 2c), *BCL2* (*cg23756272*, *r* = 0.77, *P* = 0.004, Fig. 2d), *CD6* (*cg01877352*, *r* = 0.88, *P* = 0.0002, Fig. 2e), *DOCK2* (*cg00357551*, *r* = − 0.84, *P* = 0.001, Fig. 2f), *DUSP10* (*cg19318653*, *r* = − 0.59, *P* = 0.04, Fig. 2g), *FCER2* (*cg12387247*, *r* = 0.85, *P* = 0.0004, Fig. 2h), *LIPA* (*cg12555086*, *r* = − 0.78, *P* = 0.003, Fig. 2i), *NCK1* (*cg00382999*, *r* = 0.58, *P* = 0.05, Fig. 2j), and *PCNXL2* (*cg17894435*, *r* = 0.79, *P* = 0.002, Fig. 2k). Among those with significant relationships between DNA methylation and gene expression, four CpGs were enriched at genes with known involvement in immune-related processes (*i.e.*, cellular response to cytokine stimulus, regulation of JNK cascade/activity, acute inflammatory response, etc*.*): *BCL2*, *CD6*, *DUSP10*, and *FCER2*. These findings suggest these DML in epigenetically labile *cis*-regulatory regions involved in transcriptional regulation.

### A diabetes-specific social support program associates with attenuated pro-inflammatory responses from monocytes

**Fig. 3:**
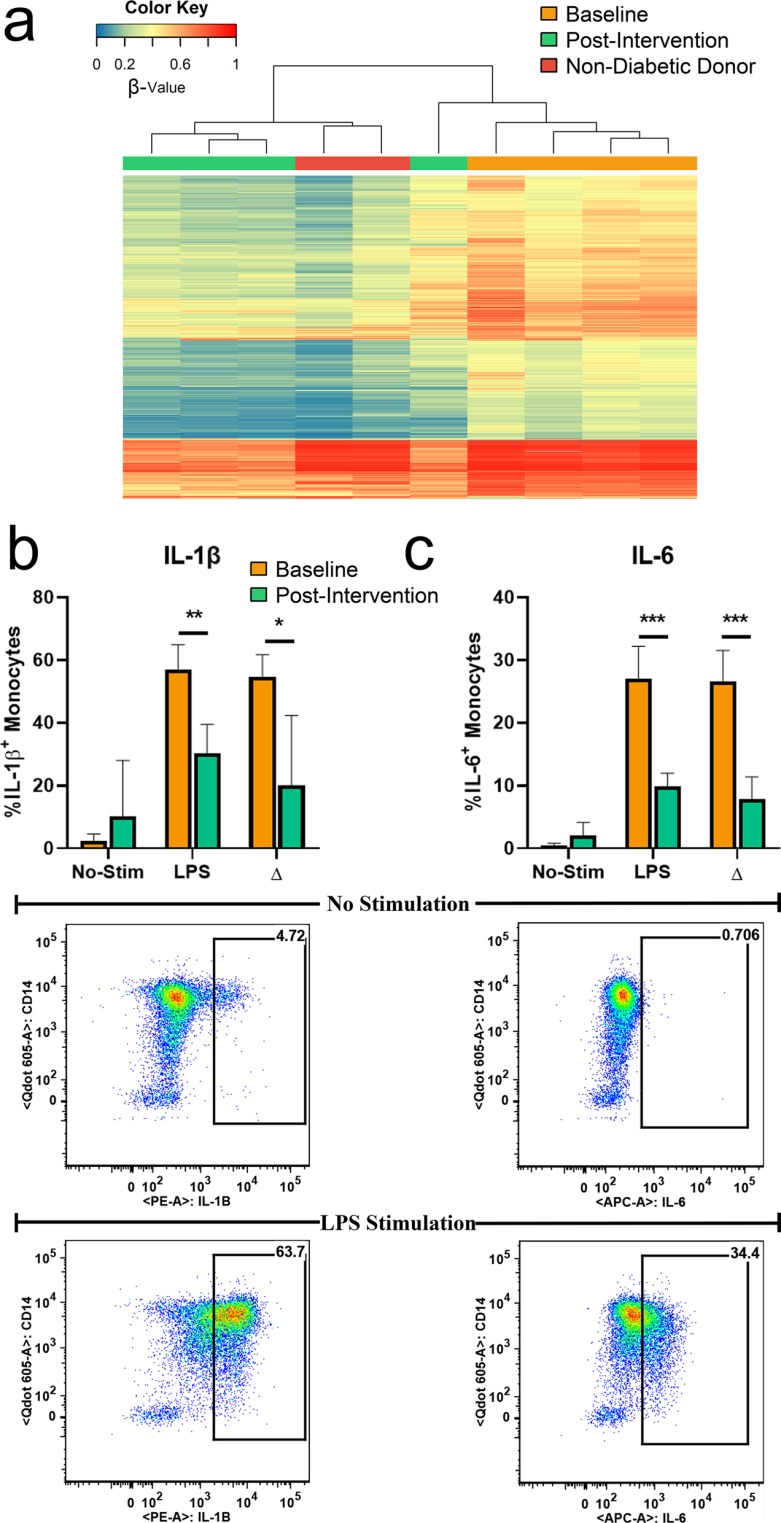
Changes in monocyte inflammatory response between intervention timepoints determined by monocyte intracellular cytokine staining (Mono-ICS). **a** Unsupervised hierarchical cluster analysis (Manhattan distance, complete linkage method) of the DML from a subset of participants subjected to Mono-ICS. **b**–**c** Figures represent the frequency of monocytes producing inflammatory cytokines at baseline (orange) and post-intervention (green) for **b** IL-1β, **c** IL-6 for two conditions: non-stimulated (No-Stim) and stimulation with inflammatory stimuli (LPS). Δ represents the difference in the frequency of cytokine-producing monocyte between LPS and No-Stim conditions. IL-1β, interleukin 1-beta; IL-6, interleukin 6; LPS, lipopolysaccharide. Bottom figures represent gating strategy employed for quantifying the frequency (%) of cytokine secreting monocytes from both treatments for **b** & **c.** *P* value for < 0.05, < 0.01, < 0.001, indicated by *, **, ***, respectively

Given the enrichment of DM-SSP-associated differences in methylation and expression at genes involved in inflammation, we determined whether participation in DM-SSP led to changes in inflammatory states of monocytes. Thus, we performed monocyte intracellular cytokine staining (Mono-ICS), an ex vivo immunophenotyping assay used to determine monocyte inflammatory activity in response to the inflammatory stimuli lipopolysaccharide (LPS), in the same aliquot of monocytes used for molecular analyses. We selected 4 participants at baseline and 4 post-intervention (3 were matched participants at both timepoints), all of whose samples were included in our methylomic and transcriptomic profiling analyses. We first performed unsupervised hierarchical clustering analysis of the initial DM-SSP-associated DML (1,061 CpGs) in monocytes from this subset of participants, which robustly stratified individuals from both timepoints (Fig. 3a). Next, we measured inflammatory cytokine (IL-1β, IL-8, IL-6, and TNF-α) production from monocytes in resting conditions and stimulated with LPS. In an inflammatory stimuli-free condition (no-stim), participants displayed a low percentage of cytokine-specific producing monocytes (% of cytokine-specific^+^ monocytes/% of total monocytes) from both timepoints. Upon stimulation with LPS, we observed increased production of cytokine-producing monocytes at pre- and post-intervention relative to their respective stimuli-free conditions. However, comparing pre- to post-intervention timepoints under the LPS stimulated conditions, pre-intervention samples exhibited a significantly higher frequency of IL-1β (Baseline = 56.93 ± 7.96%, Post-Intervention = 30.33 ± 9.17%, *P* = 0.005, Fig. 3b) and IL-6 (Baseline = 26.98 ± 5.22%, Post-Intervention = 9.89 ± 2.06%, *P* = 0.001, Fig. 3c) producing monocytes than that of post-intervention. While these results are in agreement with previous reports suggesting that the beneficial effects of a diabetes self-management intervention include the reduction of inflammation and improved glucose tolerance [55], to our knowledge this attenuated monocyte-specific inflammatory response post-DM-SSP has never before been reported.

We confirmed whether there was a heightened, or “hyper-responsive,” monocyte immune response to inflammatory stimuli pre-intervention that may have been attenuated post-intervention. We did this by comparing the difference in the frequency (%) of cytokine-producing monocytes between LPS-stimulated (LPS) and stimuli-free (no-stim) conditions (LPS - no-stim = Δ-value) separately at each intervention timepoint (Fig. 3b, c). Indeed, we observed a hyper-responsiveness to LPS challenge at pre- versus post-intervention for IL-1β (Baseline = 54.57 ± 7.13%, Post-Intervention = 20.10 ± 22.17%, *P* = 0.03, Fig. 3b) and IL-6 (Baseline = 26.54 ± 5.02%, Post-Intervention = 7.82 ± 3.55%, *P* = 0.001, Fig. 3c). That the overlapping DM-SSP-associated differences in DNA methylation and expression are at genes involved in the immune response, coupled with the attenuated inflammatory activity post-intervention, together suggest that DM-SSP facilitates epigenetic modulation that underlies monocyte function.

### Epigenetic regulation of inflammatory genes associates with inflammatory monocytes

**Fig. 4:**
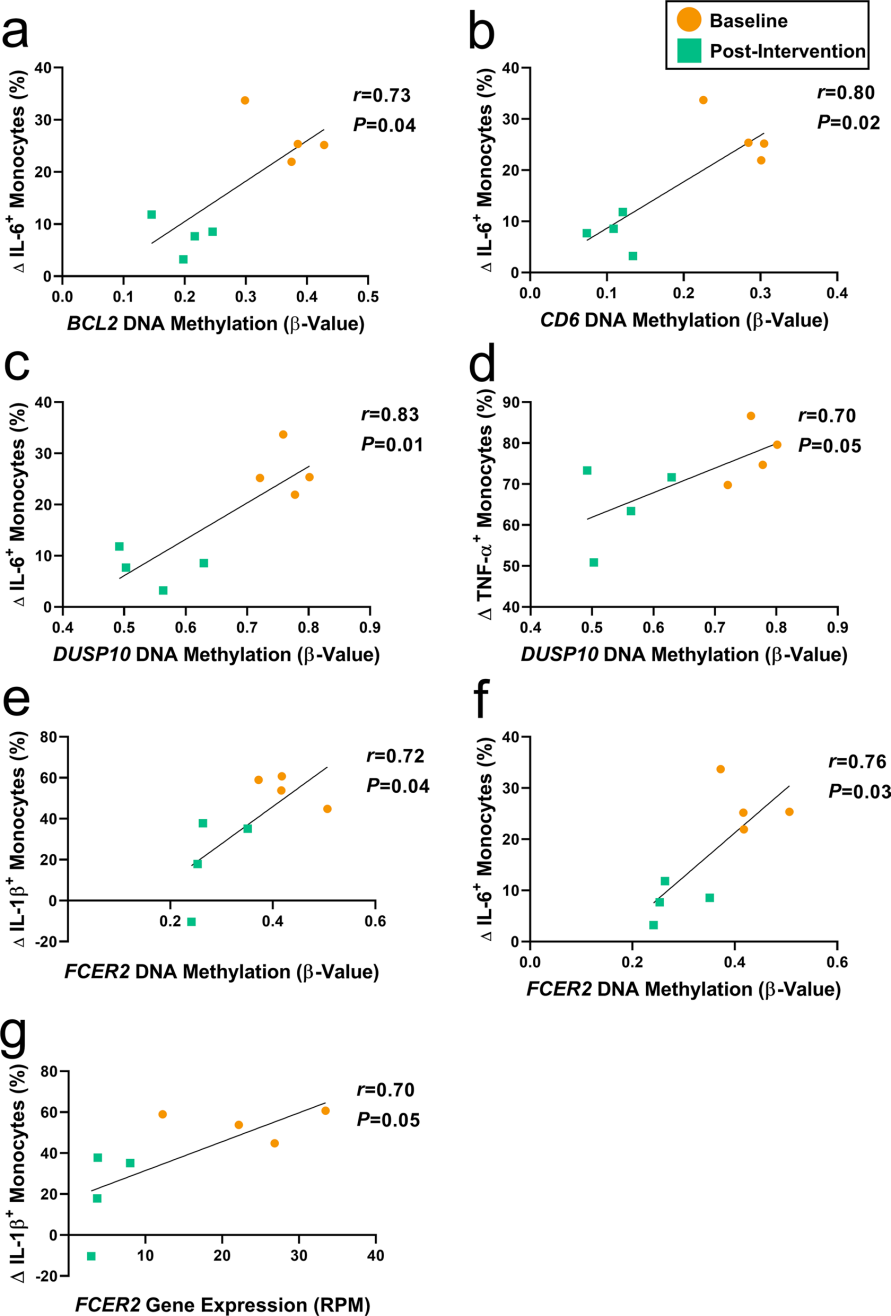
Association between monocyte immune responses and immune-related gene differential methylation. Plots represent correlation between the delta (Δ) frequency (%) of cytokine-producing monocytes (LPS-stimulated frequency of cytokine-producing monocytes [%] - no stimulation frequency of cytokine-producing monocytes [%]; y-axis) and **a**–**f** DNA methylation (β-value; x-axis; left) and **g** gene expression levels (RPM; x-axis; right) from baseline (orange circle) and post-intervention (green squares) samples for the subset of immune-related DML and DML-enriched DEG, including between **a** *BCL2* & IL-6, **b** *CD6* & IL-6, **c** *DUSP10* & IL-6, **d** *DUSP10* & TNF-α, **e** *FCER2* & IL-β, **f** *FCER2* & IL-6, **g** *FCER2* & IL-1β. Significance taken at *P* value < 0.05

Previous data has shown that DNA methylation may facilitate pro-inflammatory responses to inflammatory stimuli [57]. As most, if not all, circulating monocytes would have turned over during the course of a 3-month intervention, the epigenetic, transcriptional, and functional differences we observed are likely a result of changes to the monocyte trajectory during differentiation. Thus, we focused on identifying a relationship between the immune response in monocytes and the monocyte methylation states of our previously observed immune-related DML that appear to be transcriptionally regulated (*BCL2*, *CD6*, *DUSP10,* and *FCER2*). From the same subset of participants used to assess monocyte inflammatory response, we compared Mono-ICS inflammatory response data (Δ % of cytokine-producing monocytes = LPS % of cytokine-producing monocytes - no stim % of cytokine-producing monocytes) to the methylation states of the DML at each timepoint. For an intragenic DML of *BCL2* (*cg23756272*), monocyte methylation displayed a significant relationship with the frequency of cytokine-producing monocytes in response to LPS challenge for IL-6 (*r* = 0.73, *P* = 0.04, Fig. 4a). The association between methylation of an exon-localized DML of *CD6* (*cg01877352*) was significantly correlated with IL-6^+^ monocytes (*r* = 0.80, *P* = 0.02, Fig. 4b). *DUSP10*, containing an intragenic DML (*cg19318653*), showed a significant positive association between DNA methylation and IL-6 (*r* = 0.83, *P* = 0.01, Fig. 4c) and TNF-α-secreting monocytes (*r* = 0.70, *P* = 0.05, Fig. 4d). We observed that the methylation state of the promoter-localized DML of *FCER2* (*cg12387247*) was significantly positively correlated with both IL-1β^+^ (*r* = 0.72, *P* = 0.04, Fig. 4e) and IL-6^+^ (*r* = 0.76, *P* = 0.03, Fig. 4f) monocytes in response to LPS stimulation. Finally, we sought to investigate whether gene expression of each immune-related DML was associated with the cytokine-producing monocytes. Our results showed that the expression levels of only *FCER2* was significantly associated with IL-1β^+^ monocytes (*r* = 0.70, *P* = 0.05, Fig. 4g). Together, these results revealed that the methylation and immune response states were dynamic between intervention timepoints, indicating that they were responsive to the DM-SSP intervention. These results suggest that the apparent modulation of DNA methylation states of genes associated with the inflammatory response of monocytes (*i.e., BCL2*, *CD6*, *DUSP10*, and *FCER2*) may be the result of a shift in the monocyte differentiation trajectory as the cell population replenishes over the course of the 3-month intervention.

## Discussion

Monocytes respond rapidly and dynamically to environment signals, polarizing to various cellular phenotypes in both physiological and pathophysiological conditions. Pathophysiological features of diabetes, such as hyperglycemia, have been linked to shifts in monocyte inflammatory phenotypes [58]. This plasticity is facilitated by epigenetic modifications, including DNA methylation [59]. We observed a strong degree by which DNA methylation states in monocytes was able to delineate pre- and post-intervention conditions, independent of clinical, psychosocial, or immunological characteristics. Previous work by our group has shown the utility of differentially methylated patterns in monocytes as an indicator of diseased states [40, 41]. Additionally, including the methylation states at the same loci from monocytes collected from healthy individuals showed that the DNA methylation landscape reflected that of a non-diabetic-like state post-intervention. This “normalized” epigenetic landscape may be modified in response to distress, which may be involved in the lower inflammatory state of monocytes in diabetic individuals prior to improved glycemic control [60]. Together, these results support the application of epigenome profiling as a novel approach to evaluate the efficacy of a DM-SSP intervention on potential long-term health outcomes. Indeed, our findings that DNA methylation changes may precede measurable clinical changes in disease pathology are akin to measurements of pre-cancerous tissue [61]. A recent study by Ouni and colleagues identified differentially methylated regions and differentially expressed genes in the islets of Langerhans from obese mice that preceded pancreatic dysfunction and future diabetes [62]. Similarly, we observed DM-SSP-associated changes in differentially methylated loci in the DM individuals irrespective of glycemic improvements, which suggests that the DML modifications may occur prior to changes in disease phenotypes.

The dynamic changes in monocyte DNA methylation states appear to be involved in the transcriptional regulation of genes that shape monocyte activity. Indeed, most of the changes to DNA methylation we observed post-intervention were at CpGs enriched in intragenic regions of the genome where they may be involved in mediating *cis*-regulatory functions, such as enhancer usage [63], alternative promoter control and alternative splicing [48, 49], which together may be linked to aberrant cellular function and diseased states [50]. Among the differentially methylated CpG loci, we observed a general hypomethylated state in monocytes after the intervention, which is consistent with previous observations of healthy lifestyle habits (*e.g.*, physical activity) associated with hypomethylation and reduced risk for DM [42], and global DNA hypermethylation associating with DM [43]. The most distinct differences in methylated DNA levels post-intervention were observed at CpGs associated with *TRIM34* (hypomethylated) and *FNBP1* (hypermethylated). In addition to a causal role in modulating cholesterol [44], *TRIM* family members including *TRIM34* have a role in macrophage innate immunity via induction by toll-like receptor-3 and receptor-4 ligands [64]. *FNBP1* has been reported to contribute to the formation of the phagocytic cups of macrophages [45] and has been identified as a conserved gene involved in mononuclear phagocyte subset identification in animal models [65]. Coupled with these examples, we observed that DM-SSP-associated DMLs were enriched at genes implicated in the immune response, insulin signaling, glucose and lipid metabolism, and cardiometabolic disease, including type 2 diabetes mellitus. Thus, our results suggest an immunoepigenetic signature relevant to diabetes pathophysiology in monocytes. This observation implicates specific genomic regions in monocytes susceptible to epigenetic plasticity and may underlie inflammation and cardiometabolic outcomes in individuals with non-DM [59].

The changes to the monocyte DNA methylation landscape that preferentially occurred over functionally relevant regions of the genome prompted us to examine gene expression. Consistent with gene pathway analysis of DM-SSP-associated DMLs, most differentially expressed genes were attributed to biological processes involved in immune-related pathways, including activator protein 1 (AP-1) and hypoxia-inducible factor-1 (HIF-1) transcription networks. This result suggested that altered transcription factor networks may mediate the changes to expression of inflammatory genes [66–68]. Further, we identified differentially expressed genes that contained at least one differentially methylated locus. A subset of these overlapping loci occurred at genes involved in inflammatory processes, including *BCL2*, *CD6*, *DUSP10*, and *FCER2*. Antagonism of *BCL2*, a pro-survival protein, was reported to promote both glucose signaling and insulin secretion in pancreatic β-cells [69], and may be involved in β-cell apoptosis [70], potentially contributing to loss of β-cell function and subsequently impaired glucose homeostasis typically observed in diabetic patients. Further, monocytes expressing *BCL2* have attenuated inflammatory responses, reduced differentiation, and activation of macrophages [71]. CD6, a lymphocyte receptor, is described in T cell activation and acts as a co-stimulatory receptor for monocyte antigen presentation [72]. Although administration of anti-CD6 antibodies in combination with oral insulin was shown to be protective of diabetes in new onset diabetes in mice [73], the function of CD6 in monocytes has not been described. *DUSP10* has been previously described as an important protein involved in regulating inflammation [74], possibly through regulating MAPK proteins p38 and c-Jun N-terminal kinase, and has been associated with inflammation-associated diseases, such as diabetes and associated cardiovascular disease [75]. FCER2, also known as CD23, is found in various hematopoietic-derived immune cells; in monocytes, it is known to induce inflammatory cytokines [76]. Further, activation of CD23^+^ monocytes causes its differentiation toward antigen-presenting macrophage phenotypes [77]. Our data indicate that these genes are likely to be regulated by epigenetics and may be involved in shaping monocyte differentiation and function.

Modified lifestyle behaviors are associated with attenuated inflammation and improved metabolic outcomes and disease risk [17, 78]. Recent and accumulating evidence have indicated epigenetic mechanisms are involved in inflammatory processes linked to diabetes and cardiometabolic complications [59, 79]. Given that the epigenetic and expression changes we observed occurred at genes involved in inflammation, we hypothesize that monocytes pre- and post-intervention may be differentially poised for an inflammatory stimuli response. Using ex vivo monocyte cytokine immunophenotyping assays on the same set of monocytes we had molecularly profiled, we observed robust differences in the inflammatory response of cells from baseline to 3 months post-DM-SSP intervention in our cohort of NHs with DM, which was further corroborated with the observed differences in the responsiveness of monocytes to the inflammatory stimuli, lipopolysaccharide. Du et al*.* has previously observed a monocyte hyper-responsiveness to LPS and lipoteichoic acid, in which inflammatory stimulation led to significant increases in the production of IL-1β and TNF-α in DM patients and latent autoimmune diabetics relative to healthy controls [56]. Together, these results indicated that compared to pre-intervention, monocytes of diabetic individuals post-intervention had a significantly attenuated inflammatory response. It is plausible that along with an increase in the frequency of cytokine-producing monocytes upon challenge with inflammatory stimuli (*i.e.*, LPS) there is a robust responsiveness, or trained immunity, in monocytes to inflammatory stimuli constantly present in the microenvironment of individuals with known DM (*e.g.*, oxidized LDL, LPS, free fatty acids, hyperglycemia, etc.) [80], which together may actively participate in maintaining and propagating diabetic phenotypes. Likewise, the observed reduction of cytokine-producing monocytes and attenuated responsiveness of monocytes to inflammatory stimuli after the completion of a DM-SSP may, in part, be due to a change in the microenvironment of DM patients.

We reason that chronic exposure of monocytes to a diabetic milieu may impart a hyperinflammatory memory, or trained immunity, mediated by the epigenome. This may augment their ability to elicit a secondary response to inflammatory mediators within the periphery (*e.g.*, glucose, lipids, cytokines, etc.) [81, 82]. Monocytes are characterized by a frequent turnover rate (1–3 days) where in response to environmental cues, hematopoietic stem cell-derived monocytes and monocyte-derived macrophages will polarize to inflammatory or anti-inflammatory states [58, 83, 84]. Interestingly, macrophage phenotypes also appear to facilitate hematopoietic stem cell reprogramming of differentiation toward specific cell fates [85]. For instance, in conditions characterized by inflammatory states, such as diabetes and cardiovascular disease, the hematopoietic stem cell pool continually drives the production of cell types necessary to sustain the inflammatory demand [86]. Further, prior findings by our group have hinted at the potential origins of monocyte epigenetic biomarkers associated with insulin resistance as derived from residual epigenetic states of hematopoietic stem cells [41]. The altered inflammatory state of monocytes in response to the DM-SSP intervention may have been due to shifts in hematopoietic stem cell trajectories in differentiation toward either a resting or anti-inflammatory monocyte phenotype. Likewise, we posit that changes to the monocyte phenotype  may have occurred in response to improved weight management and the psychosocial factors associated with diabetes care, such as the Diabetes Care Profile. Epigenetic signatures have been observed in the complex interactions between nutrition, weight management, obesity, diabetes, and immune cell phenotypes [87]. Psychosocial distress has also been associated with altered inflammation [60]. Further, evidence in pregnant mothers has shown that maternal distress alters immune outcomes in the developing fetus via epigenetic alterations [88]. It is possible the improved feelings of DM-related distress, indicated by changes in Diabetes Care Profiles, could have led to behavioral-induced alterations to monocyte epigenetic profiles underlying immune activity. We hypothesize that behavioral changes and weight management may induce changes to the epigenetic landscape in monocytes to improve inflammatory phenotypes, which may have potential impact on long-term changes in diabetes care and management.

Our pilot study suggests that a DM-SSP intervention contributes to immediate beneficial outcomes on DM management by altering epigenetic modifications to inflammatory genes that may underlie shifts in immune cell phenotypes. Native Hawaiians have a higher incidence of DM compared to the general population, which likely derives from a multifaceted interplay of social and behavioral determinants that influence lifestyle behaviors such as diet, exercise, smoking, alcohol consumption, *etc*., that modify individual-level risk of DM [89]. We reason, however, that independent of factors underlying the increased risk of DM in Native Hawaiians, attenuated inflammation, facilitated by the DM-SSP intervention, may be effective in DM management of other populations [90]. For instance, “inflammaging”, a concept defined by the natural increase in age-associated pro-inflammatory states observed in the aging population, has been associated with the increased risk of age-associated diseases including DM [91]. Thus, targeting inflammation may attenuate DM phenotypes in an aging population [92]. Similarly, some drugs administered for DM, including metformin and pioglitazone, have secondary effects on attenuating sterile inflammation, suggesting inflammation as a key target in DM management and prevention [93]. We suspect that individuals living with DM, irrespective of ethnicity, may benefit from a DM-SSP intervention by improvements in psychosocial behaviors that may cause epigenetic changes underlying shifts in inflammatory phenotypes. Whether the intervention-associated differentially methylated loci observed in this pilot study are altered in a broader Native Hawaiian community and the general population living with DM was not within the scope of this study and warrants further investigation. Nonetheless, our pilot study adds an additional layer to understanding potential mechanisms of DM self-management on DM maintenance, while providing evidence for the inclusion of inflammatory markers in evaluating DM maintenance-focused interventions and DM management. Likewise, the reversibility in DNA methylation signatures provides a feasible therapeutic target to abrogate inflammation derived from pro-inflammatory innate immune cell phenotypes, a possibility future studies may seek to address. We acknowledge a major limitation of this pilot study was the small sample size (*n* = 8), which may introduce bias, and results presented here should be interpreted with caution and need further replication in a larger sample size and across different populations living with DM. However, our intent in this exploratory study was to generate hypotheses rather than test it. Our preliminary findings describe potential for further investigation as we have identified a significant shift in the immunoepigenomic landscape of monocytes even in the setting of non-significant glycemic changes. Further, the short timeframe of the DM-SSP, coupled with a minimally invasive measure of glycemic control (HbA1c) also limited our identification of changes in glycemic control, given HbA1c is a stable marker that averages glycated hemoglobin for up to 3 months. Although beyond the scope of this study, future studies may seek to address changes in participant’s clinical phenotypes several months after an intervention and a longer timeframe to observe changes in stable glycemic markers, such as HbA1c. Additionally, although significant improvements in glycemia were not observed, participants displayed robust changes in inflammatory responses, which indicated the possibility that the modifications to DNA methylation, transcription, and inflammatory phenotypes observed may have occurred independent of changes to glycemic control and suggests that inflammation may indeed precede clinically relevant changes in DM; however, we cannot rule out the possibility of unmeasured factors contributing to the altered DNA methylome, transcriptome, and immune response. Additional limitations of this study include the scope of molecular changes observed, directionality of relationships identified, and monocyte enrichment method. DNA methylation analyses were performed with a single-nucleotide resolution microarray-based platform with limited coverage of CpGs (less than 2% of the CpG content throughout the genome). As such, other potential genomic regions involved in epigenetic regulation of monocyte activity were likely missed; however, the limited CpG content that was surveyed may yet reflect changes to broader regional methylation patterns [94]. Whole-genome bisulfite sequencing methods [95] will overcome this limitation in future as it becomes increasingly more amenable to clinical studies. Another limitation involves the relationship between methylation, transcription, and monocyte inflammatory responses we observed; while the associations we examined may indicate direct roles of methylation in regulating gene expression and inflammatory activity of monocytes, we cannot rule out the possibility that indirect mechanisms may contribute to the changes we observed in monocyte phenotype. Indeed, we found methylation-associated changes in transcription factors involved in inflammation that may act upstream to regulate the expression of genes we observed to be differentially expressed post-intervention. These findings warrant further investigation. In addition, although a relatively homogenous population of cells, monocytes can further be distinguished into several subtypes [96] and evidence supports a spectrum of monocytes with varied function [97]. Although a widely used benchmark methodology, our monocyte enrichment assays are based on negative selection of specific cell surface markers that does not distinguish these subtypes. However, we posit that the robust DM-SSP-associated changes in methylation and expression are likely a result of the major subtype within the monocyte population, classical monocytes (CD16^+/++^CD14^−)^. This reinforces the notion of a shift in the monocyte differentiation trajectory post-intervention to account for the molecular differences we observed, and can be resolved in future studies using more advanced single cell-based methodologies [98]. Nonetheless, our pilot study is the first to examine changes in the DNA methylation, transcription, and inflammation states of specific innate immune cell populations from individuals participating in a DM-SSP intervention. Despite the limitations noted, our findings set a precedent for future studies, including expansion of epigenome profiling of a larger cohort of Native Hawaiians and other individuals undergoing DM self-care interventions, to better understand the molecular and cellular impacts of these interventions and to identify functionally relevant gene pathways as targets for improving and/or monitoring the potential long-term benefits of such interventions.

## Conclusions

Native Hawaiians diagnosed with DM were found to have significant changes to the epigenetic landscape of monocytes at regions of the genome involved in their inflammatory activity following a diabetes-specific social support program intervention. Improvements in Diabetes Care Profile following the DM-SSP intervention were associated with an epigenetic-based shift in monocyte inflammatory activity defined as a reduced state of systemic inflammation irrespective of corresponding improvements in glycemic status (HbA1c) and with only modest improvements in weight and systolic blood pressure.

## Methods

### Participant enrollment from a diabetes-specific social support program (DM-SSP)

All participants were recruited from a pre-existing study aimed at testing a DM-specific social support program (DM-SSP) following a standard diabetes self-management education program focused on reducing risk factors associated with diabetes complications, known as “Partners in Care (PIC),” described elsewhere [35]. Clinical measurements were taken at baseline (t_0_) and post-DM-SSP (t_1_ = 3 months), which included weight, height, BMI, HbA1c, total cholesterol, HDL cholesterol, LDL cholesterol, triglycerides, and diastolic and systolic blood pressure. Secondary outcomes to the DM-SSP intervention used to assess DM self-management comprehension, performance on self-management activities, and DM-related stress were collected using the Diabetes Care Profile, Summary of Diabetes Self-Care Activities, and Problem Areas in Diabetes, respectively. The Diabetes Care Profile uses a questionnaire consisting of 12 questions related to diabetes self-care, each scaled from 1 (poor) to 5 (excellent), with an overall score ranging from 12 (poor understanding of diabetes self-management) to 60 (excellent understanding) [37]. The Summary of Diabetes Self-Care Activities (SDSCA) consists of a survey determining whether a participant followed a DM self-care routine within the last week by indicating the total number of days he/she performed activities relating to DM self-care; results were averaged across 7 activities and scores ranged from 7 (no weekly DM self-care activities) to 28 (DM self-care activities every day) [99]. DM-related distress included a 20-item questionnaire to assess feelings toward living with DM and DM-related treatment using a Likert scale from 0 (not a problem) to 4 (serious problem), and scores were combined across all 20 questions and multiplied by 1.25; higher scores correspond to greater DM-related distress [100].

**Fig. 5:**
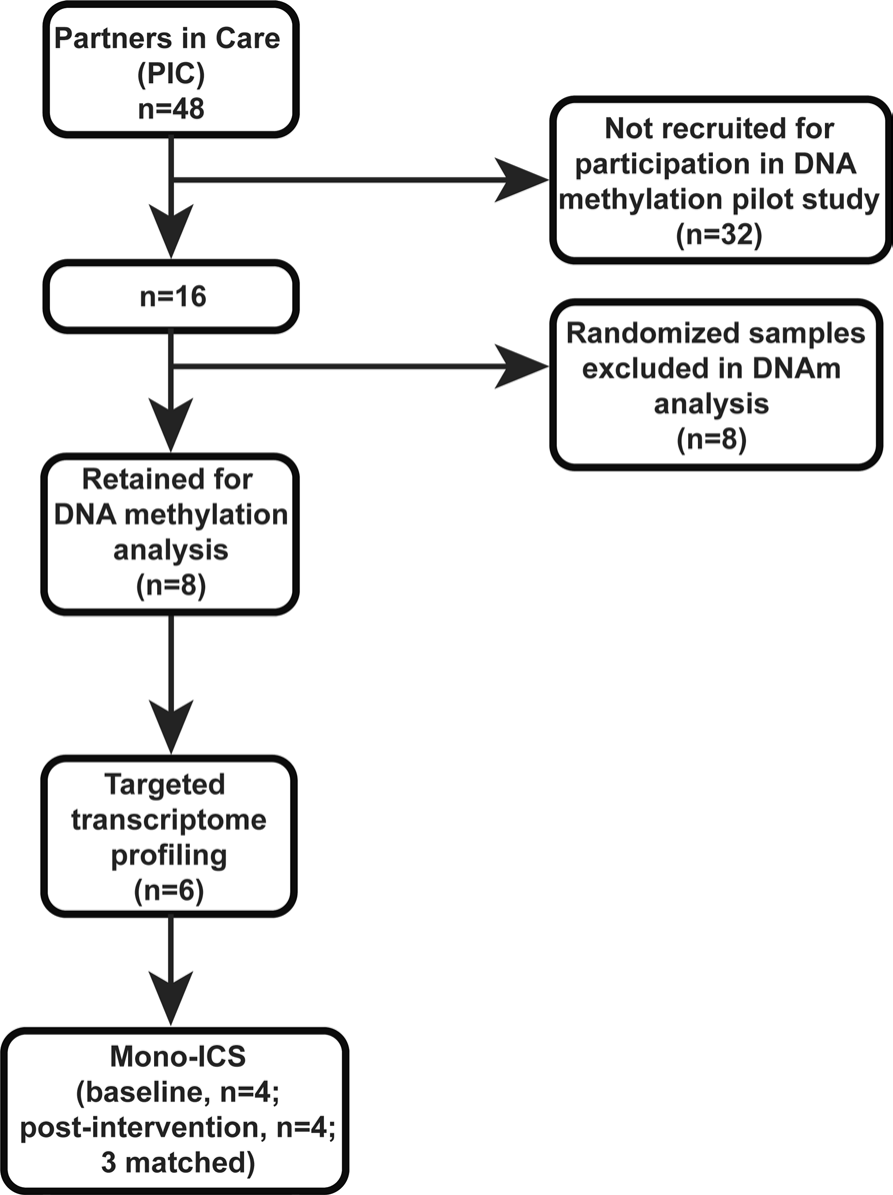
Flowchart of participant inclusion. Flowchart schematic represents the participants included in each analysis of the study

For this pilot project, participants were recruited from the parent PIC intervention study from the same community, Papakōlea, and were a part of the Kula no Nā Poʻe Hawaiʻi community organization. From a total of 48 participants in the parent intervention, we recruited 16 individuals for this pilot study according to the following inclusion criteria: (i) self-reported Native Hawaiian or other Pacific Islander ethnicity, (ii) 18 years of age or older, (iii) physician-diagnosed T2D, (iv) baseline HbA1c ≥ 7%, and exclusion criteria of (i) survival less than 6 months, (ii) moving off island or out of state during study period, (iii) pregnancy, and (iv) co-morbidities preventing participation. Of the 16 individuals, 8 individuals were randomly selected for complete DNA methylation analyses (Fig. 5); sample size consideration was based on a power analysis that allowed us to determine the lower sample size limit needed to maintain at least 80% power with a False Discovery Rate (FDR) controlled at 5% for differential methylation analysis. From this subset, we focused on transcriptomic and inflammatory phenotyping analyses. Written informed consent was obtained to participate in this supplemental pilot study. The informed consent was approved by two Institutional Review Boards: University of Hawai‘i and Papa Ola Lokahi Native Hawaiian Health Board.

### PBMC specimens, monocyte enrichment, and nucleic acid isolation

At the community site for the DM-SSP intervention, licensed phlebotomists drew 20 ml of anti-coagulated blood from each consenting participant at baseline and post-intervention. De-identified PBMCs were separated from whole blood via gradient centrifugation with Ficoll-Paque (Miltenyi Biotec, Bergisch Gladbach, Germany) and cryopreserved in liquid nitrogen immediately after separation. It is important to note that at the request of the Kula no Nā Poʻe Hawaiʻi, the community organization who recruited patients and facilitated the DM-SSP, no remaining blood or its biological components were stored beyond what was used for this study. Viably cryopreserved PBMCs from participants at both timepoints were first thawed in AIM-V Serum Free Media (Thermo Fisher Scientific, Inc., Waltham, MA, USA) supplemented with 1:50 DNase (Sigma-Aldrich, St. Louis, MO, USA), washed, media aspirated, and resuspended in wash buffer (PBS, 3% BSA, and 1 mM EDTA). Cells were counted for each sample using a Countess Cell Counter (Thermo Fisher Scientific) to determine viability and cell concentration (live cell counts). Approximately, 1.25 × 10^5^ aliquots were taken from each sample for flow cytometry-based cellular phenotyping analysis to determine cell type composition of PBMCs prior to enrichment of monocytes. PBMCs were then subjected to bead-based immunomagnetic targeted immune cell enrichment utilizing the Negative Selection Human Monocyte Enrichment Kit without CD16 Depletion (StemCell Technologies, Inc., Vancouver, BC, Canada) following manufacturer’s guidelines for the EasySep™ magnet (StemCell Technologies). To determine the efficiency of monocyte enrichment, negatively selected cells (monocytes) were counted and partitioned to 1.0 × 10^5^–1.25 × 10^5^ cells for flow cytometry analysis of enriched cells. The remainder of negatively selected cells were pelleted and resuspended in lysis buffer for subsequent purification of nucleic acids. Isolation of DNA and RNA was performed using the AllPrep® DNA/RNA Mini Kit (Qiagen, Hilden, Germany) according to manufacturer’s recommendations for purification of DNA and RNA from animal cells. Nucleic acid concentrations were quantified using the Qubit® 2.0 Fluorometer (Thermo Fisher Scientific) following the manufacturer’s protocol with the Qubit® dsDNA HS Assay Kit and Qubit® RNA BR Assay Kit (Thermo Fisher Scientific).

### Assessment of cell population purity

PBMCs and immunomagnetically isolated total monocytes were assessed for cellular heterogeneity by immunophenotyping of sample aliquots. Cells were immunophenotyped based on CD14 and CD16 expression using our established multi-parametric panel of antibodies that identify the HLA-DR^+^, live cell population that exclude T cells, B cells and NK cells (CD3^−^CD19^−^CD14^−^CD20^−^CD56^−^), and dead cells (yellow amine reactive dye [YARD^+^]). Aliquots were stained with YARD (Thermo Fisher Scientific) and then with mouse antihuman fluorochromes targeted for, anti-CD16 Brilliant Violet 421 (Clone 3G8), anti-CD3 V500 (Clone UCHT1), anti-CD14 Qdot®605 (Clone TüK4), anti-CD56 Pe-Cy7 (Clone B159), anti-CD19 PE-Cy7 (Clone SJ25C1), anti-CD20 Pe-Cy7 (Clone 2H7), and anti-HLA-DR APC-H7 (Clone G46-6) for identification of leukocyte subpopulation frequencies. Anti-CD16 was purchased from BioLegend, Inc., San Diego, CA, USA. Anti-CD3, anti-CD56, anti-CD20, anti-CD19, anti-HLA-DR were obtained from BD Biosciences, San Jose, CA, USA. Anti-mouse Ig/Negative Control (FBS) Compensation Particle Set (BD Biosciences) was used for compensation analysis of fluorescent signals emitted by each fluorochrome from the multi-colored cellular phenotyping panel employed. Anti-mouse Ig compensation beads were stained with each fluorochrome-conjugated antibody in separate wells. ArC Amine Reactive Compensation Bead Kit (Thermo Fisher Scientific) reactive bead/negative beads were used for compensation of YARD (Live/Dead stain) fluorescent signals. Stained cells from PBMCs, enriched monocytes, and compensation particles were analyzed using a 4-laser BD LSRFortessa flow cytometer (BD Biosciences). Data were analyzed using the FlowJo software (Tree Star, Inc., Ashland, OR, USA). The frequency (as %) of monocytes was determined by event count (specific event/total events) with debris exclusion.

### DNA methylation analysis

From a subset of participants (*n* = 8), DNA methylation analysis was performed on enriched monocytes at single-nucleotide resolution using the well-established Infinium® HumanMethylation450 BeadChip (450K) microarray (Illumina, San Diego, CA, USA). Briefly, 500 ng of DNA per sample were bisulfite-converted using the EZ DNA Methylation kit (Zymo Research, Irvine, CA, USA) according to the manufacturer’s instructions. Bisulfite-converted DNA (4 μl per sample) were assigned to a chip well of the 450 K, amplified, hybridized onto the array, and imaged with the iScan SQ instrument (Illumina) to obtain raw image intensities. Array IDAT raw intensity data were preprocessed in R statistical environment 3.1.2 using the *RnBeads* 0.99.18 pipeline analysis package [101] (Additional File 1: Fig. S1), and raw IDAT files can be found at GEO Accession: GSE197881. Raw data were quality-controlled using internal control probes to check for samples that could bias normalization and preprocessing was performed to remove missing probes, SNP-enriched probes, non-specific probes and low detection *P* value probes (detection *P* > 0.05). Methylation β-values ranging from 0 to 1 (corresponding to unmethylated to methylated signal intensity) for each sample were normalized using the subset quantile within-array normalization (SWAN) method within the *minfi* package in the *RnBeads* pipeline, a methodology we have previously used [40, 41]. A total of ~ 8,000 probes were removed after filtering. Differential methylation of specific cytosine-guanine dinucleotides (CpGs) was determined by a resampling-based empirical Bayesian Method permutation approach and those with a FDR of *P* < 0.05 were deemed significant [39]. This approach yielded 118,338 CpGs. Significantly differentially methylated CpGs (*P* < 0.05) were further filtered for absolute differences in methylation of ≥ 15% (δβ-value) between intervention timepoints. Gplot within the Bioconductor package was used to generate heatmaps of differential methylation between intervention timepoints. Unsupervised hierarchical clustering was performed using Manhattan distance, complete linkage method.

### Cell type-specific differential methylation validation of monocyte enrichment

To corroborate monocyte enrichment immunophenotyping data, we used DNA methylation data derived from participant-derived primary monocytes and compared them to methylation states of known PBMCs and sorted monocyte methylation states previously described [38], a technique our laboratory applies to validate monocyte enrichment methods [40, 41]. Briefly, 450K microarray data were downloaded from GEO Accession: GSE35069 [38]. First, cell type-specific methylation data from fluorescence-activated cell-sorted (FACS) monocytes (*n* = 6) and PBMCs (*n* = 6) were used to determine cell type-specific DNA methylation sites using the resampling-based empirical Bayes methods permutation approach as performed above but with an absolute difference in DNA methylation ≥ 30% between monocytes and PBMCs [39]. This stringent cutoff produced 5,124 CpGs, whose mean methylation states distinguished monocytes from PBMCs. Pearson’s correlation was used to determine the degree of similarity or difference between the methylation states at these locus-specific CpGs distinguishing PBMCs from monocytes and confirming our monocyte enrichment protocol.

### Transcriptome profiling by AmpliSeq

By utilizing targeted amplicon-based sequencing of the ~ 20,000 RefSeq genes, we surveyed differential gene expression of all protein-coding genes. From the subset of participants examined for DNA methylation analysis (*n* = 8), we selected 6 participants to investigate DEGs in monocytes between intervention timepoints. Whole-genome targeted transcriptomics by AmpliSeq™ (Thermo Fisher Scientific) was performed using the semiconductor-based sequencing on the Ion Torrent: Proton™ Sequencer (Thermo Fisher Scientific). Library preparation was first performed on a minimum of 10 ng of total RNA. Total RNA was reverse transcribed to a cDNA library using SuperScript™ VILO™ cDNA Synthesis Kit (Thermo Fisher Scientific) and amplified with an AmpliSeq™ PCR Mix (Thermo Fisher Scientific). Following amplification, barcoded adapters were ligated for multiplexing samples and amplicons purified. Using the Agilent 2100 Bioanalyzer™ (Agilent Technologies, Santa Clara, CA, USA), AmpliSeq libraries were quantified with the Agilent High Sensitivity DNA Kit (Agilent Technologies), diluted to ~ 100 pM, and pooled at this equimolar concentration. Pooled libraries were then amplified by emulsion PCR performed on the Ion Torrent OneTouch™ 2 System (Thermo Fisher Scientific) following manufacturer’s protocol. Templated libraries were subsequently loaded onto an Ion Torrent Ion PI™ Chip v3 (Thermo Fisher Scientific) and sequenced on the Ion Torrent: Proton™ System (Thermo Fisher Scientific) following manufacturer’s protocol. Raw reads were aligned using open-access software pipeline, Tuxedo (Oracle Corporation, Redwood Shores, CA, USA). Within Tuxedo, Bowtie was utilized to map and align reads to a reference genome [102]. Differential expression was performed on normalized expression values (Reads Per Million reads) of each amplicon with open-access software DESeq2 from RStudio’s Bioconductor package [103]. The FDR was calculated pre- and post-intervention and filtered for significance at *P* < 0.05. Gplot within the Bioconductor package was used to generate heatmaps of differentially expressed amplicons between pre-intervention and post-intervention. Unsupervised hierarchical clustering was performed using Manhattan distance, complete linkage method.

### Monocyte intracellular cytokine staining inflammatory phenotyping panel

Cryopreserved PBMCs were thawed and followed with a resting overnight period at 37 °C and 5% CO_2_ in a polypropylene plate, a protocol optimized by Jalbert et al. [104], to avoid monocyte differentiation in culture, and to preserve ex vivo surface expression of CD14 and CD16 [104]. The cells were stimulated with LPS (100 ng/ml) or media alone (unstimulated) for 6 h in the presence of brefeldin A (5 μg/ml) and monensin (5 μg/ml). Cells were then surface-stained with mouse antihuman fluorochromes for: anti-CD3 (V500), anti-CD14 (Qdot605), anti-CD16 (Alexa700), anti-CD56 (PE-Cy7), anti-CD19 (PE-Cy7), anti-CD20 (PECy7), anti-HLA-DR (APC-H7), and with Live/Dead fixable yellow dead cell stain (YARD). Cells were subsequently fixed, permeabilized with BD FACS Permeabilizing Buffer II (BD Biosciences) and stained with mouse antihuman conjugated antibodies: anti-IL-1β (PE), anti-IL-8 (FITC), anti-IL-6 (APC) and anti-TNF-α (PerCP-Cy5.5). Data were acquired on a custom 4-laser BD LSRFortessa (BD Biosciences), and all compensation and gating analyses were performed in FlowJo (TreeStar). Data were analyzed based on gating strategies described by Jalbert et al. [104]. For each individual, the degree of monocyte inflammatory response was calculated based on the percent of stimulated minus unstimulated cells producing a positive fluorescent signal normalized by total monocyte cell counts determined by flow cytometric technology.

### Gene ontology analysis

Gene ontology (GO) analysis was performed using the publicly available analysis program, Enrichr (https://amp.pharm.mssm.edu/Enrichr/) [51, 52], which utilizes gene-level or chromosomal position of CpGs of interest to determine the nearest gene(s) for each CpG and the functional relevance to specific molecular, cellular, or biological processes, and pathway analysis. Species assembly used was human: GRCH37 (UCSC hg19, Feb/2009). GO analysis included annotation of the DML and differentially expressed genes (DEGs) from pre- and post-intervention timepoints. Statistical significance of GO results was determined using Fisher’s exact test; significance at *P* < 0.05.

### Statistical analysis

Comparative analyses of clinical and immunological data between pre- and post-intervention were performed using parametric, paired *t* tests. Comparative analyses of differential DNA methylation between baseline and 3 months (FDR at *P* < 0.05, δβ-value ≥ 0.15) were performed using parametric, paired *t* tests. Chi-square test was performed for analysis of observed vs. expected CpG distribution. Mono-ICS was analyzed using parametric, unpaired student *t* tests; paired data from both timepoints for some participants were unavailable for paired statistical analyses. Pearson’s correlation was calculated to determine statistical significance for all associations tested. All tests were determined significant at a threshold of *P* < 0.05. Graphing and statistical analyses were performed using Prism 8, Version 8.4.3 (GraphPad, La Jolla, CA, USA) and R (version 1.4.1106).

## Supplementary Information

**Additional file 1: Fig. S1.** R-script for DNA methylation pre-processing.

**Additional file 2: Table S1.** Clinical comparison between participants included for DNAm analysis and remaining DM-SSP participants.

**Additional file 3: Table S2.** Clinical and demographic data for non-DM donors.

**Additional file 4: Table S3.** Gene ontology analysis of differentially methylated loci.

**Additional file 5: Table S4.** Gene ontology analysis of differentially expressed genes.

## Data Availability

The data and materials supporting the findings of this study can be accessed at the Gene Expression Omnibus (https://www.ncbi.nlm.nih.gov/geo/) under the GEO Accession: GSE197881. The authors confirm that the data supporting findings from this study can be accessed in the supplementary materials.
