# Supplementary material for: Shifts in the immunoepigenomic landscape of monocytes in response to a diabetes-specific social support intervention: a pilot study among Native Hawaiian adults with diabetes
Source: Clin Epigenetics. 2022 Jul 18;14:91. doi: 10.1186/s13148-022-01307-6 (PMC9295496; doi:10.1186/s13148-022-01307-6)
Supplement: Supplementary file 1 — Additional file 1: Fig. S1. R-script for DNA methylation pre-processing. [file 13148_2022_1307_MOESM1_ESM.docx]

**Comprehensive Analysis of DNA Methylation Data^1^**

(<https://www.bioconductor.org/packages/release/bioc/vignettes/RnBeads/inst/doc/RnBeads.pdf>)

# Load RnBeads Package, install.packages if necessary

if (!requireNamespace("BiocManager", quietly=TRUE))
+ install.packages("BiocManager")
BiocManager::install(c("RnBeads", "RnBeads.hg38"))

library(RnBeads)

# SET UP ANALYSIS ENVIRONMENT

#define the directory structure

#setwd(".")

setwd("File_Path_to_Directory")

# Directory where your data is located

data.dir <- file.path(getwd(), "data")

datasetDir <- file.path(data.dir, "dataset")

idat.dir <- file.path(datasetDir, "idat_file_folder")

sample.annotation <- file.path(datasetDir, "Sample_Annotation_Worksheet.csv")

## Directory where the output should be written to

analysis.dir <- file.path(getwd(), "results")

## Directory where the report files should be written to

## New report directory before every new analysis

report.dir <- file.path(analysis.dir, "Name_of_Report_Directory")

rnb.initialize.reports(report.dir)

logger.start(fname=NA)

## configure analysis parameters and reporting parameters for RnBeads analyses

rnb.options(

filtering.sex.chromosomes.removal = FALSE,

identifiers.column = "Sample_Name",

exploratory.correlation.qc = FALSE,

exploratory.intersample = FALSE,

exploratory.region.profiles = character(0),

exploratory.clustering ="top",

exploratory.clustering.top.sites = 100,

# region.types = c("promoters", "genes", "tiling"),

region.types = NULL,

differential.report.sites = FALSE,

differential.comparison.columns = NULL,

qc = TRUE,

qc.boxplots = TRUE,

qc.barplots = TRUE,

qc.negative.boxplot = TRUE,

preprocessing = TRUE,

normalization = NULL,

normalization.method = "swan,

filtering.snp = "3",

filtering.cross.reactive = FALSE,

filtering.greedycut = NULL,

filtering.greedycut.pvalue.threshold = 0.05,

filtering.missing.value.quantile = 1,

export.to.csv = TRUE

)

# Data Import

data.source <- c(idat.dir, sample.annotation)

## Load the dataset into an RnBeadSet object

result <- rnb.run.import(data.source=data.source, data.type="infinium.idat.dir",

+ dir.reports=report.dir)

rnb.set <- result$rnb.set

rnb.set <- rnb.execute.import(data.source=data.source, data,

+ data.type="infinium.idat.dir")

# Quality Control

## QC probes present within input data; start with IDAT files

#command (below) generates QC report (qc.html)

rnb.run.qc(rnb.set, report.dir)

## Command to generate boxplot and barplot w/ unique identifier

rnb.plot.control.boxplot(rnb.set, "BISULFITE CONVERSION I")

rnb.plot.control.barplot(rnb.set, "BISULFITE CONVERSION I.2")

## Command for negative control

rnb.plot.negative.boxplot(rnb.set)

# Preprocessing – Filtering and Normalization

rnb.set.unfiltered <- rnb.set

result <-rnb.run.preprocessing(rnb.set.unfiltered, dir.reports=report.dir)

rnb.set <- result$rnb.set

# Tracks and Tables

rnb.options(export.to.csv=TRUE)

rnb.run.tnt(rnb.set, report.dir)

#Beta-values exported as CSV across all ~450,000 preprocessed probes can be subjected to #permutation analysis for significantly differentially methylated CpGs

**Reference**

1 Assenov, Y. *et al.* Comprehensive analysis of DNA methylation data with RnBeads. *Nat Methods* **11**, 1138-1140, doi:10.1038/nmeth.3115 (2014).
