# Supplementary material for: Shifts in the immunoepigenomic landscape of monocytes in response to a diabetes-specific social support intervention: a pilot study among Native Hawaiian adults with diabetes
Source: Clin Epigenetics. 2022 Jul 18;14:91. doi: 10.1186/s13148-022-01307-6 (PMC9295496; doi:10.1186/s13148-022-01307-6)
Supplement: Supplementary file 2 — Additional file 2: Table S1. Clinical comparison between participants included for DNAm analysis and remaining DM-SSP participants. [file 13148_2022_1307_MOESM2_ESM.docx]

| **Supplementary Table I. Comparison of Clinical Characteristics Between Randomly Selected Participants and DM-SSP-Enrolled Participants** | **Randomly Selected (n=8)** | **DM-SSP-Enrolled (n=8)** | ***P*-Value** |  |
| --- | --- | --- | --- | --- |
|  |  |  |  |  |
| Hemoglobin A1c, mean (SD) | 8.9 (1.3) | 9.1 (2.0) | 0.78 |  |
| Weight, lbs, mean (SD) | 224.9 (37.0) | 237.0 (84.7) | 0.72 |  |
| Body Mass Index, kg/m^2, mean (SD) | 36.2 (5.2) | 36.6 (8.4) | 0.92 |  |
| Systolic Blood Pressure, mmHg, mean (SD) | 121.9 (10.2) | 129.1 (14.0) | 0.28 |  |
| Diastolic Blood Pressure, mmHg, mean (SD) | 76.2 (7.5) | 71.6 (9.7) | 0.33 |  |
| Lipids, mg/dL, mean (SD): |  |  |  |  |
| Total Cholesterol | 196.8 (49.0) | 169.8 (34.0) | 0.22 |  |
| High-Density Lipoprotein Cholesterol | 37.7 (7.0) | 33.8 (6.2) | 0.39 |  |
| Low-Density Lipoprotein Cholesterol | 85.3 (23.9) | 88.8 (36.4) | 0.89 |  |
| Triglycerides | 413.4 (258.0) | 286.3 (139.2) | 0.24 |  |
| Problem Areas in Diabetes Score, mean (SD) | 46.7 (20.7) | 42.2 (17.0) | 0.64 |  |
| Diabetes Care Profile, mean (SD) | 2.7 (0.8) | 3.3 (0.4) | 0.09 |  |
| Summary of Diabetes Self-Care Attitudes, mean (SD) | 14.3 (4.1) | 18.3 (5.3) | 0.11 |  |
