# Supplementary material for: Shifts in the immunoepigenomic landscape of monocytes in response to a diabetes-specific social support intervention: a pilot study among Native Hawaiian adults with diabetes
Source: Clin Epigenetics. 2022 Jul 18;14:91. doi: 10.1186/s13148-022-01307-6 (PMC9295496; doi:10.1186/s13148-022-01307-6)
Supplement: Supplementary file 3 — Additional file 3: Table S2. Clinical and demographic data for non-DM donors. [file 13148_2022_1307_MOESM3_ESM.docx]

| **Supplementary Table 2. Participant Characteristics of Non-Diabetic Donors (n=2)** | |
| --- | --- |
| Age, years, mean (SD) | 48.5 (7.1) |
| Gender (Male; n) | 2 |
| Currently Smoking (n) | 0 |
| Systolic Blood Pressure (mmHg) | 107.0 (2.7) |
| Total Cholesterol (mg/dL) | 203.5 (45.6) |
| HDL Cholesterol (mg/dL) | 66.5 (11.7) |
| HOMA-IR | 0.94 (0.3) |
