# Supplementary material for: Shifts in the immunoepigenomic landscape of monocytes in response to a diabetes-specific social support intervention: a pilot study among Native Hawaiian adults with diabetes
Source: Clin Epigenetics. 2022 Jul 18;14:91. doi: 10.1186/s13148-022-01307-6 (PMC9295496; doi:10.1186/s13148-022-01307-6)
Supplement: Supplementary file 4 — Additional file 4: Table S3. Gene ontology analysis of differentially methylated loci. [file 13148_2022_1307_MOESM4_ESM.docx]

| **Supplementary Table 3. DML GO Analysis** | | | | | | | | | | | |
| --- | --- | --- | --- | --- | --- | --- | --- | --- | --- | --- | --- |
| **NCI Nature Pathways** | | | | | | | | | | | |
| **Term** | | **GO *P*-value** | **Combined Score** | **Gene(s)** | **CpG Probe** | **Chromosome Position** | **Genomic Location** | **Baseline Mean β±SD** | **Post-Intervention Mean β±SD** | **Mean β Diff.** | ***P*-value** |
| Class I PI3K signaling events | 0.00004 | | 16.87 | *ADAP1* | cg02123534 | Chr7:965485-965486 | Body | 0.27±0.06 | 0.11±0.04 | -0.16 | 0.0003 |
|  | 0.00004 | | 16.87 | *ADAP1* | cg00904258 | Chr7:968194-968195 | Body | 0.50±0.07 | 0.34±0.10 | -0.16 | 0.0002 |
|  | 0.00004 | | 16.87 | *ADAP1* | cg21842920 | Chr7:968493-968494 | Body | 0.43±0.11 | 0.26±0.11 | -0.17 | 0.0002 |
|  | 0.00004 | | 16.87 | *ADAP1* | cg18332229 | Chr7:971130-971131 | Body | 0.54±0.08 | 0.37±0.11 | -0.16 | 0.0001 |
|  | 0.00004 | | 16.87 | *ADAP1* | cg04786889 | Chr7:990150-990151 | Body | 0.55±0.12 | 0.31±0.07 | -0.24 | 0.0003 |
|  | 0.00004 | | 16.87 | *BLK* | cg15742700 | Chr8:11350853-11350854 | TSS1500 | 0.50±0.08 | 0.30±0.06 | -0.20 | 0.001 |
|  | 0.00004 | | 16.87 | *BLNK* | cg08901339 | Chr10:98031261-98031262 | 5ʻUTR | 0.45±0.06 | 0.28±0.09 | -0.17 | 0.002 |
|  | 0.00004 | | 16.87 | *CYTH1* | cg07823562 | Chr17:76743501-76743502 | Body | 0.48±0.09 | 0.27±0.06 | -0.21 | 0.0004 |
|  | 0.00004 | | 16.87 | *FGR* | cg16922167 | Chr1:27961746-27961747 | TSS200 | 0.39±0.08 | 0.20±0.06 | -0.19 | 0.001 |
|  | 0.00004 | | 16.87 | *FGR* | cg13448978 | Chr1:27961796-27961797 | TSS200 | 0.26±0.09 | 0.08±0.04 | -0.18 | 0.001 |
|  | 0.00004 | | 16.87 | *PTEN* | cg02307823 | Chr10:89675901-89675902 | Body | 0.62±0.08 | 0.45±0.08 | -0.18 | 0.003 |
|  | 0.00004 | | 16.87 | *RAC1* | cg15381475 | Chr7:6436101-6436102 | Body | 0.22±0.06 | 0.07±0.03 | -0.15 | 0.001 |
|  | 0.00004 | | 16.87 | *SGK1* | cg08550353 | Chr6:134497627-134497628 | TSS1500 | 0.28±0.08 | 0.10±0.03 | -0.17 | 0.001 |
| EPHB forward signaling | 0.0003 | | 12.67 | *EFNB2* | cg25814649 | Chr13:107143980-107143981 | 3ʻUTR | 0.32±0.07 | 0.16±0.04 | -0.16 | 0.001 |
|  | 0.0003 | | 12.67 | *GRB7* | cg14398691 | Chr17:37895771-37895772 | 5ʻUTR | 0.48±0.04 | 0.32±0.04 | -0.16 | 0.00001 |
|  | 0.0003 | | 12.67 | *NCK1* | cg00382999 | Chr3:13664933-136649334 | Body | 0.42±0.07 | 0.26±0.09 | -0.17 | 0.002 |
|  | 0.0003 | | 12.67 | *PAK1* | cg24304210 | Chr11:77118761-77118762 | 5ʻUTR | 0.43±0.06 | 0.25±0.07 | -0.18 | 0.0002 |
|  | 0.0003 | | 12.67 | *RAC1* | cg15381475 | Chr7:6436101-6436102 | Body | 0.22±0.06 | 0.07±0.03 | -0.15 | 0.001 |
| Regulation of p38-alpha and p38-beta | 0.002 | | 28.09 | *BLK* | cg15742700 | Chr8:11350853-11350854 | TSS1500 | 0.50±0.08 | 0.30±0.06 | -0.20 | 0.001 |
|  | 0.002 | | 28.09 | *DUSP10* | cg19318653 | Chr1:221876616-221876617 | Body | 0.72±0.08 | 0.52±0.06 | -0.19 | 0.001 |
|  | 0.002 | | 28.09 | *FGR* | cg16922167 | Chr1:27961746-27961747 | TSS200 | 0.39±0.08 | 0.20±0.06 | -0.19 | 0.001 |
|  | 0.002 | | 28.09 | *FGR* | cg13448978 | Chr1:27961796-27961797 | TSS200 | 0.26±0.09 | 0.08±0.04 | -0.18 | 0.001 |
|  | 0.002 | | 28.09 | *PAK1* | cg24304210 | Chr11:77118761-77118762 | 5ʻUTR | 0.43±0.06 | 0.25±0.07 | -0.18 | 0.0002 |
|  | 0.002 | | 28.09 | *RAC1* | cg15381475 | Chr7:6436101-6436102 | Body | 0.22±0.06 | 0.07±0.03 | -0.15 | 0.001 |
|  | 0.002 | | 28.09 | *TRAF6* | cg01476222 | Chr11:36522143-36522144 | Body | 0.54±0.08 | 0.29±0.10 | -0.25 | 0.001 |
| **GO Biological Process** | | | | | | | | | | | |
| **Term** | **GO *P*-value** | | **Combined Score** | **Gene(s)** | **CpG Probe** | **Chromosome Position** | **Genomic Location** | **Baseline Mean β±SD** | **Post-Intervention Mean** β**±SD** | **Mean** β **Diff.** | ***P*-value** |
| regulation of cell migration | 0.00001 | | 15.26 | *ACVR1* | cg20453861 | Chr2:158687877-158687878 | 5ʻUTR | 0.84±0.04 | 0.67±0.08 | -0.18 | 0.00002 |
|  | 0.00001 | | 15.26 | *ADAM10* | cg02816525 | Chr15:59024033-59024034 | Body | 0.51±0.10 | 0.30±0.04 | -0.21 | 0.001 |
|  | 0.00001 | | 15.26 | *CCR1* | cg10499974 | Chr3:46244099-46244100 | 3ʻUTR | 0.45±0.06 | 0.29±0.10 | -0.16 | 0.002 |
|  | 0.00001 | | 15.26 | *CPNE3* | cg12162100 | Chr8:87529647-87529648 | 5ʻUTR | 0.61±0.07 | 0.44±0.06 | -0.17 | 0.000 |
|  | 0.00001 | | 15.26 | *DAG1* | cg09416188 | Chr3:49538552-49538553 | 5ʻUTR | 0.61±0.09 | 0.38±0.07 | -0.23 | 0.001 |
|  | 0.00001 | | 15.26 | *DUSP10* | cg19318653 | Chr1:221876616-221876617 | Body | 0.72±0.08 | 0.52±0.06 | -0.19 | 0.001 |
|  | 0.00001 | | 15.26 | *FER* | cg19730600 | Chr5:108088674-108088675 | 5ʻUTR | 0.42±0.04 | 0.25±0.05 | -0.17 | 0.0001 |
|  | 0.00001 | | 15.26 | *FGR* | cg16922167 | Chr1:27961746-27961747 | TSS200 | 0.39±0.08 | 0.20±0.06 | -0.19 | 0.001 |
|  | 0.00001 | | 15.26 | *FGR* | cg13448978 | Chr1:27961796-27961797 | TSS200 | 0.26±0.09 | 0.08±0.04 | -0.18 | 0.001 |
|  | 0.00001 | | 15.26 | *GCNT2* | cg23719713 | Chr6:10586167-10586168 | Body | 0.50±0.05 | 0.31±0.08 | -0.19 | 0.001 |
|  | 0.00001 | | 15.26 | *GNA12* | cg16569650 | Chr7:2773072-2773073 | Body | 0.53±0.05 | 0.37±0.10 | -0.16 | 0.004 |
|  | 0.00001 | | 15.26 | *GPER1* | cg21235678 | Chr7:1130967-1130968 | 5ʻUTR | 0.47±0.08 | 0.28±0.06 | -0.19 | 0.002 |
|  | 0.00001 | | 15.26 | *GRB7* | cg14398691 | Chr17:37895771-37895772 | 5ʻUTR | 0.48±0.04 | 0.32±0.04 | -0.16 | 0.00001 |
|  | 0.00001 | | 15.26 | *GTPBP4* | cg20744788 | Chr10:1043407-1043408 | Body | 0.83±0.08 | 0.66±0.09 | -0.17 | 0.001 |
|  | 0.00001 | | 15.26 | *KDR* | cg10858353 | Chr4:55945399-55945400 | 3ʻUTR | 0.69±0.08 | 0.53±0.10 | -0.16 | 0.01 |
|  | 0.00001 | | 15.26 | *LAMB1* | cg20438043 | Chr7:107601757-107601758 | Body | 0.77±0.11 | 0.61±0.05 | -0.16 | 0.001 |
|  | 0.00001 | | 15.26 | *LMO4* | cg20701183 | Chr1:87799914-87799915 | Body | 0.38±0.04 | 0.23±0.03 | -0.15 | 0.0001 |
|  | 0.00001 | | 15.26 | *PAK1* | cg24304210 | Chr11:77118761-77118762 | 5ʻUTR | 0.43±0.06 | 0.25±0.07 | -0.18 | 0.0002 |
|  | 0.00001 | | 15.26 | *PLXNB2* | cg14043316 | Chr22:50743420-50743421 | 5ʻUTR | 0.50±0.06 | 0.34±0.05 | -0.16 | 0.0002 |
|  | 0.00001 | | 15.26 | *PTEN* | cg02307823 | Chr10:89675901-89675902 | Body | 0.62±0.08 | 0.45±0.08 | -0.18 | 0.003 |
|  | 0.00001 | | 15.26 | *RAC1* | cg15381475 | Chr7:6436101-6436102 | Body | 0.22±0.06 | 0.07±0.03 | -0.15 | 0.001 |
|  | 0.00001 | | 15.26 | *SEMA4B* | cg24924577 | Chr15:90735148-90735149 | 5ʻUTR | 0.23±0.04 | 0.08±0.02 | -0.15 | 0.00005 |
|  | 0.00001 | | 15.26 | *SGK1* | cg08550353 | Chr6:134497627-134497628 | TSS1500 | 0.28±0.08 | 0.10±0.03 | -0.17 | 0.001 |
|  | 0.00001 | | 15.26 | *SORL1* | cg05921138 | Chr11:121440849-121440850 | Body | 0.54±0.07 | 0.37±0.06 | -0.17 | 0.0002 |
|  | 0.00001 | | 15.26 | *TGFBR1* | cg14426785 | Chr9:101876671-101876672 | Body | 0.37±0.05 | 0.20±0.03 | -0.17 | 0.0001 |
| regulation of protein kinase B signaling | 0.001 | | 8.69 | *DAG1* | cg09416188 | Chr3:49538552-49538553 | 5ʻUTR | 0.61±0.09 | 0.38±0.07 | -0.23 | 0.001 |
|  | 0.001 | | 8.69 | *FGF17* | cg03025830 | Chr8:21905599-21305600 | Body | 0.53±0.07 | 0.37±0.14 | -0.15 | 0.02 |
|  | 0.001 | | 8.69 | *GCNT2* | cg23719713 | Chr6:10586167-10586168 | Body | 0.50±0.05 | 0.31±0.08 | -0.19 | 0.001 |
|  | 0.001 | | 8.69 | *GPER1* | cg21235678 | Chr7:1130967-1130968 | 5ʻUTR | 0.47±0.08 | 0.28±0.06 | -0.19 | 0.002 |
|  | 0.001 | | 8.69 | *INPP5K* | cg07185664 | Chr17:1412950-1412951 | Body | 0.70±0.04 | 0.54±0.09 | -0.17 | 0.002 |
|  | 0.001 | | 8.69 | *ITGB1* | cg20545410 | Chr10:33232548-33232549 | 5ʻUTR | 0.33±0.06 | 0.16±0.04 | -0.17 | 0.0002 |
|  | 0.001 | | 8.69 | *PHLPP1* | cg13381110 | Chr18:60646614-60646615 | Body | 0.59±0.06 | 0.39±0.12 | -0.21 | 0.0005 |
|  | 0.001 | | 8.69 | *PTEN* | cg02307823 | Chr10:89675901-89675902 | Body | 0.62±0.08 | 0.45±0.08 | -0.18 | 0.003 |
|  | 0.001 | | 8.69 | *TCF7L2* | cg05923857 | Chr10:114911615-11411616 | Body | 0.45±0.07 | 0.22±0.09 | -0.23 | 0.0003 |
|  | 0.001 | | 8.69 | *TCF7L2* | cg24788483 | Chr10:114911652-114911653 | Body | 0.47±0.07 | 0.27±0.05 | -0.20 | 0.00003 |
|  | 0.001 | | 8.69 | *TGFBR1* | cg14426785 | Chr9:101876671-101876672 | Body | 0.37±0.05 | 0.20±0.03 | -0.17 | 0.0001 |
| negative regulation of systemic arterial blood pressure | 0.003 | | 21.04 | *ADRB2* | cg08370787 | Chr5:148208041-148208042 | 1st Exon | 0.70±0.08 | 0.51±0.04 | -0.18 | 0.0001 |
|  | 0.003 | | 21.04 | *PRCP* | cg19733740 | Chr11:82609924-82609925 | Body | 0.78±0.04 | 0.63±0.02 | -0.15 | 0.00001 |
| cellular response to low-density lipoprotein particle stimulus | 0.003 | | 14.26 | *CD36* | cg10207609 | Chr7:80267619-80267620 | TSS1500 | 0.54±0.07 | 0.31±0.10 | -0.23 | 0.001 |
|  | 0.003 | | 14.26 | *FCER1G* | cg20806175 | Chr1:161186839-161186840 | Body | 0.27±0.06 | 0.12±0.05 | -0.15 | 0.001 |
|  | 0.003 | | 14.26 | *ITGB1* | cg20545410 | Chr10:33232548-33232549 | 5ʻUTR | 0.33±0.06 | 0.16±0.04 | -0.17 | 0.0002 |
|  | 0.003 | | 14.26 | *PPARG* | cg18637222 | Chr3:12435731-12435732 | Body | 0.59±0.08 | 0.43±0.10 | -0.16 | 0.001 |
| Fc-gamma receptor signaling pathway | 0.003 | | 11.98 | *CYFIP2* | cg10578938 | Chr5:156695410-156695411 | 5ʻUTR | 0.49±0.04 | 0.66±0.06 | 0.17 | 0.0002 |
|  | 0.003 | | 11.98 | *ELMO1* | cg14431020 | Chr7:37256235-37256236 | Body | 0.67±0.10 | 0.47±0.03 | -0.20 | 0.001 |
|  | 0.003 | | 11.98 | *FCER1G* | cg20806175 | Chr1:161186839-161186840 | Body | 0.27±0.06 | 0.12±0.05 | -0.15 | 0.001 |
|  | 0.003 | | 11.98 | *FGR* | cg16922167 | Chr1:27961746-27961747 | TSS200 | 0.39±0.08 | 0.20±0.06 | -0.19 | 0.001 |
|  | 0.003 | | 11.98 | *FGR* | cg13448978 | Chr1:27961796-27961797 | TSS200 | 0.26±0.09 | 0.08±0.04 | -0.18 | 0.001 |
|  | 0.003 | | 15.26 | *NCK1* | cg00382999 | Chr3:13664933-136649334 | Body | 0.42±0.07 | 0.26±0.09 | -0.17 | 0.002 |
|  | 0.003 | | 11.98 | *PAK1* | cg24304210 | Chr11:77118761-77118762 | 5ʻUTR | 0.43±0.06 | 0.25±0.07 | -0.18 | 0.0002 |
|  | 0.003 | | 11.98 | *PRKCE* | cg02073763 | Chr2:46064034-46064035 | Body | 0.42±0.06 | 0.25±0.07 | -0.17 | 0.001 |
|  | 0.003 | | 11.98 | *RAC1* | cg15381475 | Chr7:6436101-6436102 | Body | 0.22±0.06 | 0.07±0.03 | -0.15 | 0.001 |
|  | 0.003 | | 11.98 | *WAS* | cg11654838 | ChrX:48541415-48541416 | TSS1500 | 0.70±0.08 | 0.54±0.20 | -0.16 | 0.029 |
| inositol phosphate dephosphorylation | 0.004 | | 15.46 | *INPP5A* | cg06760238 | Chr10:134400036-134400037 | Body | 0.29±0.09 | 0.09±0.03 | -0.20 | 0.001 |
|  | 0.004 | | 15.46 | *INPP5A* | cg12608975 | Chr10:134404873-134404874 | Body | 0.78±0.07 | 0.57±0.04 | -0.21 | 0.00002 |
|  | 0.004 | | 15.46 | *INPP5K* | cg07185664 | Chr17:1412950-1412951 | Body | 0.70±0.04 | 0.54±0.09 | -0.17 | 0.002 |
|  | 0.004 | | 15.46 | *PTEN* | cg02307823 | Chr10:89675901-89675902 | Body | 0.62±0.08 | 0.45±0.08 | -0.18 | 0.003 |
| glycerophospholipid biosynthetic process | 0.007 | | 9.89 | *CPNE3* | cg12162100 | Chr8:87529647-87529648 | 5ʻUTR | 0.61±0.07 | 0.44±0.06 | -0.17 | 0.0004 |
|  | 0.007 | | 9.89 | *ETNK1* | cg10456459 | Chr12:22843015-22843016 | 3ʻUTR | 0.55±0.08 | 0.35±0.11 | -0.19 | 0.001 |
|  | 0.007 | | 9.89 | *FAR1* | cg16636767 | Chr11:13694647-13694648 | 5ʻUTR | 0.70±0.07 | 0.39±0.14 | -0.30 | 0.001 |
|  | 0.007 | | 9.89 | *GPAM* | cg25290227 | Chr10:113944114-113944115 | TSS1500 | 0.72±0.09 | 0.56±0.07 | -0.15 | 0.002 |
|  | 0.007 | | 9.89 | *INPP4A* | cg18267330 | Chr2:99095277-99095278 | 5ʻUTR | 0.81±0.11 | 0.65±0.09 | -0.16 | 0.004 |
|  | 0.007 | | 9.89 | *INPP5D* | cg14012546 | Chr2:233981788-233981789 | Body | 0.50±0.05 | 0.34±0.07 | -0.16 | 0.001 |
|  | 0.007 | | 9.89 | *INPP5D* | cg22666015 | Chr2:233981885-233981886 | Body | 0.41±0.05 | 0.23±0.09 | -0.18 | 0.002 |
|  | 0.007 | | 9.89 | *INPP5K* | cg07185664 | Chr17:1412950-1412951 | Body | 0.70±0.04 | 0.54±0.09 | -0.17 | 0.002 |
|  | 0.007 | | 9.89 | *LPCAT1* | cg22185977 | Chr5:1518133-1518134 | Body | 0.42±0.05 | 0.25±0.08 | -0.17 | 0.001 |
|  | 0.007 | | 9.89 | *PLD1* | cg22466012 | Chr3:171466200-171466201 | 5ʻUTR | 0.52±0.13 | 0.33±0.05 | -0.19 | 0.01 |
|  | 0.007 | | 9.89 | *PTEN* | cg02307823 | Chr10:89675901-89675902 | Body | 0.62±0.08 | 0.45±0.08 | -0.18 | 0.003 |
|  | 0.007 | | 9.89 | *SLC27A1* | cg25517015 | Chr19:17584020-17584021 | Body | 0.59±0.07 | 0.35±0.11 | -0.25 | 0.001 |
| cellular response to cytokine stimulus | 0.009 | | 5.38 | *BCL2* | cg23756272 | Chr18:60904418-60904419 | Body | 0.39±0.05 | 0.23±0.04 | -0.16 | 0.0001 |
|  | 0.009 | | 5.38 | *CCR1* | cg10499974 | Chr3:46244099-46244100 | 3ʻUTR | 0.45±0.06 | 0.29±0.10 | -0.16 | 0.002 |
|  | 0.009 | | 5.38 | *FCER2* | cg12387247 | Chr19:7766974-7766975 | 1st Exon | 0.43±0.05 | 0.27±0.07 | -0.15 | 0.0004 |
|  | 0.009 | | 5.38 | *FER* | cg19730600 | Chr5:108088674-108088675 | 5ʻUTR | 0.42±0.04 | 0.25±0.05 | -0.17 | 0.0001 |
|  | 0.009 | | 5.38 | *FLT3* | cg20227511 | Chr13:28670602-28670603 | Body | 0.63±0.08 | 0.42±0.10 | -0.21 | 0.001 |
|  | 0.009 | | 5.38 | *GP1BA* | cg03451731 | Chr17:4837077-4837078 | Body | 0.80±0.10 | 0.63±0.07 | -0.17 | 0.001 |
|  | 0.009 | | 5.38 | *GPER1* | cg21235678 | Chr7:1130967-1130968 | 5ʻUTR | 0.47±0.08 | 0.28±0.06 | -0.19 | 0.002 |
|  | 0.009 | | 5.38 | *IFNGR2* | cg17356733 | Chr21:34774627-34774628 | TSS1500 | 0.56±0.06 | 0.35±0.11 | -0.21 | 0.0005 |
|  | 0.009 | | 5.38 | *IL10* | cg17744604 | Chr1:206946166-206946167 | TSS1500 | 0.38±0.06 | 0.22±0.09 | -0.15 | 0.001 |
|  | 0.009 | | 5.38 | *IL1A* | cg00839584 | Chr2:113542091-113542092 | 5ʻUTR | 0.45±0.06 | 0.28±0.08 | -0.17 | 0.001 |
|  | 0.009 | | 5.38 | *IL1A* | cg27606396 | Chr2:113542117-113542118 | 5ʻUTR | 0.54±0.08 | 0.39±0.08 | -0.16 | 0.002 |
|  | 0.009 | | 5.38 | *IL31RA* | cg07363330 | Chr5:55148493-55148494 | Body | 0.44±0.07 | 0.28±0.06 | -0.16 | 0.00004 |
|  | 0.009 | | 5.38 | *INPP5D* | cg14012546 | Chr2:233981788-233981789 | Body | 0.50±0.05 | 0.34±0.07 | -0.16 | 0.001 |
|  | 0.009 | | 5.38 | *INPP5D* | cg22666015 | Chr2:233981885-233981886 | Body | 0.41±0.05 | 0.23±0.09 | -0.18 | 0.002 |
|  | 0.009 | | 5.38 | *INPP5K* | cg07185664 | Chr17:1412950-1412951 | Body | 0.70±0.04 | 0.54±0.09 | -0.17 | 0.002 |
|  | 0.009 | | 5.38 | *ITGB1* | cg20545410 | Chr10:33232548-33232549 | 5ʻUTR | 0.33±0.06 | 0.16±0.04 | -0.17 | 0.0002 |
|  | 0.009 | | 5.38 | *NPR2* | cg24194775 | Chr9:35791475-35791476 | TSS1500 | 0.45±0.14 | 0.26±0.15 | -0.18 | 0.05 |
|  | 0.009 | | 5.38 | *PTPN12* | cg04776231 | Chr7:77168113-77168114 | 5ʻUTR | 0.28±0.12 | 0.10±0.03 | -0.19 | 0.008 |
|  | 0.009 | | 5.38 | *RBMX* | cg02936290 | ChrX:135962199-135962200 | Body | 0.30±0.08 | 0.48±0.09 | 0.17 | 0.0002 |
|  | 0.009 | | 5.38 | *SMAD3* | cg22528123 | Chr15:67417964-67417965 | TSS200 | 0.81±0.06 | 0.62±0.06 | -0.19 | 0.0004 |
|  | 0.009 | | 5.38 | *USP10* | cg05298510 | Chr16:84776329-84776330 | Body | 0.61±0.07 | 0.41±0.10 | -0.20 | 0.001 |
| regulation of phosphatidylinositol 3-kinase signaling | 0.01 | | 5.83 | *FGR* | cg16922167 | Chr1:27961746-27961747 | TSS200 | 0.39±0.08 | 0.20±0.06 | -0.19 | 0.0006 |
|  | 0.01 | | 5.83 | *FGR* | cg13448978 | Chr1:27961796-27961797 | TSS200 | 0.26±0.09 | 0.08±0.04 | -0.18 | 0.0014 |
|  | 0.01 | | 5.83 | *FLT3* | cg20227511 | Chr13:28670602-28670603 | Body | 0.63±0.08 | 0.42±0.10 | -0.21 | 0.001 |
|  | 0.01 | | 5.83 | *GPER1* | cg21235678 | Chr7:1130967-1130968 | 5ʻUTR | 0.47±0.08 | 0.28±0.06 | -0.19 | 0.002 |
|  | 0.01 | | 5.83 | *KDR* | cg10858353 | Chr4:55945399-55945400 | 3ʻUTR | 0.69±0.08 | 0.53±0.10 | -0.16 | 0.01 |
|  | 0.01 | | 5.83 | *NLRC3* | cg16429499 | Chr16:3597151-3597152 | Body | 0.48±0.08 | 0.22±0.10 | -0.26 | 0.0002 |
|  | 0.01 | | 5.83 | *PTEN* | cg02307823 | Chr10:89675901-89675902 | Body | 0.62±0.08 | 0.45±0.08 | -0.18 | 0.003 |
| regulation of NLRP3 inflammasome complex assembly | 0.01 | | 11.73 | *CD36* | cg10207609 | Chr7:80267619-80267620 | TSS1500 | 0.54±0.07 | 0.31±0.10 | -0.23 | 0.001 |
|  | 0.01 | | 11.73 | *MEFV* | cg00599219 | Chr16:3307037-3307038 | TSS1500 | 0.44±0.05 | 0.27±0.05 | -0.17 | 0.0001 |
|  | 0.01 | | 11.73 | *NLRC3* | cg16429499 | Chr16:3597151-3597152 | Body | 0.48±0.08 | 0.22±0.10 | -0.26 | 0.0002 |
| regulation of cholesterol storage | 0.02 | | 10.64 | *ABCG1* | cg05046272 | Chr21:43619510-43619511 | TSS1500 | 0.73±0.08 | 0.54±0.05 | -0.18 | 0.0001 |
|  | 0.02 | | 10.64 | *PPARG* | cg18637222 | Chr3:12435731-12435732 | Body | 0.59±0.08 | 0.43±0.10 | -0.16 | 0.001 |
| negative regulation of interleukin-12 production | 0.02 | | 8.84 | *IL10* | cg17744604 | Chr1:206946166-206946167 | TSS1500 | 0.38±0.06 | 0.22±0.09 | -0.15 | 0.001 |
|  | 0.02 | | 8.84 | *MEFV* | cg00599219 | Chr16:3307037-3307038 | TSS1500 | 0.44±0.05 | 0.27±0.05 | -0.17 | 0.0001 |
|  | 0.02 | | 8.84 | *TLR8* | cg13153942 | ChrX:12924247-12924248 | TSS1500 | 0.75±0.11 | 0.59±0.05 | -0.16 | 0.002 |
|  | 0.02 | | 8.84 | *TLR8* | cg20981403 | ChrX:12940308-12940309 | 3ʻUTR | 0.76±0.13 | 0.55±0.06 | -0.21 | 0.001 |
| regulation of JUN kinase activity | 0.02 | | 6.74 | *DUSP10* | cg19318653 | Chr1:221876616-221876617 | Body | 0.72±0.08 | 0.52±0.06 | -0.19 | 0.001 |
|  | 0.02 | | 6.74 | *PAK1* | cg24304210 | Chr11:77118761-77118762 | 5ʻUTR | 0.43±0.06 | 0.25±0.07 | -0.18 | 0.0002 |
|  | 0.02 | | 6.74 | *TAOK3* | cg16301036 | Chr12:118808298-118808299 | 5ʻUTR | 0.59±0.07 | 0.43±0.13 | -0.16 | 0.001 |
|  | 0.02 | | 6.74 | *TRAF6* | cg01476222 | Chr11:36522143-36522144 | Body | 0.54±0.08 | 0.29±0.10 | -0.25 | 0.001 |
| regulation of JNK cascade | 0.02 | | 6.25 | *DUSP10* | cg19318653 | Chr1:221876616-221876617 | Body | 0.72±0.08 | 0.52±0.06 | -0.19 | 0.001 |
|  | 0.02 | | 6.25 | *IL1A* | cg00839584 | Chr2:113542091-113542092 | 5ʻUTR | 0.45±0.06 | 0.28±0.08 | -0.17 | 0.001 |
|  | 0.02 | | 6.25 | *IL1A* | cg27606396 | Chr2:113542117-113542118 | 5ʻUTR | 0.54±0.08 | 0.39±0.08 | -0.16 | 0.002 |
|  | 0.02 | | 6.25 | *PHLPP1* | cg13381110 | Chr18:60646614-60646615 | Body | 0.59±0.06 | 0.39±0.12 | -0.21 | 0.0005 |
|  | 0.02 | | 6.25 | *RAP2A* | cg03608515 | Chr13:98112404-98112405 | Body | 0.61±0.07 | 0.45±0.05 | -0.15 | 0.001 |
|  | 0.02 | | 6.25 | *TAOK3* | cg16301036 | Chr12:118808298-118808299 | 5ʻUTR | 0.59±0.07 | 0.43±0.13 | -0.16 | 0.001 |
| negative regulation of chemokine production | 0.02 | | 8.96 | *IL10* | cg17744604 | Chr1:206946166-206946167 | TSS1500 | 0.38±0.06 | 0.22±0.09 | -0.15 | 0.001 |
|  | 0.02 | | 8.96 | *MEFV* | cg00599219 | Chr16:3307037-3307038 | TSS1500 | 0.44±0.05 | 0.27±0.05 | -0.17 | 0.0001 |
| regulation of interleukin-1 beta production | 0.02 | | 8.41 | *AIM2* | cg27296413 | Chr1:159037843-159037844 | Body | 0.36±0.07 | 0.20±0.05 | -0.16 | 0.002 |
|  | 0.02 | | 8.41 | *FOXP1* | cg18067134 | Chr3:71084634-71084635 | Body | 0.56±0.09 | 0.37±0.10 | -0.19 | 0.003 |
|  | 0.02 | | 8.41 | *FOXP1* | cg02520804 | Chr3:71629054-71629055 | 5ʻUTR | 0.55±0.11 | 0.36±0.13 | -0.20 | 0.003 |
|  | 0.02 | | 8.41 | *MEFV* | cg00599219 | Chr16:3307037-3307038 | TSS1500 | 0.44±0.05 | 0.27±0.05 | -0.17 | 0.0001 |
| negative regulation of response to external stimulus | 0.02 | | 4.32 | *GPER1* | cg21235678 | Chr7:1130967-1130968 | 5ʻUTR | 0.47±0.08 | 0.28±0.06 | -0.19 | 0.002 |
|  | 0.02 | | 4.32 | *IL10* | cg17744604 | Chr1:206946166-206946167 | TSS1500 | 0.38±0.06 | 0.22±0.09 | -0.15 | 0.001 |
|  | 0.02 | | 4.32 | *MEFV* | cg00599219 | Chr16:3307037-3307038 | TSS1500 | 0.44±0.05 | 0.27±0.05 | -0.17 | 0.0001 |
|  | 0.02 | | 4.32 | *NLRC3* | cg16429499 | Chr16:3597151-3597152 | Body | 0.48±0.08 | 0.22±0.10 | -0.26 | 0.0002 |
|  | 0.02 | | 4.32 | *PPARG* | cg18637222 | Chr3:12435731-12435732 | Body | 0.59±0.08 | 0.43±0.10 | -0.16 | 0.001 |
|  | 0.02 | | 4.32 | *PTEN* | cg02307823 | Chr10:89675901-89675902 | Body | 0.62±0.08 | 0.45±0.08 | -0.18 | 0.003 |
|  | 0.02 | | 4.32 | *TRIB1* | cg02160684 | Chr8:126448033-126448034 | Body | 0.60±0.08 | 0.41±0.07 | -0.19 | 0.001 |
| Ras protein signal transduction | 0.02 | | 4.68 | *ELMO1* | cg14431020 | Chr7:37256235-37256236 | Body | 0.67±0.10 | 0.47±0.03 | -0.20 | 0.001 |
|  | 0.02 | | 4.68 | *GNA12* | cg16569650 | Chr7:2773072-2773073 | Body | 0.53±0.05 | 0.37±0.10 | -0.16 | 0.004 |
|  | 0.02 | | 4.68 | *KSR1* | cg26877678 | Chr17:25856540-25856541 | 5ʻUTR | 0.49±0.06 | 0.33±0.09 | -0.16 | 0.001 |
|  | 0.02 | | 4.68 | *PAK1* | cg24304210 | Chr11:77118761-77118762 | 5ʻUTR | 0.43±0.06 | 0.25±0.07 | -0.18 | 0.0002 |
|  | 0.02 | | 4.68 | *PLD1* | cg22466012 | Chr3:171466200-171466201 | 5ʻUTR | 0.52±0.13 | 0.33±0.05 | -0.19 | 0.009 |
|  | 0.02 | | 4.68 | *RAB40B* | cg22516137 | Chr17:80657589-80657590 | TSS1500 | 0.78±0.13 | 0.61±0.06 | -0.17 | 0.009 |
|  | 0.02 | | 4.68 | *RAC1* | cg15381475 | Chr7:6436101-6436102 | Body | 0.22±0.06 | 0.07±0.03 | -0.15 | 0.001 |
|  | 0.02 | | 4.68 | *RAP2A* | cg03608515 | Chr13:98112404-98112405 | Body | 0.61±0.07 | 0.45±0.05 | -0.15 | 0.001 |
|  | 0.02 | | 4.68 | *RAPGEF3* | cg14854517 | Chr12:48152428-48152429 | 1st Exon | 0.27±0.10 | 0.11±0.02 | -0.16 | 0.002 |
|  | 0.02 | | 4.68 | *RGL2* | cg12118504 | Chr6:33265393-33265394 | Body | 0.36±0.07 | 0.21±0.05 | -0.15 | 0.0001 |
| fatty acid transport | 0.03 | | 8.30 | *PPARG* | cg18637222 | Chr3:12435731-12435732 | Body | 0.59±0.08 | 0.43±0.10 | -0.16 | 0.001 |
|  | 0.03 | | 8.30 | *SLC27A1* | cg25517015 | Chr19:17584020-17584021 | Body | 0.59±0.07 | 0.35±0.11 | -0.25 | 0.001 |
|  | 0.03 | | 8.30 | *SLCO3A1* | cg21594043 | Chr15:92491578-92491579 | Body | 0.35±0.03 | 0.20±0.07 | -0.15 | 0.0003 |
| long-chain fatty acid transport | 0.03 | | 6.47 | *CD36* | cg10207609 | Chr7:80267619-80267620 | TSS1500 | 0.54±0.07 | 0.31±0.10 | -0.23 | 0.001 |
|  | 0.03 | | 6.47 | *PPARG* | cg18637222 | Chr3:12435731-12435732 | Body | 0.59±0.08 | 0.43±0.10 | -0.16 | 0.001 |
|  | 0.03 | | 6.47 | *SLC27A1* | cg25517015 | Chr19:17584020-17584021 | Body | 0.59±0.07 | 0.35±0.11 | -0.25 | 0.001 |
| regulation of p38MAPK cascade | 0.03 | | 5.84 | *DUSP10* | cg19318653 | Chr1:221876616-221876617 | Body | 0.72±0.08 | 0.52±0.06 | -0.19 | 0.001 |
|  | 0.03 | | 5.84 | *PHLPP1* | cg13381110 | Chr18:60646614-60646615 | Body | 0.59±0.06 | 0.39±0.12 | -0.21 | 0.0005 |
| ERBB2 signaling pathway | 0.03 | | 5.19 | *CPNE3* | cg12162100 | Chr8:87529647-87529648 | 5ʻUTR | 0.61±0.07 | 0.44±0.06 | -0.17 | 0.0004 |
|  | 0.03 | | 5.19 | *GRB7* | cg14398691 | Chr17:37895771-37895772 | 5ʻUTR | 0.48±0.04 | 0.32±0.04 | -0.16 | 0.00001 |
|  | 0.03 | | 5.19 | *PTPN12* | cg04776231 | Chr7:77168113-77168114 | 5ʻUTR | 0.28±0.12 | 0.10±0.03 | -0.19 | 0.01 |
| regulation of interleukin-12 secretion | 0.04 | | 8.35 | *FOXP1* | cg18067134 | Chr3:71084634-71084635 | Body | 0.56±0.09 | 0.37±0.10 | -0.19 | 0.003 |
|  | 0.04 | | 8.35 | *FOXP1* | cg02520804 | Chr3:71629054-71629055 | 5ʻUTR | 0.55±0.11 | 0.36±0.13 | -0.20 | 0.003 |
|  | 0.04 | | 8.35 | *TLR8* | cg13153942 | ChrX:12924247-12924248 | TSS1500 | 0.75±0.11 | 0.59±0.05 | -0.16 | 0.002 |
|  | 0.04 | | 8.35 | *TLR8* | cg20981403 | ChrX:12940308-12940309 | 3ʻUTR | 0.76±0.13 | 0.55±0.06 | -0.21 | 0.001 |
| acute inflammatory response | 0.04 | | 5.04 | *CD6* | cg01877352 | Chr11:60775233-60775234 | Body | 0.28±0.06 | 0.11±0.03 | -0.17 | 0.0002 |
| regulation of interleukin-23 production | 0.05 | | 8.47 | *RAC1* | cg15381475 | Chr7:6436101-6436102 | Body | 0.22±0.06 | 0.07±0.03 | -0.15 | 0.001 |
| receptor metabolic process | 0.05 | | 4.30 | *CD36* | cg10207609 | Chr7:80267619-80267620 | TSS1500 | 0.54±0.07 | 0.31±0.10 | -0.23 | 0.001 |
|  | 0.05 | | 4.30 | *FCER1G* | cg20806175 | Chr1:161186839-161186840 | Body | 0.27±0.06 | 0.12±0.05 | -0.15 | 0.001 |
|  | 0.05 | | 4.30 | *IL10* | cg17744604 | Chr1:206946166-206946167 | TSS1500 | 0.38±0.06 | 0.22±0.09 | -0.15 | 0.001 |
|  | 0.05 | | 4.30 | *ITGB1* | cg20545410 | Chr10:33232548-33232549 | 5ʻUTR | 0.33±0.06 | 0.16±0.04 | -0.17 | 0.0002 |
|  | 0.05 | | 4.30 | *LMTK2* | cg10412943 | Chr7:97739696-97739697 | Body | 0.85±0.04 | 0.70±0.07 | -0.16 | 0.0001 |
| **dbGaP GO Cardiometabolic Diseases** | | | | | | | | | | | |
| **Term** | **GO *P*-value** | | **Combined Score** | **Gene(s)** | **CpG Probe** | **Chromosome Position** | **Genomic Location** | **Baseline Mean β±SD** | **Post-Intervention Mean β±SD** | **Mean β Diff.** | ***P*-value** |
| hypertension | 0.000005 | | 54.57 | *CACNB2* | cg04764839 | Chr10:18708076-1870877 | Body | 0.61±0.07 | 0.31±0.07 | -0.30 | 0.000139 |
|  | 0.000005 | | 54.57 | *CUBN* | cg13223209 | Chr10:16986391-16986392 | Body | 0.53±0.07 | 0.36±0.10 | -0.16 | 0.001 |
|  | 0.000005 | | 54.57 | *CD36* | cg10207609 | Chr7:80267619-80267620 | TSS1500 | 0.54±0.07 | 0.31±0.10 | -0.23 | 0.001 |
|  | 0.000005 | | 54.57 | *ETV6* | cg06225767 | Chr12:11912232-11912233 | Body | 0.51±0.06 | 0.26±0.07 | -0.25 | 0.0002 |
|  | 0.000005 | | 54.57 | *ETV6* | cg14693090 | Chr12:11899281-11899282 | Body | 0.30±0.06 | 0.14±0.05 | -0.16 | 0.0003 |
|  | 0.000005 | | 54.57 | *FES* | cg26405020 | Chr15:91427363-91427364 | TSS1500 | 0.29±0.05 | 0.13±0.05 | -0.16 | 0.001 |
|  | 0.000005 | | 54.57 | *LPP* | cg15120525 | Chr3:188108233-188108234 | 5'UTR | 0.58±0.08 | 0.39±0.10 | -0.19 | 0.002 |
|  | 0.000005 | | 54.57 | *MED12L* | cg24474182 | Chr3:151047307-151047308 | Body | 0.56±0.05 | 0.37±0.10 | -0.18 | 0.0002 |
|  | 0.000005 | | 54.57 | *MED12L* | cg12207930 | Chr3:151048337-151048338 | Body; TSS1500 | 0.53±0.06 | 0.37±0.08 | -0.16 | 0.001 |
|  | 0.000005 | | 54.57 | *PARK2* | cg26872907 | Chr6:161796854-161796855 | Body | 0.33±0.07 | 0.13±0.05 | -0.20 | 0.001 |
|  | 0.000005 | | 54.57 | *PARK2* | cg23244761 | Chr6:161796850-161796851 | Body | 0.48±0.07 | 0.23±0.07 | -0.25 | 0.0002 |
|  | 0.000005 | | 54.57 | *PRKCE* | cg02073763 | Chr2:46064034-46064035 | Body | 0.42±0.06 | 0.25±0.07 | -0.17 | 0.001 |
|  | 0.000005 | | 54.57 | *PVT1* | cg23295629 | Chr8:128888486-128888487 | Body | 0.61±0.10 | 0.77±0.06 | 0.15 | 0.02 |
| myocardial infarction | 0.0001 | | 26.68 | *AUH* | cg13932501 | Chr9:94060853-94060854 | Body | 0.47±0.07 | 0.28±0.11 | -0.19 | 0.001 |
|  | 0.0001 | | 26.68 | *CASQ2* | cg14985891 | Chr1:116270626-116270627 | Body | 0.46±0.07 | 0.30±0.12 | -0.16 | 0.003 |
|  | 0.0001 | | 26.68 | *CDKAL1* | cg27224478 | Chr6:20620920-20620921 | Body | 0.78±0.08 | 0.62±0.08 | -0.16 | 0.0001 |
|  | 0.0001 | | 26.68 | *CUX1* | cg25711558 | Chr7:101499638-101499639 | Body | 0.31±0.08 | 0.10±0.03 | -0.21 | 0.001 |
|  | 0.0001 | | 26.68 | *DIP2C* | cg16072823 | Chr10:549040-549041 | Body | 0.83±0.08 | 0.68±0.10 | -0.15 | 0.002 |
|  | 0.0001 | | 26.68 | *DOCK2* | cg00357551 | Chr5:169407472-169407473 | 5'UTR | 0.45±0.05 | 0.26±0.07 | -0.19 | 0.0001 |
|  | 0.0001 | | 26.68 | *DOCK5* | cg27185978 | Chr8:25095288-25095289 | Body | 0.50±0.07 | 0.31±0.08 | -0.18 | 0.00001 |
|  | 0.0001 | | 26.68 | *ELMO1* | cg14431020 | Chr7:37256235-37256236 | Body | 0.67±0.10 | 0.47±0.03 | -0.20 | 0.001 |
|  | 0.0001 | | 26.68 | *FHIT* | cg01556706 | Chr3:59804435-59804436 | Body | 0.60±0.03 | 0.45±0.07 | -0.16 | 0.00004 |
|  | 0.0001 | | 26.68 | *KCNMA1* | cg00986133 | Chr10:78647640-78647641 | Body | 0.60±0.06 | 0.43±0.09 | -0.17 | 0.01 |
|  | 0.0001 | | 26.68 | *LHFPL2* | cg14114546 | Chr5:77823064-77823065 | 5'UTR | 0.30±0.06 | 0.15±0.04 | -0.15 | 0.0001 |
|  | 0.0001 | | 26.68 | *LHFPL2* | cg18281939 | Chr5:77783895-77783896 | 3'UTR | 0.45±0.08 | 0.30±0.09 | -0.15 | 0.005 |
|  | 0.0001 | | 26.68 | *LPCAT1* | cg22185977 | Chr5:1518133-1518134 | Body | 0.42±0.05 | 0.25±0.08 | -0.17 | 0.001 |
|  | 0.0001 | | 26.68 | *LRP5* | cg12016746 | Chr11:68084453-68084454 | Body | 0.41±0.07 | 0.22±0.06 | -0.19 | 0.002 |
|  | 0.0001 | | 26.68 | *MAD1L1* | cg22963979 | Chr7:1858916-1858917 | Body | 0.34±0.09 | 0.15±0.05 | -0.19 | 0.001 |
|  | 0.0001 | | 26.68 | *MAD1L1* | cg07027305 | Chr7:2059796-2059797 | Body | 0.35±0.05 | 0.19±0.05 | -0.16 | 0.002 |
|  | 0.0001 | | 26.68 | *MBP* | cg16604566 | Chr18:74845829-74845830 | TSS1500 | 0.34±0.05 | 0.18±0.06 | -0.16 | 0.0003 |
|  | 0.0001 | | 26.68 | *MICAL2* | cg04468741 | Chr11:12181467-12181468 | 5'UTR | 0.54±0.06 | 0.34±0.07 | -0.20 | 0.001 |
|  | 0.0001 | | 26.68 | *NCAM1* | cg11946963 | Chr11:113143736-113143737 | Body | 0.82±0.09 | 0.66±0.05 | -0.16 | 0.003 |
|  | 0.0001 | | 26.68 | *SGK1* | cg08550353 | Chr6:134497627-134497628 | TSS1500 | 0.28±0.08 | 0.10±0.03 | -0.17 | 0.001 |
|  | 0.0001 | | 26.68 | *ST6GALNAC3* | cg11348257 | Chr1:76556226-76556227 | Body | 0.34±0.04 | 0.19±0.04 | -0.15 | 0.00002 |
|  | 0.0001 | | 26.68 | *WIPI2* | cg20367388 | Chr7:5258485-5258486 | Body | 0.38±0.06 | 0.19±0.10 | -0.19 | 0.0004 |
|  | 0.0001 | | 26.68 | *WIPI2* | cg26660312 | Chr7:5271480-5271481 | 3'UTR | 0.36±0.08 | 0.16±0.06 | -0.20 | 0.00002 |
| stroke | 0.0001 | | 23.54 | *ATP10A* | cg07986058 | Chr15:26094704-26094705 | Body | 0.39±0.08 | 0.15±0.03 | -0.24 | 0.0002 |
|  | 0.0001 | | 23.54 | *ATP8B4* | cg13399903 | Chr15:50352288-503552289 | Body | 0.42±0.10 | 0.25±0.08 | -0.17 | 0.002 |
|  | 0.0001 | | 23.54 | *BACH2* | cg25670076 | Chr6:90807675-90807676 | 5'UTR | 0.70±0.05 | 0.54±0.10 | -0.16 | 0.003 |
|  | 0.0001 | | 23.54 | *CACNB2* | cg04764839 | Chr10:18708076-1870877 | Body | 0.609652 | 0.30847611 | -0.301 | 0.0001 |
|  | 0.0001 | | 23.54 | *CDKAL1* | cg27224478 | Chr6:20620920-20620921 | Body | 0.78±0.08 | 0.62±0.08 | -0.16 | 0.0001 |
|  | 0.0001 | | 23.54 | *CUX1* | cg25711558 | Chr7:101499638-101499639 | Body | 0.31±0.08 | 0.10±0.03 | -0.21 | 0.001 |
|  | 0.0001 | | 23.54 | *DOCK2* | cg00357551 | Chr5:169407472-169407473 | 5'UTR | 0.45±0.05 | 0.26±0.07 | -0.19 | 0.0001 |
|  | 0.0001 | | 23.54 | *DTNBP1* | cg26878947 | Chr6:15658832-15658833 | Body | 0.28±0.08 | 0.11±0.04 | -0.17 | 0.001 |
|  | 0.0001 | | 23.54 | *EBF1* | cg00210856 | Chr5:158466226-158466227 | Body | 0.30±0.06 | 0.13±0.04 | -0.17 | 0.0001 |
|  | 0.0001 | | 23.54 | *FHIT* | cg01556706 | Chr3:59804435-59804436 | Body | 0.60±0.03 | 0.45±0.07 | -0.16 | 0.00004 |
|  | 0.0001 | | 23.54 | *HHLA2* | cg08817540 | Chr3:108020727-108020728 | TSS1500 | 0.28±0.09 | 0.10±0.04 | -0.18 | 0.003 |
|  | 0.0001 | | 23.54 | *MICAL2* | cg04468741 | Chr11:12181467-12181468 | 5'UTR | 0.54±0.06 | 0.34±0.07 | -0.2 | 0.001 |
|  | 0.0001 | | 23.54 | *NFIC* | cg14101485 | Chr19:3369759-3369760 | Body | 0.27±0.11 | 0.10±0.04 | -0.17 | 0.01 |
|  | 0.0001 | | 23.54 | *NFIC* | cg03281139 | Chr19:3451135-3451136 | Body | 0.36±0.07 | 0.17±0.05 | -0.19 | 0.001 |
|  | 0.0001 | | 23.54 | *NFIC* | cg25826463 | Chr19:3369820-3369821 | Body | 0.43±0.05 | 0.27±0.07 | -0.16 | 0.001 |
|  | 0.0001 | | 23.54 | *PAN3* | cg17426969 | Chr13:28803434-28803435 | Body | 0.81±0.03 | 0.64±0.09 | -0.18 | 0.0002 |
|  | 0.0001 | | 23.54 | *PCNXL2* | cg17894435 | Chr1:233426334-233426335 | Body | 0.50±0.10 | 0.31±0.06 | -0.20 | 0.003 |
|  | 0.0001 | | 23.54 | *PEPD* | cg16896911 | Chr19:33895690-33895691 | Body | 0.43±0.05 | 0.27±0.06 | -0.15 | 0.001 |
|  | 0.0001 | | 23.54 | *PITNC1* | cg23791626 | Chr17:65542892-65542893 | Body | 0.49±0.06 | 0.30±0.07 | -0.19 | 0.001 |
|  | 0.0001 | | 23.54 | *REEP5* | cg03556021 | Chr5:112258649-112258650 | TSS1500 | 0.37±0.05 | 0.21±0.03 | -0.15 | 0.001 |
|  | 0.0001 | | 23.54 | *RIN2* | cg09035925 | Ch20:19915769-19915770 | Body | 0.30±0.06 | 0.11±0.04 | -0.19 | 0.0003 |
|  | 0.0001 | | 23.54 | *SNX29* | cg01990910 | Chr16:12207648-12207649 | Body | 0.28±0.06 | 0.11±0.03 | -0.18 | 0.0005 |
|  | 0.0001 | | 23.54 | *SSBP3* | cg02927682 | Chr1:54844424-54844425 | Body | 0.36±0.09 | 0.19±0.07 | -0.17 | 0.002 |
|  | 0.0001 | | 23.54 | *TAF5L* | cg04128065 | Chr1:229733305-229733306 | Body | 0.54±0.08 | 0.31±0.08 | -0.23 | 0.0005 |
|  | 0.0001 | | 23.54 | *ZAK* | cg11119767 | Chr2:174024669-174024670 | Body | 0.42±0.06 | 0.26±0.09 | -0.15 | 0.0002 |
| insulin resistance | 0.0001 | | 49.04 | *ADAM10* | cg02816525 | Chr15:59024033-59024034 | Body | 0.51±0.10 | 0.30±0.04 | -0.21 | 0.001 |
|  | 0.0001 | | 49.04 | *CACNA1D* | cg13414750 | Chr3:53782863-53782864 | Body | 0.60±0.08 | 0.37±0.04 | -0.23 | 0.001 |
|  | 0.0001 | | 49.04 | *CACNA1D* | cg21503701 | Chr3:53781065-53751066 | Body | 0.72±0.07 | 0.53±0.10 | -0.19 | 0.002 |
|  | 0.0001 | | 49.04 | *GALNTL6* | cg09232805 | Chr4:173935615-173935616 | Body | 0.37±0.04 | 0.21±0.08 | -0.16 | 0.0003 |
|  | 0.0001 | | 49.04 | *HHLA2* | cg08817540 | Chr3:108020727-108020728 | TSS1500 | 0.28±0.09 | 0.10±0.04 | -0.18 | 0.003 |
|  | 0.0001 | | 49.04 | *ITPR1* | cg02105211 | Chr3:4625188-4625189 | Body | 0.63±0.05 | 0.47±0.10 | -0.15 | 0.001 |
|  | 0.0001 | | 49.04 | *ITPR1* | cg12320198 | Chr3:4557437-4557438 | 5'UTR | 0.78±0.09 | 0.58±0.09 | -0.2 | 0.001 |
|  | 0.0001 | | 49.04 | *ITPR1* | cg02799411 | Chr3:4794061-4794062 | Body | 0.32±0.04 | 0.16±0.08 | -0.14 | 0.004 |
|  | 0.0001 | | 49.04 | *ITPR1* | cg11600734 | Chr3:4794020-4794021 | Body | 0.36±0.05 | 0.16±0.07 | -0.2 | 0.0003 |
|  | 0.0001 | | 49.04 | *PARK2* | cg26872907 | Chr6:161796854-161796855 | Body | 0.33±0.07 | 0.13±0.05 | -0.20 | 0.001 |
|  | 0.0001 | | 49.04 | *PARK2* | cg23244761 | Chr6:161796850-161796851 | Body | 0.48±0.07 | 0.23±0.07 | -0.25 | 0.0002 |
|  | 0.0001 | | 49.04 | *PFKP* | cg03068346 | Chr10:3113919-3113920 | Body | 0.62±0.05 | 0.46±0.05 | -0.16 | 0.0003 |
|  | 0.0001 | | 49.04 | *TRRAP* | cg07213487 | Chr7:98603561-98603562 | Body | 0.56±0.06 | 0.39±0.08 | -0.17 | 0.001 |
|  | 0.0001 | | 49.04 | *TRRAP* | cg01827012 | Chr7:98603440-98603441 | Body | 0.56±0.06 | 0.38±0.08 | -0.18 | 0.0004 |
|  | 0.0001 | | 49.04 | *TRRAP* | cg10154880 | Chr7:98603502-98603503 | Body | 0.50±0.06 | 0.31±0.07 | -0.19 | 0.0003 |
|  | 0.0001 | | 49.04 | *TSSC1* | cg17494897 | Chr2:3200012-3200013 | Body | 0.60±0.06 | 0.44±0.11 | -0.16 | 0.003 |
| coronary artery disease | 0.001 | | 20.29 | *ANKS1A* | cg04739880 | Chr6:35017865-35017866 | Body | 0.37±0.05 | 0.20±0.04 | -0.17 | 0.0002 |
|  | 0.001 | | 20.29 | *BACH2* | cg25670076 | Chr6:90807675-90807676 | 5'UTR | 0.70±0.05 | 0.54±0.10 | -0.16 | 0.003 |
|  | 0.001 | | 20.29 | *C10ORF11* | cg03458695 | Chr10:78163703-78163704 | Body | 0.57±0.07 | 0.39±0.10 | -0.18 | 0.001 |
|  | 0.001 | | 20.29 | *EBF1* | cg00210856 | Chr5:158466226-158466227 | Body | 0.30±0.06 | 0.13±0.04 | -0.17 | 0.0001 |
|  | 0.001 | | 20.29 | *FHIT* | cg01556706 | Chr3:59804435-59804436 | Body | 0.60±0.03 | 0.45±0.07 | -0.16 | 0.00004 |
|  | 0.001 | | 20.29 | *FNDC3B* | cg06740950 | Chr3:171878318-171878319 | Body | 0.32±0.09 | 0.12±0.02 | -0.20 | 0.001 |
|  | 0.001 | | 20.29 | *FNDC3B* | cg26074575 | Chr3:171764199-171764200 | 5'UTR | 0.76±0.09 | 0.60±0.06 | -0.16 | 0.001 |
|  | 0.001 | | 20.29 | *GOLGA3* | cg23755933 | Chr12:133349383-133349384 | 3'UTR | 0.68±0.07 | 0.52±0.09 | -0.16 | 0.0001 |
|  | 0.001 | | 20.29 | *INPP5D* | cg14012546 | Chr2:233981788-233981789 | Body | 0.50±0.05 | 0.34±0.07 | -0.16 | 0.001 |
|  | 0.001 | | 20.29 | *INPP5D* | cg22666015 | Chr2:233981885-233981886 | Body | 0.41±0.05 | 0.23±0.09 | -0.18 | 0.002 |
|  | 0.001 | | 20.29 | *LIPA* | cg12555086 | Chr10:91002590-91002591 | Body | 0.48±0.05 | 0.31±0.09 | -0.17 | 0.002 |
|  | 0.001 | | 20.29 | *LY86* | cg19190593 | Chr6:6588737-6588738 | TSS200 | 0.48±0.08 | 0.24±0.05 | -0.24 | 0.0003 |
|  | 0.001 | | 20.29 | *PARK2* | cg26872907 | Chr6:161796854-161796855 | Body | 0.33±0.07 | 0.13±0.05 | -0.20 | 0.001 |
|  | 0.001 | | 20.29 | *PARK2* | cg23244761 | Chr6:161796850-161796851 | Body | 0.48±0.07 | 0.23±0.07 | -0.25 | 0.000222 |
|  | 0.001 | | 20.29 | *PTPRG* | cg03856153 | Chr3:61904580-61904581 | Body | 0.77±0.08 | 0.62±0.06 | -0.15 | 0.004 |
|  | 0.001 | | 20.29 | *RBM47* | cg08949974 | Chr4:40632860-40632861 | TSS1500 | 0.30±0.07 | 0.15±0.04 | -0.15 | 0.002 |
|  | 0.001 | | 20.29 | *RBM47* | cg22056336 | Chr4:40444001-40444002 | 5'UTR | 0.65±0.05 | 0.36±0.10 | -0.29 | 0.001 |
|  | 0.001 | | 20.29 | *ST3GAL4* | cg09989037 | Chr1:44300942-44300943 | Body | 0.35±0.05 | 0.19±0.05 | -0.16 | 0.0001 |
|  | 0.001 | | 20.29 | *ST3GAL4* | cg19636224 | Chr1:44176844-44176845 | 5'UTR | 0.51±0.08 | 0.31±0.09 | -0.20 | 0.0005 |
|  | 0.001 | | 20.29 | *VGLL4* | cg25357825 | Chr3:11697138-11697139 | Body | 0.35±0.07 | 0.19±0.02 | -0.16 | 0.0004 |
|  | 0.001 | | 20.29 | *WDR49* | cg18517055 | Chr17:80581701-80581702 | Body | 0.61±0.07 | 0.42±0.08 | -0.19 | 0.001 |
|  | 0.001 | | 20.29 | *ZNF264* | cg05341610 | Chr19:57701430-57701431 | TSS1500 | 0.48±0.06 | 0.26±0.05 | -0.22 | 0.0001 |
| diabetes mellitus type 2 | 0.001 | | 29.05 | *AP3S2* | cg23007087 | Chr15:90380258-90380259 | Body | 0.51±0.05 | 0.32±0.11 | -0.19 | 0.001 |
|  | 0.001 | | 29.05 | *CDKAL1* | cg27224478 | Chr6:20620920-20620921 | Body | 0.78±0.08 | 0.62±0.08 | -0.16 | 0.0001 |
|  | 0.001 | | 29.05 | *FGGY* | cg19746186 | Chr1:60158474-60158475 | Body | 0.37±0.06 | 0.21±0.08 | -0.16 | 0.0002 |
|  | 0.001 | | 29.05 | *GPD2* | cg09290120 | Chr2:157291759-157291760 | TSS1500 | 0.61±0.03 | 0.42±0.08 | -0.20 | 0.001 |
|  | 0.001 | | 29.05 | *GPD2* | cg14754555 | Chr2:157292018-157292019 | 1st Exon | 0.42±0.09 | 0.24±0.05 | -0.19 | 0.002 |
|  | 0.001 | | 29.05 | *GPD2* | cg22033586 | Chr2:157292113-157292114 | 1st Exon | 0.33±0.07 | 0.17±0.04 | -0.16 | 0.001 |
|  | 0.001 | | 29.05 | *MAEA* | cg24597363 | Chr4:1294996-1294997 | Body | 0.47±0.07 | 0.31±0.11 | -0.16 | 0.00200 |
|  | 0.001 | | 29.05 | *PCNXL2* | cg17894435 | Chr1:233426334-233426335 | Body | 0.50±0.10 | 0.31±0.06 | -0.20 | 0.00300 |
|  | 0.001 | | 29.05 | *PEPD* | cg16896911 | Chr19:33895690-33895691 | Body | 0.43±0.05 | 0.27±0.06 | -0.15 | 0.001 |
|  | 0.001 | | 29.05 | *PEX5L* | cg23499115 | Chr3:179590806-17950807 | Body | 0.75±0.09 | 0.57±0.06 | -0.18 | 0.00020 |
|  | 0.001 | | 29.05 | *PPARG* | cg18637222 | Chr3:12435731-12435732 | Body | 0.59±0.08 | 0.43±0.10 | -0.16 | 0.001 |
|  | 0.001 | | 29.05 | *PTPRG* | cg03856153 | Chr3:61904580-61904581 | Body | 0.77±0.08 | 0.62±0.06 | -0.15 | 0.004 |
|  | 0.001 | | 29.05 | *TCF7L2* | cg05923857 | Chr10:114911615-11411616 | Body | 0.45±0.07 | 0.22±0.09 | -0.23 | 0.0003 |
|  | 0.001 | | 29.05 | *TCF7L2* | cg24788483 | Chr10:114911652-114911653 | Body | 0.47±0.07 | 0.27±0.05 | -0.20 | 0.00003 |
