# Supplementary material for: Shifts in the immunoepigenomic landscape of monocytes in response to a diabetes-specific social support intervention: a pilot study among Native Hawaiian adults with diabetes
Source: Clin Epigenetics. 2022 Jul 18;14:91. doi: 10.1186/s13148-022-01307-6 (PMC9295496; doi:10.1186/s13148-022-01307-6)
Supplement: Supplementary file 5 — Additional file 5: Table S4. Gene ontology analysis of differentially expressed genes. [file 13148_2022_1307_MOESM5_ESM.docx]

| **Supplementary Table 4. Intervention-Associated Differentially Expressed Genes GO Analysis** | | | | | | |  |  |  |
| --- | --- | --- | --- | --- | --- | --- | --- | --- | --- |
|  |  |  |  |  |  |  |  | |  |
| **NCI Nature 2016 Pathways** | | | | | | |  | |  |
|  |  |  |  |  |  |  |  | |  |
| **Term** | **GO *P*-value** | **Log10 P-Value** | **Z-score** | **Combined Score** | | **Gene(s)** |  |  |  |
| HIF-1-alpha transcription factor network | 0.00000002 | 7.65 | -1.45 | 25.54 | NCOA1;JUN;EGLN3;PFKFB3;TFRC;CXCR4;FURIN;ADM;HIF1A;VEGFA;NT5E;ID2;BHLHE40;EP300;PLIN2;MCL1 | |  | | |
| TCR signaling in naive CD4+ T cells | 0.000001 | 6.18 | -1.67 | 23.75 | ITK;CD3G;SLA2;CBL;CD3E;RASGRP1;CD3D;NRAS;GRAP2;CD28;FLNA;PLCG1;IKBKG;NCK1 | |  | | |
| TCR signaling in naive CD8+ T cells | 0.000003 | 5.55 | -1.55 | 19.77 | NRAS;CD8B;CD8A;GRAP2;CD28;CD3G;PLCG1;IKBKG;CBL;CD3E;RASGRP1;CD3D | |  | | |
| C-MYB transcription factor network | 0.000003 | 5.53 | -1.46 | 18.59 | CDKN1A;CBX4;LEF1;GATA3;PAX5;IQGAP1;PTGS2;NLK;ETS2;NRAS;CA1;MAT2A;MYC;BCL2;EP300 | |  | | |
| Validated targets of C-MYC transcriptional repression | 0.00002 | 4.73 | -1.45 | 15.84 | ITGB1;CDKN1A;MAX;CCL5;ID2;MYC;FTH1;BCL2;EP300;ITGA6;CLU;ZFP36L1 | |  | | |
| Integrin-linked kinase signaling | 0.00002 | 4.65 | -1.33 | 14.19 | PPP1R14A;JUN;ZEB1;ACTN1;SNAI1;CTNNB1;IQGAP1;PARVB;MYL9;LIMS1 | |  | | |
| Signaling events mediated by focal adhesion kinase | 0.00004 | 4.37 | -1.61 | 16.19 | ITGB1;RAP1B;JUN;MMP14;ITGB5;ACTN1;PLCG1;TLN1;ARHGAP26;VCL;NCK1 | |  | | |
| Validated targets of C-MYC transcriptional activation | 0.00004 | 4.37 | -1.31 | 13.14 | TFRC;MAX;ODC1;SUPT3H;FOSL1;MYCT1;KAT5;ID2;MYC;SNAI1;EP300;PMAIP1;EIF4G1 | |  | | |
| Regulation of nuclear beta catenin signaling and target gene transcription | 0.0002 | 3.73 | -1.30 | 11.20 | JUN;ID2;MYC;CAMK4;LEF1;TCF7;CTNNB1;EP300;HBP1;AXIN2;KLF4;YWHAH | |  | | |
| AP-1 transcription factor network | 0.0002 | 3.66 | -1.46 | 12.29 | FOSL1;EGR1;JUN;DMTF1;MYC;FOSB;CTNNB1;EP300;HIF1A;ATF3;FOSL2 | |  | | |
| S1P3 pathway | 0.0002 | 3.65 | -0.83 | 7.00 | GNA13;GNAZ;GNA15;ITGB3;S1PR1;CXCR4;VEGFA | |  | | |
| S1P1 pathway | 0.0002 | 3.63 | -0.29 | 2.46 | GNAZ;ITGB3;S1PR1;PLCG1;PTGS2;VEGFA | |  | | |
| Syndecan-4-mediated signaling events | 0.0003 | 3.46 | -0.50 | 3.94 | ITGB1;SDC4;CCL5;ACTN1;CXCR4;TFPI;THBS1 | |  | | |
| Stabilization and expansion of the E-cadherin adherens junction | 0.0004 | 3.41 | -0.81 | 6.37 | EGF;ACTN1;ABI1;CTNNB1;KIFC3;AQP3;VCL;NCK1 | |  | | |
| CXCR4-mediated signaling events | 0.0005 | 3.31 | -1.10 | 8.38 | BLK;ITGB1;GNAZ;CXCR4;CD3G;ARRB2;CD3E;CD3D;PTK2;RAP1B;GNA13;RGS1;ITGA6 | |  | | |
| PDGFR-beta signaling pathway | 0.001 | 3.24 | -1.29 | 9.59 | BLK;JUN;ITGB3;PTPRJ;IQGAP1;CBL;RAP1B;NRAS;CTTN;MYC;ABI1;S1PR1;PLCG1;YWHAH;NCK1 | |  | | |
| Downstream signaling in naive CD8+ T cells | 0.001 | 3.19 | -0.87 | 6.41 | FOSL1;EGR1;NRAS;JUN;CD8B;CD8A;IL2RA;CD3G;CD3E;CD3D | |  | | |
| Arf6 signaling events | 0.001 | 3.12 | -0.31 | 2.23 | GNA15;EGF;ITGB3;ITGA2B;ARRB2;ADRB2;NCK1 | |  | | |
| Signaling events mediated by VEGFR1 and VEGFR2 | 0.001 | 3.09 | -0.92 | 6.55 | ARF1;ITGB3;CTNNB1;PTPRJ;PLCG1;IQGAP1;CBL;VCL;NCK1;VEGFA | |  | | |
| Calcineurin-regulated NFAT-dependent transcription in lymphocytes | 0.001 | 3.06 | -0.54 | 3.82 | FOSL1;EGR1;JUN;CD40LG;IL2RA;DGKA;GATA3;PTGS2 | |  | | |
| Regulation of nuclear SMAD2/3 signaling | 0.001 | 3.04 | -0.73 | 5.12 | NCOA1;CDKN1A;JUN;ITGB5;MAX;MYC;EP300;GATA3;SKIL;ATF3;SMAD7 | |  | | |
| Notch signaling pathway | 0.001 | 2.95 | -0.53 | 3.59 | NOTCH2;CDKN1A;NCSTN;MYC;EP300;FURIN;GATA3;CBL;RAB11A | |  | | |
| Nectin adhesion pathway | 0.002 | 2.82 | 0.41 | -2.68 | RAP1B;ITGB3;CTNNB1;IQGAP1;TLN1;F11R | |  | | |
| IL12 signaling mediated by STAT4 | 0.003 | 2.59 | 0.23 | -1.38 | JUN;IL2RA;CD28;CD3G;CD3E;CD3D | |  | | |
| Beta3 integrin cell surface interactions | 0.003 | 2.57 | -0.08 | 0.49 | SDC4;ITGB3;ITGA2B;PLAUR;F11R;THBS1;VEGFA | |  | | |
| IL2-mediated signaling events | 0.003 | 2.54 | -0.53 | 3.11 | SOCS3;NRAS;JUN;CISH;IL2RA;MYC;BCL2;IKZF3 | |  | | |
| C-MYC pathway | 0.003 | 2.52 | 2.10 | -12.23 | KAT5;MAX;MYC;HBP1;SUPT3H | |  | | |
| Direct p53 effectors | 0.003 | 2.52 | -0.50 | 2.92 | PLK3;CDKN1A;BTG2;JUN;CCNK;PRDM1;BCL6;DDIT4;BCL2;EP300;PMAIP1;ATF3;MCL1;HSPA1A | |  | | |
| Trk receptor signaling mediated by the MAPK pathway | 0.004 | 2.45 | 0.56 | -3.17 | RAP1B;EGR1;RIT1;NRAS;RPS6KA5;MAPK7 | |  | | |
| HIF-2-alpha transcription factor network | 0.004 | 2.45 | 0.67 | -3.76 | EGLN3;MMP14;BHLHE40;SLC11A2;EP300;VEGFA | |  | | |
| Validated transcriptional targets of AP1 family members Fra1 and Fra2 | 0.004 | 2.38 | 0.68 | -3.75 | FOSL1;THBD;JUN;DMTF1;PLAUR;EP300 | |  | | |
| IL4-mediated signaling events | 0.005 | 2.31 | -0.24 | 1.29 | SELP;SOCS3;FCER2;IL4R;CD40LG;BCL6;ITGB3;CBL | |  | | |
| Integrin family cell surface interactions | 0.01 | 2.28 | 1.96 | -10.27 | ITGB1;ITGB5;ITGB3;ITGA2B;ITGA6 | |  | | |
| IL12-mediated signaling events | 0.01 | 2.22 | -0.03 | 0.17 | CD8B;RIPK2;CD8A;IL2RA;CCL3;CD3G;CD3E;CD3D | |  | | |
| VEGFR1 specific signals | 0.01 | 2.21 | 1.30 | -6.60 | PLCG1;CBL;HIF1A;NCK1;VEGFA | |  | | |
| Signaling events mediated by Hepatocyte Growth Factor Receptor (c-Met) | 0.01 | 2.15 | -0.32 | 1.59 | RAP1B;EGR1;JUN;SNAI1;CTNNB1;PTPRJ;PLCG1;CBL;NCK1 | |  | | |
| amb2 Integrin signaling_Homo sapiens | 0.01 | 2.09 | 0.58 | -2.80 | BLK;SELP;RAP1B;PLAUR;TLN1;JAM3 | |  | | |
| Calcium signaling in the CD4+ TCR pathwa | 0.01 | 2.07 | 2.13 | -10.18 | FOSL1;JUN;CD40LG;IL2RA;PTGS2 | |  | | |
| Beta2 integrin cell surface interactions | 0.01 | 2.07 | 2.62 | -12.52 | CD40LG;PLAUR;GP1BA;F11R;JAM3 | |  | | |
| Glucocorticoid receptor regulatory network | 0.01 | 1.97 | -0.16 | 0.72 | NCOA1;NR4A1;EGR1;CDKN1A;JUN;EP300;GATA3;PBX1;YWHAH | |  | | |
| Thromboxane A2 receptor signaling | 0.01 | 1.93 | 0.26 | -1.17 | BLK;GNA13;GNA15;EGF;TBXA2R;ARRB2;RAB11A | |  | | |
| Sphingosine 1-phosphate (S1P) pathway | 0.01 | 1.89 | 3.11 | -13.57 | GNA13;GNAZ;GNA15;S1PR1 | |  | | |
| Ceramide signaling pathway | 0.01 | 1.89 | 0.76 | -3.33 | ASAH1;EGF;SMPD1;MYC;BCL2;PDGFA | |  | | |
| ATF-2 transcription factor network | 0.01 | 1.85 | 0.41 | -1.76 | SOCS3;JUN;DUSP10;KAT5;BCL2;EP300;ATF3 | |  | | |
| EGF receptor (ErbB1) signaling pathway | 0.02 | 1.78 | 1.14 | -4.67 | NRAS;EGF;PLCG1;TLN1;NCK1 | |  | | |
| IL8- and CXCR2-mediated signaling events | 0.02 | 1.78 | 1.71 | -7.04 | GNA15;ARRB2;CBL;DOCK2;RAB11A | |  | | |
| Signaling events mediated by PRL | 0.02 | 1.75 | 2.77 | -11.20 | ITGB1;EGR1;CDKN1A;TUBA1B | |  | | |
| S1P2 pathway | 0.02 | 1.69 | 2.91 | -11.31 | GNA13;GNAZ;GNA15;JUN | |  | | |
| Trk receptor signaling mediated by PI3K and PLC-gamma | 0.02 | 1.68 | 1.48 | -5.74 | EGR1;NRAS;CAMK4;PLCG1;YWHAH | |  | | |
| Arf6 trafficking events | 0.02 | 1.68 | 1.05 | -4.05 | ITGB1;IL2RA;CTNNB1;ITGA6;ASAP2;ADRB2 | |  | | |
| S1P4 pathway | 0.02 | 1.65 | 3.88 | -14.78 | GNA13;GNAZ;PLCG1 | |  | | |
| BCR signaling pathway | 0.02 | 1.63 | 0.54 | -2.03 | CD79B;CD79A;JUN;PPP3CC;IKBKG;CD22;NFKBIB | |  | | |
| Alpha9 beta1 integrin signaling events | 0.02 | 1.63 | 4.43 | -16.61 | ITGB1;F13A1;SAT1;VEGFA | |  | | |
| LPA receptor mediated events | 0.03 | 1.60 | 0.60 | -2.20 | GNA13;GNAZ;GNA15;JUN;PLCG1;PTK2;HBEGF | |  | | |
| Signaling events mediated by HDAC Class III | 0.03 | 1.57 | 4.20 | -15.22 | CDKN1A;TUBA1B;EP300;ACSS1 | |  | | |
| Arf6 downstream pathway | 0.03 | 1.57 | 5.06 | -18.29 | ARF1;PLAUR;RAB11A | |  | | |
| ErbB receptor signaling network | 0.03 | 1.57 | 6.03 | -21.81 | EGF;AREG;HBEGF | |  | | |
| Circadian rhythm pathway | 0.03 | 1.57 | 8.81 | -31.85 | PER1;BHLHE40;NR1D1 | |  | | |
| E-cadherin signaling in the nascent adherens junction | 0.03 | 1.55 | 2.27 | -8.08 | RAP1B;CTTN;ABI1;CTNNB1;IQGAP1 | |  | | |
| Plasma membrane estrogen receptor signaling_ | 0.03 | 1.50 | 1.86 | -6.43 | GNA13;GNAZ;GNA15;NRAS;HBEGF | |  | | |
| Internalization of ErbB1 | 0.03 | 1.50 | 1.91 | -6.62 | NRAS;EGF;UBE2D2;LRIG1;CBL | |  | | |
| Retinoic acid receptors-mediated signaling | 0.03 | 1.47 | 3.44 | -11.62 | NCOA1;CCNH;NRIP1;EP300 | |  | | |
| Syndecan-3-mediated signaling events | 0.04 | 1.43 | 7.93 | -26.03 | NCSTN;CTTN;CASK | |  | | |
| CD40/CD40L signaling | 0.04 | 1.37 | 3.53 | -11.13 | JUN;CD40LG;MYC;TNFAIP3 | |  | | |
| Ephrin B reverse signaling | 0.04 | 1.37 | 3.66 | -11.53 | BLK;ITGB3;ITGA2B;CXCR4 | |  | | |
| Fc-epsilon receptor I signaling in mast cells | 0.04 | 1.36 | 1.34 | -4.21 | ITK;JUN;S1PR1;PLCG1;IKBKG;CBL | |  | | |
| ErbB1 downstream signaling | 0.04 | 1.35 | 1.27 | -3.94 | EGR1;NRAS;JUN;RPS6KA5;MAPK7;EGF;ABI1;IQGAP1;YWHAH | |  | | |
| FGF signaling pathway | 0.05 | 1.33 | 1.44 | -4.39 | JUN;SDC4;CTTN;PLAUR;PLCG1;CBL | |  | | |
| Nephrin/Neph1 signaling in the kidney podocyte | 0.05 | 1.32 | 3.49 | -10.65 | JUN;PLCG1;ARRB2;NCK1 | |  | | |
| a6b1 and a6b4 Integrin signaling | 0.05 | 1.31 | 1.92 | -5.81 | ITGB1;EGF;CD9;ITGA6;YWHAH | |  | | |
| **GO Biological Process 2018** | | | | | | |  | |  |
|  |  |  |  |  |  |  |  | |  |
| **Term** | **GO *P*-value** |  | **Z-score** | **Combined Score** | **Gene(s)** | |  | | |
| platelet degranulation (GO:0002576) | 0.0000000000002 | 12.82 | -1.44 | 42.38 | APP;SPARC;ITGB3;PROS1;PLEK;ITGA2B;ENDOD1;PDGFA;F13A1;THBS1;CLU;FLNA;LY6G6F;SRGN;EGF;APLP2;ACTN1;RAB27B;PPBP;TUBA4A;F5;VEGFA;SELP;CD9;TAGLN2;TLN1;VCL;PF4;FERMT3 | |  | | |
| regulated exocytosis (GO:0045055) | 0.000000000003 | 11.53 | -2.30 | 61.16 | APP;SPARC;ITGB3;PROS1;PLEK;ITGA2B;ENDOD1;PDGFA;F13A1;THBS1;CLU;FLNA;LY6G6F;SRGN;EGF;APLP2;ACTN1;RAB27B;PPBP;TUBA4A;F5;VEGFA;SELP;RAB31;CD9;TAGLN2;TLN1;VCL;PF4;FERMT3 | |  | | |
| platelet aggregation (GO:0070527) | 0.0000001 | 6.84 | -1.79 | 28.17 | P2RY12;RAP2B;ITGB3;PLEK;FLNA;MYH9;HBB;TLN1;MYL9;VCL;FERMT3 | |  | | |
| cytokine-mediated signaling pathway (GO:0019221) | 0.0000002 | 6.76 | -1.34 | 20.90 | ITGB1;ARF1;CDKN1A;CIITA;F13A1;TNFRSF13C;CXCL5;CA1;RPS6KA5;PSMD7;MYC;IKBKG;PF4V1;IL4R;CISH;TNFRSF12A;RIPK2;FLT3LG;GP1BA;FCER2;ZEB1;TNFRSF25;RHOU;LTB;PSMD14;GATA3;PTGS2;CBL;HIF1A;PSMA7;HSP90B1;RAP1B;SOCS3;HIST1H3A;CCL5;IL21R;S1PR1;CCL3;MCL1;IL32;HSPA9;EGR1;CCL20;OSM;PPBP;SOD2;VEGFA;ISG20;PSMC6;CD40LG;BCL6;TNFSF4;IL2RA;BCL2;CD27;IL7R;PF4;LIMS1 | |  | | |
| T cell activation (GO:0042110) | 0.0000003 | 6.47 | -1.33 | 19.82 | BLK;ITK;RHOH;PTPN22;CD3G;SLA2;CD3E;CD3D;DPP4;CD2;CD8B;CD8A;TNFSF4;CD28;CCR7;ICOSLG;NCK1 | |  | | |
| homotypic cell-cell adhesion (GO:0034109) | 0.000001 | 6.16 | -1.68 | 23.91 | P2RY12;RAP2B;ITGB3;PLEK;FLNA;MYH9;HBB;TLN1;MYL9;VCL;FERMT3 | |  | | |
| negative regulation of apoptotic process (GO:0043066) | 0.000001 | 5.88 | -1.06 | 14.33 | NOTCH2;ITGB1;TNFAIP8;GSTP1;LEF1;ALOX12;CBL;THBS1;HSP90B1;TCL1A;DNAJB6;GRK5;MYC;ZC3H12A;MYO18A;CD38;FLNA;PIM3;PIM2;MCL1;SNCA;BARD1;CDKN2D;HSPA9;PLK3;NAT8B;CBX4;HSPA5;ANGPT1;RIPK2;F2R;PLAUR;KLF4;SOD2;PTK2;VEGFA;DAB2;CD40LG;BCL3;CD28;BCL2;PPIF;CD27;CTNNB1;HSPA1B;HSPA1A | |  | | |
| regulation of cell proliferation (GO:0042127) | 0.000001 | 5.85 | -1.13 | 15.17 | ITK;APP;CDKN1A;BTG2;TGFB1I1;RTKN2;SIRPG;PTPRJ;SLA2;AREG;CXCL5;TCL1A;GRAP2;MYC;FTH1;NAMPT;PIM2;PF4V1;KLF11;F2R;FLT3LG;AXIN2;CDC25B;TNFRSF25;HBEGF;BLK;LEF1;NOP2;PDGFA;GATA3;THBS1;ZFP36L1;DPP4;RPS15A;GRK5;LMNA;ZNF703;CDKN2D;JUN;EGLN3;EGF;OSM;PPBP;KLF4;SOD2;PTK2;VEGFA;FOSL1;CCDC88A;NR4A3;BCL6;TNFSF4;ABI1;BHLHE40;CD27;CTNNB1;GRAP;IL7R;HSPA1B;PF4;EIF4G1;HSPA1A | |  | | |
| negative regulation of transcription, DNA-templated (GO:0045892) | 0.000002 | 5.72 | -1.75 | 23.08 | CREBZF;ZNF253;CIITA;NAB2;PRDM1;AHR;SLA2;ETS2;IRF2BPL;RPS6KA5;KAT5;MYC;CD38;EP300;JARID2;KLF11;KLF12;ZHX2;ARID5B;THAP1;HCFC1;ZEB1;ZNF318;ATF3;URI1;MAX;LEF1;PBXIP1;GATA3;NRIP1;ZNF703;FLNA;SKIL;SNCA;PLK3;CBX7;JUN;ZFHX3;CBX5;CBX4;NR1D1;KLF4;KLF2;BMP6;VEGFA;SMAD7;PER1;GFI1B;DAB2;NR4A3;BCL6;TNFSF4;ID2;BCL3;ID1;BHLHE40;SNAI1;FOSB;CTNNB1;TAF7;ZNF217;MAFK;MXD1;MPHOSPH8;GZF1;LIMS1 | |  | | |
| cellular response to transforming growth factor beta stimulus (GO:0071560) | 0.000002 | 5.66 | -1.98 | 25.76 | JUN;ITGB5;ARHGEF18;FURIN;ARRB2;F11R;CBL;NLK;PTK2;ZFP36L1;SMAD7;TWSG1;MAPK7;FNTA;ID1;ADAM9;LIMS1 | |  | | |
| T cell receptor signaling pathway (GO:0050852) | 0.000004 | 5.41 | -1.21 | 15.01 | ITK;PSMD14;BTN3A1;RIPK2;TRAT1;UBE2D2;THEMIS;PTPRJ;PTPN22;CD3G;GATA3;CD3E;CD3D;PSMA7;PSMC6;PSMD7;GRAP2;PDE4B;PLCG1;IKBKG;ICOSLG;NCK1 | |  | | |
| positive regulation of nitrogen compound metabolic process (GO:0051173) | 0.00001 | 5.15 | -2.34 | 27.75 | MTPN;APP;MAPK7;EGF;GATA3;KLF4;KLF2 | |  | | |
| negative regulation of gene expression (GO:0010629) | 0.00001 | 5.04 | -1.25 | 14.49 | CREBZF;APP;ZNF253;BTG2;CIITA;PTPN22;AHR;CD3E;RPS6KA5;KAT5;ZC3H12A;CD38;JARID2;RPS13;ZHX2;ARID5B;ZEB1;ZNF318;DCP2;NOTCH2;LEF1;PBXIP1;GATA3;HIF1A;HIST1H3A;ZNF703;CCL3;JUN;CBX5;CBX4;NR1D1;KLF4;MEX3C;VEGFA;PER1;HIST1H4A;DAB2;BCL6;TNFSF4;ID2;BCL3;ID1;BHLHE40;CD28;CTNNB1;TAF7;ZNF217;MPHOSPH8;HSPA1B;GZF1;LIMS1;HSPA1A | |  | | |
| antigen receptor-mediated signaling pathway (GO:0050851) | 0.00001 | 4.88 | -1.06 | 11.92 | BLK;ITK;PSMD14;UBE2D2;PTPRJ;PTPN22;CD3G;GATA3;SLA2;CD3E;CD3D;PSMA7;CD79B;CD79A;PSMD7;GRAP2;PDE4B;CD38;PLCG1;IKBKG;ICOSLG;NCK1;BTN3A1;RIPK2;TRAT1;THEMIS;PSMC6;BCL2 | |  | | |
| regulation of cell migration (GO:0030334) | 0.00001 | 4.83 | -1.26 | 14.02 | CLIC4;MCTP1;LEF1;NEXN;PDGFA;PTPRJ;THBS1;RHOBTB1;CORO1C;CXCL16;DUSP10;CCL5;LMNA;ZNF703;CCL3;FLNA;HSPA5;EGF;TPM1;F2R;SOD2;PTK2;VEGFA;SMAD7;VIL1;MMP14;DAB2;SNAI1;ITGA6;VCL;HBEGF;FERMT3 | |  | | |
| positive regulation of cellular process (GO:0048522) | 0.00002 | 4.76 | -1.34 | 14.72 | MTPN;LEF1;NOP2;RTKN2;SIRPG;PDGFA;HBB;AGPAT1;THBS1;AREG;CXCL5;CXCL16;DPP4;TCL1A;CNST;RPS15A;GRK5;MYC;ZC3H12A;LMNA;NAMPT;ZNF703;FLNA;JUN;EGF;RIPK2;F2R;OSM;FLT3LG;AXIN2;PTK2;CDC25B;HCFC1;VEGFA;FOSL1;P2RX5;MMP14;DAB2;TAL1;TNFSF4;BCL2;IL7R;EIF4G2;EIF4G1;HBEGF | |  | | |
| negative regulation of intrinsic apoptotic signaling pathway (GO:2001243) | 0.00002 | 4.73 | -1.33 | 14.44 | CDKN2D;URI1;MAPK7;BCL2;SNAI1;PLAUR;PPIF;HYOU1;SOD2;CLU;MCL1;HSPA1A | |  | | |
| response to lipopolysaccharide (GO:0032496) | 0.00002 | 4.68 | -1.69 | 18.24 | JUN;GSTP1;F2R;TNFAIP3;PTPN22;PPBP;NR1D1;CXCL5;SELP;CD6;TNFSF4;ZC3H12A;PDE4B;CD27;ADAM9;NLRP3;TNFRSF25;PF4V1;PF4;SNCA | |  | | |
| regulation of myeloid cell differentiation (GO:0045637) | 0.00003 | 4.52 | -1.37 | 14.21 | HSPA9;MEIS1;HIST1H4A;NR4A3;HIST1H3A;ITGA2B;EP300;DPY30;GP1BA;THBS1;MYL9;PF4 | |  | | |
| transforming growth factor beta receptor signaling pathway (GO:0007179) | 0.00003 | 4.49 | -1.46 | 15.07 | JUN;ITGB5;ARHGEF18;FURIN;ARRB2;F11R;CBL;NLK;PTK2;TWSG1;FNTA;ID1;ADAM9 | |  | | |
| negative regulation of programmed cell death (GO:0043069) | 0.00004 | 4.42 | -1.26 | 12.85 | NOTCH2;TNFAIP8;GSTP1;LEF1;CBL;THBS1;HSP90B1;TCL1A;GRK5;MYC;MYO18A;CD38;FLNA;PIM3;PIM2;MCL1;SNCA;BARD1;HSPA9;PLK3;NAT8B;CBX4;HSPA5;ANGPT1;RIPK2;PLAUR;PTK2;VEGFA;DAB2;CD40LG;BCL3;CD28;BCL2;PPIF;CD27;HSPA1B;HSPA1A | |  | | |
| cellular response to cytokine stimulus (GO:0071345) | 0.00004 | 4.41 | -1.15 | 11.67 | ITGB1;CDKN1A;DHX9;LEF1;TCF7;CXCR4;F13A1;GATA3;PTGS2;CBL;HIF1A;HSP90B1;ZFP36L1;SOCS3;TCL1A;CCL5;MYC;ZC3H12A;S1PR1;CCL3;CCR7;MCL1;IL32;PTPN18;EGR1;IL4R;CISH;CCL20;OSM;FLT3LG;GP1BA;MAPK13;VEGFA;FCER2;ZEB1;BCL6;IL2RA;BCL2;RHOU;PF4 | |  | | |
| regulation of interleukin-4 production (GO:0032673) | 0.00004 | 4.38 | -1.84 | 18.50 | CD40LG;TNFSF4;LEF1;CD28;NLRP3;GATA3 | |  | | |
| beta-catenin-TCF complex assembly (GO:1904837) | 0.00005 | 4.32 | -1.61 | 16.04 | HIST1H4A;KAT5;MYC;LEF1;TCF7;CTNNB1;EP300;AXIN2 | |  | | |
| positive regulation of T cell activation (GO:0050870) | 0.00005 | 4.32 | -1.23 | 12.28 | CD40LG;PNP;TFRC;CD6;TNFSF4;CCL5;SIRPG;CD28;CCR7;GATA3;CD3E;NCK1 | |  | | |
| positive regulation of macromolecule metabolic process (GO:0010604) | 0.00005 | 4.32 | -1.82 | 18.04 | MTPN;APP;LEF1;PTPN22;ARRB2;CD3E;HIF1A;CLU;MAPK7;MYC;ZC3H12A;CCL3;SNCA;EGR1;NAT8B;ANGPT1;EGF;EPHX2;KLF4;KLF2;HCFC1;VEGFA;ID2;CD28;HSPA1B;PF4;LIMS1;HSPA1A | |  | | |
| regulation of apoptotic process (GO:0042981) | 0.0001 | 4.27 | -1.75 | 17.18 | ITGB1;TNFAIP8;CD3E;IKZF3;CLU;TCL1A;MYC;MYO18A;CD38;PIM3;PIM2;KLF11;NAT8B;ARHGEF12;TNFRSF12A;RIPK2;ACTN1;PLAUR;ARHGEF18;ANO6;OBSCN;PPIF;ITGA6;TNFRSF25;NOTCH2;GSTP1;LEF1;CBL;THBS1;HSP90B1;GNA13;NCSTN;PNMA1;GRK5;FLNA;PMAIP1;MCL1;SNCA;BARD1;HSPA9;PLK3;JUN;EGLN3;CBX4;HSPA5;ANGPT1;HIP1R;BMP6;PTK2;VEGFA;GZMK;DAB2;CD40LG;BCL6;BCL3;CD28;BCL2;CD27;CTNNB1;HSPA1B;HSPA1A | |  | | |
| positive regulation of cell motility (GO:2000147) | 0.0001 | 4.26 | -1.21 | 11.85 | HSPA5;EGF;LEF1;F2R;PDGFA;SOD2;THBS1;PTK2;CXCL16;VEGFA;VIL1;MMP14;DAB2;CCL5;ZNF703;SNAI1;CCL3;ITGA6;CCR7;HBEGF;FERMT3 | |  | | |
| regulation of megakaryocyte differentiation (GO:0045652) | 0.0001 | 4.23 | -1.37 | 13.32 | HIST1H4A;NR4A3;HIST1H3A;ITGA2B;EP300;DPY30;GP1BA;THBS1;MYL9;PF4 | |  | | |
| positive regulation of cellular metabolic process (GO:0031325) | 0.0001 | 4.21 | -1.53 | 14.79 | CDKN1A;ANGPT1;EGF;GATA3;ARRB2;KLF4;AGPAT1;THBS1;HIF1A;VEGFA;CCL5;ZC3H12A;SNCA;EIF4G1 | |  | | |
| T cell migration (GO:0072678) | 0.0001 | 4.21 | -2.17 | 21.00 | CCL20;GPR183;S1PR1;CCL3;CCR6;CXCL16 | |  | | |
| negative regulation of transcription from RNA polymerase II promoter (GO:0000122) | 0.0001 | 4.18 | -1.06 | 10.23 | CIITA;URI1;MAX;PRDM1;SLA2;ETS2;IRF2BPL;KAT5;MYC;NRIP1;EP300;SKIL;SNCA;PLK3;KLF11;CBX7;KLF12;JUN;ZFHX3;ZHX2;CBX5;CBX4;ARID5B;NR1D1;KLF4;KLF2;BMP6;THAP1;HCFC1;VEGFA;SMAD7;PER1;GFI1B;NR4A3;ZEB1;BCL6;BHLHE40;SNAI1;FOSB;TAF7;ZNF217;MAFK;MXD1;ATF3;GZF1;HSPA1A | |  | | |
| inflammatory response (GO:0006954) | 0.0001 | 4.17 | -1.75 | 16.77 | CXCR4;F11R;THBS1;CXCL5;TBXA2R;CCL5;CCL3;NLRP3;CCR7;IKBKG;PF4V1;KLRG1;CCL20;RIPK2;PLA2G4B;EPHX2;F2R;PPBP;CD40LG;CD6;TNFSF4;IL2RA;CD27;TNFRSF25;MGLL;PF4 | |  | | |
| positive regulation of lymphocyte proliferation (GO:0050671) | 0.0001 | 4.13 | -1.43 | 13.56 | CD40LG;PNP;TFRC;CD6;GPR183;TNFSF4;CCL5;CD28;BCL2;CD38;CD3E;NCK1 | |  | | |
| transmembrane receptor protein serine/threonine kinase signaling pathway (GO:0007178) | 0.0001 | 4.09 | -2.11 | 19.91 | JUN;ITGB5;TGFB1I1;LEF1;ARHGEF18;FURIN;ARRB2;F11R;CBL;NLK;FSTL1;BMP6;PTK2;SMAD7;TWSG1;FNTA;ID1;ADAM9 | |  | | |
| cellular response to hypoxia (GO:0071456) | 0.0001 | 4.05 | -1.32 | 12.35 | PLK3;EGLN3;PSMD14;UBE2D2;PTGS2;AQP3;HIF1A;PSMA7;ZFP36L1;VEGFA;PSMC6;PSMD7;MYC;LMNA;EP300;PMAIP1;HYOU1 | |  | | |
| positive regulation of hydrolase activity (GO:0051345) | 0.0001 | 4.03 | -1.69 | 15.74 | ITGB1;CCL20;DOCK9;TPM1;F2R;F11R;RASGRP1;RGP1;ARHGAP21;FNTA;RGS1;CCL5;DNAJB11;RGS10;CCL3;ITGA6;CALM3;RSU1;RGS6;LIMS1 | |  | | |
| positive regulation of cellular biosynthetic process (GO:0031328) | 0.0001 | 4.02 | -1.85 | 17.12 | EGR1;DHX9;EGF;PDGFA;HBB;NR1D1;PTGS2;KLF4;THBS1;AREG;HIF1A;CLU;KLF2;CCL5;MYC;BCL3;CD28;LDLR;SNCA | |  | | |
| lamellipodium organization (GO:0097581) | 0.0001 | 3.97 | -1.77 | 16.20 | CCDC88A;ABLIM1;CTTN;ABLIM3;S1PR1;PARVB;VCL | |  | | |
| positive regulation of epithelial cell migration (GO:0010634) | 0.0001 | 3.95 | -1.36 | 12.40 | VIL1;SPARC;ANGPT1;ITGB3;ZC3H12A;ADAM9;PLCG1;GATA3;THBS1;HIF1A;RAB11A;VEGFA | |  | | |
| positive regulation of cell migration (GO:0030335) | 0.0002 | 3.81 | -1.86 | 16.32 | HSPA5;EGF;LEF1;F2R;PDGFA;SOD2;THBS1;HIF1A;RAB11A;PTK2;CXCL16;VEGFA;VIL1;MMP14;DAB2;CCL5;ZNF703;SNAI1;CCL3;ITGA6;PLCG1;HBEGF;FERMT3 | |  | | |
| positive regulation of blood coagulation (GO:0030194) | 0.0002 | 3.76 | -1.91 | 16.53 | SELP;THBD;PLEK;F2R;ANO6;THBS1 | |  | | |
| epidermal growth factor receptor signaling pathway (GO:0007173) | 0.0002 | 3.66 | -2.11 | 17.80 | VIL1;NRAS;RPS6KA5;EGF;PLCG1;IQGAP1;CBL;AREG;PTK2;HBEGF | |  | | |
| adherens junction assembly (GO:0034333) | 0.0002 | 3.62 | -3.17 | 26.42 | ZNF703;CTNNB1;VCL;SMAD7 | |  | | |
| response to iron ion (GO:0010039) | 0.0002 | 3.61 | -2.44 | 20.28 | SLC11A2;BCL2;HIF1A;BMP6;SNCA | |  | | |
| receptor metabolic process (GO:0043112) | 0.0003 | 3.60 | -1.51 | 12.50 | DNM3;ITGB1;EHD3;RAB31;TFRC;CD9;ADM;NSG1;ARRB2;SNCA | |  | | |
| integrin-mediated signaling pathway (GO:0007229) | 0.0003 | 3.60 | -1.39 | 11.54 | ITGB1;ITGB5;ITGB3;PLEK;ITGA2B;ADAM9;NEDD9;MYH9;PTK2;FERMT3 | |  | | |
| regulation of transcription from RNA polymerase II promoter in response to stress (GO:0043618) | 0.0003 | 3.58 | -1.27 | 10.45 | EGLN3;PSMD14;UBE2D2;HIF1A;PSMA7;KLF2;VEGFA;PSMC6;PSMD7;MAPK7;EP300;ATF3;HSPA1A | |  | | |
| T cell differentiation (GO:0030217) | 0.0003 | 3.57 | -1.89 | 15.52 | BLK;CD8A;LEF1;CD28;RHOH;PTPN22;CD3D;ZFP36L1 | |  | | |
| regulation of protein metabolic process (GO:0051246) | 0.0003 | 3.54 | -1.89 | 15.36 | MTPN;APP;MAPK7;ODC1;PLAUR;FURIN;GATA3;KLF4;LDLR;KLF2 | |  | | |
| phosphatidylserine acyl-chain remodeling (GO:0036150) | 0.0003 | 3.51 | -2.87 | 23.19 | OSBPL8;PLA2G12A;LPCAT4;PLA2G4B;LPCAT3;OSBPL10 | |  | | |
| transcription, DNA-templated (GO:0006351) | 0.0003 | 3.49 | -1.80 | 14.47 | CREBZF;CCNK;MAX;CCNH;TGFB1I1;LEF1;DDX21;GATA3;ARRB2;AHR;HIF1A;BACH2;IWS1;SNAPC3;NCOA1;KLF11;EGR1;ZFHX3;TSC22D1;EAF1;PAX5;KLF4;THAP1;HCFC1;FOSL1;GFI1B;POU2AF1;BCL3;TRIP11;TAF7;NFKBIB | |  | | |
| protein complex assembly (GO:0006461) | 0.0003 | 3.48 | -1.79 | 14.36 | ANKRD28;SEC16A;LEF1;TCF7;TNFAIP3;ANO6;AXIN2;RASGRP1;AREG;F5;HIST1H4A;KAT5;TRAPPC6B;MYC;ZC3H12A;CTNNB1;NLRP3;EP300;SEC31A;FERMT3 | |  | | |
| negative regulation of intracellular signal transduction (GO:1902532) | 0.0003 | 3.48 | -1.30 | 10.41 | TRIM40;URI1;GSTP1;PDE3B;PLEK;PLAUR;RHOH;TNFAIP3;PTPRJ;ARRB2;SLA2;THBS1;PER1;ZC3H12A;DDIT4;BCL2;PPIF;MCL1 | |  | | |
| cellular response to organic cyclic compound (GO:0071407) | 0.0004 | 3.43 | -1.26 | 9.97 | P2RY12;EGR1;URI1;AHR;TFPI;HSP90B1;ZFP36L1;RAP1B;NR4A3;KAT5;CCL5;ZC3H12A;NRIP1;ZNF703;CCL3;CTNNB1 | |  | | |
| positive regulation of cytokine production (GO:0001819) | 0.0004 | 3.43 | -1.88 | 14.84 | DHX9;RIPK2;PTPN22;GATA3;PTGS2;AGPAT1;LY9;RASGRP1;HIF1A;MAPK13;CD40LG;CD6;TNFSF4;CD28;PDE4B;CTNNB1;NLRP3;EP300;SLAMF6;CCR7;HSPA1B;HSPA1A | |  | | |
| positive regulation of leukocyte chemotaxis (GO:0002690) | 0.0004 | 3.42 | -1.48 | 11.65 | CCL5;CCL3;CCR7;ANO6;PPBP;THBS1;CXCL5;PF4V1;PF4;VEGFA | |  | | |
| positive regulation of T cell proliferation (GO:0042102) | 0.0004 | 3.42 | -1.37 | 10.75 | CD40LG;PNP;TFRC;CD6;TNFSF4;CCL5;CD28;CD3E;ICOSLG;NCK1 | |  | | |
| positive regulation of GTPase activity (GO:0043547) | 0.0004 | 3.39 | -1.16 | 9.05 | ITGB1;RABGAP1L;CCL20;DOCK9;F2R;MTSS1L;F11R;RASGRP1;CORO1C;RGP1;ARHGAP21;RGS1;CCL5;RGS10;CCL3;ITGA6;CCR7;RSU1;RGS6;LIMS1 | |  | | |
| cellular response to unfolded protein (GO:0034620) | 0.0004 | 3.39 | -2.02 | 15.77 | HSPA9;HSPH1;HSPA5;HYOU1;HSPA1B;HSPA1A | |  | | |
| negative regulation of myeloid cell differentiation (GO:0045638) | 0.0004 | 3.39 | -1.99 | 15.57 | HSPA9;MEIS1;HIST1H4A;MAFB;PF4;ZFP36L1 | |  | | |
| desensitization of G-protein coupled receptor protein signaling pathway (GO:0002029) | 0.0004 | 3.39 | -2.73 | 21.32 | ADM;ARRB2;ADRB2;LY6G6E | |  | | |
| regulation of T cell proliferation (GO:0042129) | 0.0004 | 3.36 | -1.78 | 13.80 | CD40LG;PNP;SDC4;TFRC;CD6;TNFSF4;CCL5;CD28;CD3E;NCK1 | |  | | |
| response to molecule of bacterial origin (GO:0002237) | 0.0004 | 3.35 | -1.25 | 9.62 | JUN;F2R;TNFAIP3;PTPN22;PPBP;CXCL5;SELP;CD6;CD27;TNFRSF25;PF4V1;PF4;SNCA | |  | | |
| positive regulation of receptor-mediated endocytosis (GO:0048260) | 0.0005 | 3.34 | -1.98 | 15.17 | DAB2;ANGPT1;EGF;HIP1R;ARRB2;CBL;CLU;VEGFA | |  | | |
| cellular response to tumor necrosis factor (GO:0071356) | 0.0005 | 3.34 | -1.23 | 9.44 | PSMD14;TNFRSF12A;DHX9;CCL20;GATA3;TNFRSF13C;PSMA7;ZFP36L1;TCL1A;PSMC6;PSMD7;CD40LG;TNFSF4;CCL5;ZC3H12A;CCL3;CD27;TNFRSF25;LTB;LIMS1 | |  | | |
| positive regulation of interleukin-4 production (GO:0032753) | 0.001 | 3.30 | -1.94 | 14.76 | CD40LG;TNFSF4;CD28;NLRP3;GATA3 | |  | | |
| negative regulation of extrinsic apoptotic signaling pathway in absence of ligand (GO:2001240) | 0.001 | 3.29 | -1.83 | 13.81 | MAPK7;BCL2;HSPA1B;MCL1;PF4;HSPA1A | |  | | |
| negative regulation of signal transduction in absence of ligand (GO:1901099) | 0.001 | 3.29 | -1.82 | 13.76 | MAPK7;BCL2;HSPA1B;MCL1;PF4;HSPA1A | |  | | |
| lamellipodium assembly (GO:0030032) | 0.001 | 3.29 | -1.76 | 13.30 | CCDC88A;ABLIM1;ABLIM3;S1PR1;PARVB;VCL | |  | | |
| regulation of erythrocyte differentiation (GO:0045646) | 0.001 | 3.28 | -1.36 | 10.27 | HSPA9;MAFB;TAL1;HIF1A;HSPA1B;ZFP36L1;HSPA1A | |  | | |
| response to lipid (GO:0033993) | 0.001 | 3.26 | -1.34 | 10.03 | CDKN2D;JUN;F2R;PTPN22;PPBP;NR1D1;AQP3;CXCL5;SELP;CD6;CD27;CTNNB1;TNFRSF25;PF4V1;PF4;SNCA | |  | | |
| positive regulation of cell adhesion (GO:0045785) | 0.001 | 3.25 | -1.80 | 13.44 | CCL5;TPM1;SIRPG;ADAM9;PTPRJ;CCR7;RSU1;LIMS1;VEGFA;SMAD7 | |  | | |
| positive regulation of protein kinase B signaling (GO:0051897) | 0.001 | 3.24 | -1.16 | 8.66 | ITGB1;OSBPL8;ANGPT1;EGF;TRAT1;PDGFA;PTPRJ;GATA3;THBS1;PTK2;CD28;CCL3;CCR7;ICOS;HBEGF | |  | | |
| negative regulation of apoptotic signaling pathway (GO:2001234) | 0.001 | 3.19 | -2.06 | 15.17 | URI1;GSTP1;BCL2;PLAUR;PPIF;ITGA6;THBS1;CLU;MCL1;HSPA1A | |  | | |
| positive regulation of macromolecule biosynthetic process (GO:0010557) | 0.001 | 3.19 | -1.41 | 10.32 | MTPN;CIITA;DHX9;EGF;F2R;PDGFA;KLF4;THBS1;AREG;HIF1A;FCER2;HSPH1;MYC;BCL3;NAMPT;CD28 | |  | | |
| chaperone mediated protein folding requiring cofactor (GO:0051085) | 0.001 | 3.18 | -1.85 | 13.56 | HSPA9;HSPH1;HSPA5;HYOU1;HSPA1B;HSPA1A | |  | | |
| regulation of interferon-gamma secretion (GO:1902713) | 0.001 | 3.18 | -2.65 | 19.38 | CD2;ZC3H12A;PTPN22;RASGRP1 | |  | | |
| regulation of transcription from RNA polymerase II promoter (GO:0006357) | 0.001 | 3.17 | -1.28 | 9.37 | CSRNP1;APP;CDKN1A;CCNK;CIITA;CCNH;PRDM1;AHR;SLA2;PCSK6;CD3D;IKZF3;MED18;ETS2;IRF2BPL;SPIB;RPS6KA5;KAT5;MYC;ZC3H12A;NAMPT;EP300;IKBKG;ZNF367;NCK1;KDM6B;NCOA1;KLF11;KLF12;ZHX2;RIPK2;ARID5B;SUPT3H;THAP1;HCFC1;ZEB1;DMTF1;TAL1;ITGA6;ANKRA2;ATF3;URI1;DHX9;MAX;LEF1;NOP2;TCF7;GATA3;HIF1A;FSTL1;MAPK7;NRIP1;NLRP3;SKIL;SNCA;PLK3;CBX7;EGR1;JUN;ZFHX3;CBX5;CBX4;BCL11B;PCGF5;OSM;NR1D1;KLF4;SOD2;KLF2;BMP6;FOSL2;VEGFA;SMAD7;FOSL1;NR4A1;PER1;GFI1B;NR4A3;BCL6;POU2AF1;BHLHE40;SNAI1;FOSB;CTNNB1;TAF7;ZNF217;MAFK;QRICH1;MXD1;NFKBID;GZF1;PF4 | |  | | |
| dendritic cell chemotaxis (GO:0002407) | 0.001 | 3.16 | -2.27 | 16.57 | GPR183;CCL5;CXCR4;CCR7;CCR6 | |  | | |
| positive regulation of cell proliferation (GO:0008284) | 0.001 | 3.12 | -1.46 | 10.51 | CDKN1A;DHX9;LEF1;NOP2;RTKN2;SIRPG;PDGFA;THBS1;AREG;CXCL5;DPP4;TCL1A;RPS15A;GRK5;CCL5;MYC;NAMPT;ZNF703;RBPMS2;EGF;F2R;OSM;FLT3LG;BMP6;PTK2;CDC25B;VEGFA;FOSL1;NR4A3;TNFSF4;ID2;IL7R;EIF4G1;HBEGF | |  | | |
| regulation of wound healing (GO:0061041) | 0.001 | 3.12 | -1.81 | 12.98 | VIL1;TNFRSF12A;F2R;CASK;TNFAIP3;GP1BA;HBEGF | |  | | |
| cellular response to lipopolysaccharide (GO:0071222) | 0.001 | 3.11 | -1.69 | 12.13 | CD6;TNFSF4;CCL5;GSTP1;ZC3H12A;PDE4B;CCL3;ADAM9;TNFAIP3;NLRP3;PTPN22;NR1D1 | |  | | |
| receptor-mediated endocytosis (GO:0006898) | 0.001 | 3.09 | -1.32 | 9.41 | ITGB1;LMBR1L;SPARC;TFRC;HIP1R;HBB;ADM;ARRB2;ADRB2;HSP90B1;CXCL16;DNM3;HSPH1;RAB31;CTTN;CD9;HYOU1;LDLR;SNCA | |  | | |
| negative regulation of nucleic acid-templated transcription (GO:1903507) | 0.001 | 3.07 | -1.17 | 8.24 | CREBZF;ZNF253;CIITA;LEF1;PBXIP1;GATA3;AHR;RPS6KA5;KAT5;ZNF703;CD38;JARID2;JUN;ZHX2;CBX5;CBX4;ARID5B;NR1D1;KLF4;PER1;DAB2;ZEB1;BCL6;TNFSF4;ID2;BCL3;ID1;BHLHE40;ZNF318;CTNNB1;TAF7;ZNF217;MPHOSPH8;GZF1;LIMS1 | |  | | |
| positive regulation of intracellular signal transduction (GO:1902533) | 0.001 | 3.05 | -1.48 | 10.40 | ITGB1;APP;PDGFA;PTPRJ;GATA3;ADRB2;CBL;ATP2C1;THBS1;CCL5;CCL3;FLNA;PMAIP1;CCR7;IKBKG;PIM2;ICOS;OSBPL8;ANGPT1;EGF;RIPK2;TRAT1;F2R;OSM;PTK2;VEGFA;SELP;P2RX5;TRAF5;CD28;BCL2;CD27;TNFRSF25;IL7R;PF4;LIMS1;HBEGF | |  | | |
| ERBB signaling pathway (GO:0038127) | 0.001 | 3.04 | -1.43 | 10.01 | VIL1;PTPN18;NRAS;RPS6KA5;EGF;PLCG1;IQGAP1;CBL;AREG;PTK2;HBEGF | |  | | |
| regulation of endothelial cell apoptotic process (GO:2000351) | 0.001 | 3.04 | -1.77 | 12.39 | CD40LG;MAPK7;ANGPT1;TNFAIP3;ANO6;GATA3;THBS1 | |  | | |
| cellular response to organic substance (GO:0071310) | 0.001 | 3.03 | -1.08 | 7.53 | NCOA1;PTPN18;JUN;URI1;LEF1;CXCR4;KLF4;MAPK13;MAPK7;CCL5;CCL3;CTNNB1;SIK1;CCR7;MAPK6 | |  | | |
| positive regulation of cell death (GO:0010942) | 0.001 | 3.00 | -1.92 | 13.27 | EGR1;RIPK2;ZC3H12A;DDIT4;HBB;AXIN2;CLU;SNCA;EIF4G1 | |  | | |
| regulation of DNA-templated transcription, initiation (GO:2000142) | 0.001 | 3.00 | -2.54 | 17.52 | FOSL1;JUN;CTNNB1;TAF7 | |  | | |
| response to laminar fluid shear stress (GO:0034616) | 0.001 | 3.00 | -2.46 | 17.00 | MAPK7;KLF4;KLF2;SMAD7 | |  | | |
| regulation of T cell receptor signaling pathway (GO:0050856) | 0.001 | 2.99 | -1.93 | 13.28 | TESPA1;TRAT1;PTPRJ;PTPN22;CD226;UBASH3A | |  | | |
| regulation of transcription, DNA-templated (GO:0006355) | 0.001 | 2.96 | -1.67 | 11.41 | ZNF573;NAB2;AHR;IKZF3;BACH2;ETS2;SPIB;RPS6KA5;KAT5;MYC;EP300;PIM2;ZNF322;F2R;ARID5B;THAP1;HCFC1;DMTF1;ZNF318;ANKRA2;ATF3;NOTCH2;URI1;DHX9;NOP2;TCF7;GATA3;NLK;HIF1A;ZNF703;ZNF782;EGR1;JUN;ZFHX3;CBX5;CBX4;BCL11B;NR1D1;FOSL2;FOSL1;DAB2;MAFB;BCL6;TNFSF4;ID2;BCL3;ID1;BHLHE40;SNAI1;SP140L;NFKBID;CREBZF;ZNF253;CDKN1A;CIITA;ZNF493;TGFB1I1;MED18;CD38;JARID2;ZNF367;YWHAH;NCOA1;KLF11;KLF12;ZHX2;RHOH;SUPT3H;ZEB1;TAL1;ZNF780B;ZNF780A;LEF1;PBXIP1;SPTY2D1;IWS1;SERTAD3;TP53INP2;NRIP1;KLRG1;ZNF585A;EGF;KLF4;SOD2;KLF2;VEGFA;ZNF33A;PER1;CAMK4;FOSB;CTNNB1;TAF7;ZNF217;QRICH1;MPHOSPH8;GZF1;LIMS1 | |  | | |
| positive regulation of gene expression (GO:0010628) | 0.001 | 2.91 | -1.54 | 10.32 | APP;CIITA;TGFB1I1;PTPN22;AHR;CD3E;CLU;ETS2;KAT5;MYC;ZC3H12A;CD38;EP300;PIM2;YWHAH;NCOA1;NAT8B;F2R;SUPT3H;HCFC1;TAL1;ZNF318;LEF1;DDX21;GATA3;THBS1;HIF1A;SERTAD3;HIST1H3A;TP53INP2;NRIP1;CCL3;EGR1;JUN;ZFHX3;EGF;EPHX2;NR1D1;KLF4;KLF2;VEGFA;HIST1H4A;DAB2;ID2;CAMK4;BCL3;CD28;SNAI1;CTNNB1;HSPA1B;PF4;LIMS1;HSPA1A | |  | | |
| regulation of cell death (GO:0010941) | 0.001 | 2.91 | -1.30 | 8.68 | JUN;RIPK2;CCL5;ZC3H12A;CTNNB1;HBB;AXIN2;CLU;HSPA1B;NCK1;EIF4G1;HSPA1A | |  | | |
| cellular response to topologically incorrect protein (GO:0035967) | 0.001 | 2.90 | -1.98 | 13.25 | HSPA9;HSPH1;HSPA5;HYOU1;HSPA1B;HSPA1A | |  | | |
| leukocyte cell-cell adhesion (GO:0007159) | 0.001 | 2.90 | -1.97 | 13.18 | SELP;ITGB1;NT5E;CD40LG;CCL5;FERMT3 | |  | | |
| regulation of extrinsic apoptotic signaling pathway in absence of ligand (GO:2001239) | 0.001 | 2.90 | -1.81 | 12.12 | MAPK7;BCL2;HSPA1B;MCL1;PF4;HSPA1A | |  | | |
| phosphatidylserine metabolic process (GO:0006658) | 0.001 | 2.90 | -1.40 | 9.38 | OSBPL8;PLA2G12A;LPCAT4;PLA2G4B;LPCAT3;OSBPL10 | |  | | |
| cell junction assembly (GO:0034329) | 0.001 | 2.90 | -1.33 | 8.85 | VMP1;ZNF703;CTNNB1;FLNA;ITGA6;TLN1;VCL;LIMS1;SMAD7 | |  | | |
| regulation of peptidyl-threonine phosphorylation (GO:0010799) | 0.001 | 2.89 | -1.56 | 10.41 | APP;EGF;RIPK2;DDIT4;CALM3;EIF4G1;SMAD7 | |  | | |
| protein deubiquitination (GO:0016579) | 0.001 | 2.89 | -1.65 | 10.99 | BARD1;PSMD14;RIPK2;USP12;TNFAIP3;GATA3;AXIN2;ARRB2;ADRB2;JOSD1;HIF1A;PSMA7;SUPT3H;HCFC1;SMAD7;PSMC6;PSMD7;MYC;ZC3H12A;NLRP3;EP300;IKBKG;OTUD1 | |  | | |
| viral gene expression (GO:0019080) | 0.001 | 2.88 | -1.49 | 9.90 | RPL30;RPL32;RPL36A;FURIN;RPL7;RPS25;RPS27;RPS15A;RPL27A;NUP50;RPS20;RPS21;RPS13 | |  | | |
| positive regulation of apoptotic process (GO:0043065) | 0.001 | 2.85 | -1.56 | 10.21 | ITGB1;TNFAIP8;GNA13;NCSTN;PNMA1;MYC;ZC3H12A;CCL3;NLRP3;PMAIP1;SNCA;BARD1;KLF11;JUN;ARHGEF12;TNFRSF12A;F2R;HIP1R;ARHGEF18;ANO6;GZMK;OBSCN;BCL6;CTNNB1;SIK1;ITGA6 | |  | | |
| response to metal ion (GO:0010038) | 0.001 | 2.85 | -1.24 | 8.11 | SLC11A2;BCL2;ADAM9;SLC30A1;CALM3;THBS1;AQP3;HIF1A;SEC31A;SNCA | |  | | |
| cellular response to growth factor stimulus (GO:0071363) | 0.001 | 2.83 | -1.34 | 8.77 | URI1;MTSS1L;IQGAP1;KLF4;BMP6;ZFP36L1;VEGFA;SMAD7;VIL1;NR4A1;MAPK7;CCL5;CTNNB1;PLCG1;LIMS1 | |  | | |
| positive regulation of phosphate metabolic process (GO:0045937) | 0.002 | 2.82 | -1.89 | 12.25 | APP;EGF;CCL5;ITGA6;THBS1;SNCA | |  | | |
| negative regulation of homeostatic process (GO:0032845) | 0.002 | 2.82 | -1.53 | 9.93 | HSPA9;MAFB;BCL2;TNFAIP3;PIM3;ZFP36L1 | |  | | |
| protein modification by small protein removal (GO:0070646) | 0.002 | 2.80 | -1.15 | 7.40 | BARD1;USPL1;PSMD14;RIPK2;USP12;COPS7B;TNFAIP3;GATA3;AXIN2;ARRB2;ADRB2;JOSD1;HIF1A;PSMA7;HCFC1;SMAD7;PSMC6;PSMD7;MYC;ZC3H12A;NLRP3;EP300;IKBKG | |  | | |
| negative regulation of cellular macromolecule biosynthetic process (GO:2000113) | 0.002 | 2.80 | -0.99 | 6.36 | CREBZF;ZNF253;BTG2;CIITA;LEF1;PBXIP1;GATA3;AHR;RPS6KA5;KAT5;ZNF703;CD38;JARID2;JUN;ZHX2;CBX5;CBX4;EIF2AK1;ARID5B;NR1D1;KLF4;MEX3C;PER1;DAB2;ZEB1;BCL6;TNFSF4;ID2;BCL3;ID1;BHLHE40;ZNF318;CTNNB1;TAF7;ZNF217;MPHOSPH8;GZF1;LIMS1 | |  | | |
| B cell activation (GO:0042113) | 0.002 | 2.80 | -1.45 | 9.35 | FCRL1;ITGB1;CD79B;CD79A;CD40LG;GPR183;LEF1;BCL2;LAX1;MS4A1 | |  | | |
| actin cytoskeleton reorganization (GO:0031532) | 0.002 | 2.79 | -1.55 | 9.99 | CTTN;PLEK;S1PR1;RHOH;FLNA;NEDD9;MYH9;PARVB;ATP2C1 | |  | | |
| regulation of tumor necrosis factor production (GO:0032680) | 0.002 | 2.79 | -1.31 | 8.43 | CD2;GSTP1;ZC3H12A;CCL3;TNFAIP3;PTPN22;RASGRP1;CLU;PF4 | |  | | |
| negative regulation of MAP kinase activity (GO:0043407) | 0.002 | 2.79 | -1.24 | 7.98 | DUSP2;DUSP10;SMPD1;GSTP1;STK38;PTPRJ;PTPN22;LAX1;DUSP16 | |  | | |
| viral transcription (GO:0019083) | 0.002 | 2.77 | -1.66 | 10.61 | RPL30;RPL32;DHX9;RPL36A;RPL7;RPS25;RPS27;RPS15A;RPL27A;NUP50;RPS20;RPS21;RPS13 | |  | | |
| receptor internalization (GO:0031623) | 0.002 | 2.76 | -1.36 | 8.64 | DNM3;ITGB1;RAB31;TFRC;CD9;ADM;ARRB2;SNCA | |  | | |
| positive regulation of transcription from RNA polymerase II promoter in response to stress (GO:0036003) | 0.002 | 2.74 | -1.76 | 11.12 | MAPK7;HSPA5;HIF1A;ATF3;KLF2;VEGFA | |  | | |
| regulation of neutrophil chemotaxis (GO:0090022) | 0.002 | 2.74 | -1.71 | 10.78 | CCR7;PPBP;CXCL5;PF4V1;PF4;JAM3 | |  | | |
| negative regulation of cellular process (GO:0048523) | 0.002 | 2.74 | -1.27 | 7.99 | APP;CDKN1A;BTG2;TGFB1I1;PDE3B;SIRPG;PTPRJ;GATA3;CLU;SERTAD3;SERTAD2;MYC;FTH1;LMNA;PIM2;SKIL;NCK1;CDKN2D;KLF11;ANGPT1;ACTN1;F2R;OSM;CASK;AXIN2;KLF4;SOD2;NR4A1;BCL6;ABI1;BHLHE40;BCL2;CD9;CTNNB1;MYH9;HSPA1B;APOBEC3A;PF4;HSPA1A | |  | | |
| entrainment of circadian clock by photoperiod (GO:0043153) | 0.002 | 2.71 | -2.25 | 14.04 | PPP1CB;PER1;ID2;BHLHE40;SIK1 | |  | | |
| mesoderm formation (GO:0001707) | 0.002 | 2.71 | -2.16 | 13.47 | KDM6B;ITGB1;ITGB3;LEF1;SNAI1 | |  | | |
| regulation of anoikis (GO:2000209) | 0.002 | 2.71 | -2.09 | 13.06 | ITGB1;BCL2;SIK1;PTK2;MCL1 | |  | | |
| regulation of macromolecule metabolic process (GO:0060255) | 0.002 | 2.70 | -1.19 | 7.42 | APP;ARF1;AHR;NLK;HIF1A;ZFP36L1;MAPK13;MFAP4;MAPK7;MYC;ZC3H12A;NLRP3;MAPK6;LDLR | |  | | |
| regulation of GTPase activity (GO:0043087) | 0.002 | 2.70 | -1.14 | 7.09 | ITGB1;CCL20;DOCK9;F2R;STMN3;F11R;RASGRP1;PTK2;RGP1;ARHGAP21;RGS1;CCL5;RGS10;CCL3;ITGA6;RSU1;RGS6;LIMS1 | |  | | |
| cellular response to arsenic-containing substance (GO:0071243) | 0.002 | 2.69 | -2.57 | 15.96 | ZC3H12A;PPIF;ATF3;MAPK13 | |  | | |
| regulation of antigen receptor-mediated signaling pathway (GO:0050854) | 0.002 | 2.69 | -1.69 | 10.50 | BLK;PTPN22;PAX5;GCSAM | |  | | |
| positive regulation of epithelial to mesenchymal transition (GO:0010718) | 0.002 | 2.69 | -1.97 | 12.20 | DAB2;TGFB1I1;LEF1;ZNF703;SNAI1;CTNNB1;AXIN2 | |  | | |
| transcription from RNA polymerase II promoter (GO:0006366) | 0.002 | 2.68 | -1.10 | 6.78 | NOTCH2;CDKN1A;CCNK;MAX;CCNH;TGFB1I1;LEF1;DDX21;GATA3;ARRB2;AHR;HIF1A;BACH2;MED18;IWS1;NR3C2;PCF11;SNAPC3;KLF11;EGR1;ZFHX3;TSC22D1;EAF1;PAX5;NR1D1;KLF4;HCFC1;FOSL1;NR4A1;GFI1B;NR4A3;POU2AF1;TRIP11;TAF7;NABP1;SRSF7 | |  | | |
| negative regulation of extrinsic apoptotic signaling pathway (GO:2001237) | 0.002 | 2.67 | -1.14 | 6.97 | MAPK7;GSTP1;BCL2;TNFAIP3;ITGA6;THBS1;HSPA1B;MCL1;PF4;HSPA1A | |  | | |
| SRP-dependent cotranslational protein targeting to membrane (GO:0006614) | 0.002 | 2.67 | -1.70 | 10.41 | RPS25;RPL30;RPS27;RPS15A;RPL32;RPL27A;RPL36A;RPS20;RPS21;RPS13;RPL7 | |  | | |
| 'de novo' posttranslational protein folding (GO:0051084) | 0.002 | 2.66 | -1.56 | 9.54 | HSPA9;HSPH1;HSPA5;HYOU1;HSPA1B;HSPA1A | |  | | |
| regulation of DNA binding (GO:0051101) | 0.002 | 2.65 | -1.24 | 7.55 | JUN;EGF;SUMO3;ID2;BCL3;ID1;LEF1;PLAUR | |  | | |
| cellular response to decreased oxygen levels (GO:0036294) | 0.002 | 2.65 | -1.33 | 8.09 | MYC;LMNA;PMAIP1;HYOU1;PTGS2;AQP3;HIF1A;ZFP36L1;VEGFA | |  | | |
| photoperiodism (GO:0009648) | 0.002 | 2.62 | -2.54 | 15.32 | PPP1CB;PER1;ID2;BHLHE40;SIK1 | |  | | |
| Ras protein signal transduction (GO:0007265) | 0.003 | 2.60 | -1.22 | 7.29 | CDKN1A;JUN;RHOH;RAB27B;ARHGAP6;RAB11A;RHOBTB1;RAB32;RAP1B;GNA13;RIT1;NRAS;RAB20;RAP2B;RAB31;RAB37;GRAP2;RHOU;GRAP;RAB6B | |  | | |
| positive regulation of transcription, DNA-templated (GO:0045893) | 0.003 | 2.57 | -1.50 | 8.86 | CSRNP1;APP;CCNK;CIITA;CCNH;TGFB1I1;AHR;CD3D;IKZF3;ETS2;IRF2BPL;SPIB;RPS6KA5;KAT5;MYC;ZC3H12A;NAMPT;CD38;EP300;IKBKG;PIM2;YWHAH;NCK1;KDM6B;NCOA1;RIPK2;F2R;SUPT3H;HCFC1;TAL1;ZNF318;ITGA6;ASF1A;ATF3;DHX9;LEF1;GATA3;HIF1A;SERTAD3;MAPK7;TP53INP2;NRIP1;NLRP3;EGR1;JUN;ZFHX3;BCL11B;EGF;PCGF5;OSM;NR1D1;KLF4;KLF2;BMP6;FOSL2;VEGFA;SMAD7;FOSL1;NR4A1;PER1;DAB2;NR4A3;POU2AF1;ID2;CAMK4;BCL3;SNAI1;CTNNB1;TAF7;PF4 | |  | | |
| oxygen homeostasis (GO:0032364) | 0.003 | 2.57 | -4.53 | 26.82 | ALAS2;SOD2;HIF1A | |  | | |
| positive regulation of hormone metabolic process (GO:0032352) | 0.003 | 2.57 | -3.12 | 18.48 | EGR1;GATA3;HIF1A | |  | | |
| negative regulation of erythrocyte differentiation (GO:0045647) | 0.003 | 2.57 | -3.06 | 18.12 | HSPA9;MAFB;ZFP36L1 | |  | | |
| positive regulation of extrinsic apoptotic signaling pathway via death domain receptors (GO:1902043) | 0.003 | 2.57 | -3.05 | 18.07 | PMAIP1;THBS1;ATF3 | |  | | |
| positive regulation of interferon-gamma secretion (GO:1902715) | 0.003 | 2.57 | -2.91 | 17.19 | CD2;PTPN22;RASGRP1 | |  | | |
| activation of transmembrane receptor protein tyrosine kinase activity (GO:0007171) | 0.003 | 2.57 | -2.62 | 15.51 | ANGPT1;PILRB;ADRB2 | |  | | |
| regulation of protein kinase B signaling (GO:0051896) | 0.003 | 2.56 | -1.16 | 6.84 | ITGB1;OSBPL8;ANGPT1;EGF;TRAT1;PDGFA;PTPRJ;GATA3;ARRB2;THBS1;PTK2;CD28;CCL3;CCR7;ICOS;HBEGF | |  | | |
| enzyme linked receptor protein signaling pathway (GO:0007167) | 0.003 | 2.54 | -1.59 | 9.30 | BLK;ITK;NEDD9;PILRB;SLA2;GP6;CD3E;PTK2;CD8B;CD8A;GRAP2;ABI1;GRAP | |  | | |
| positive regulation of T cell differentiation (GO:0045582) | 0.003 | 2.52 | -1.64 | 9.53 | DUSP10;PNP;BCL6;TESPA1;TNFSF4;GATA3 | |  | | |
| regulation of cell-cell adhesion (GO:0022407) | 0.003 | 2.52 | -1.59 | 9.24 | DPP4;LEF1;SIRPG;PTK2;SMAD7;FERMT3 | |  | | |
| regulation of nitric oxide biosynthetic process (GO:0045428) | 0.003 | 2.52 | -1.42 | 8.22 | ZC3H12A;HBB;PTGS2;KLF4;CLU;KLF2 | |  | | |
| cellular transition metal ion homeostasis (GO:0046916) | 0.003 | 2.52 | -1.46 | 8.45 | ATP6V1A;APP;ARF1;ALAS2;TFRC;MYC;FTH1;SLC11A2;SLC40A1;SLC30A1;BMP6 | |  | | |
| cotranslational protein targeting to membrane (GO:0006613) | 0.003 | 2.52 | -1.28 | 7.40 | RPS25;RPL30;RPS27;RPS15A;RPL32;RPL27A;RPL36A;RPS20;RPS21;RPS13;RPL7 | |  | | |
| regulation of cysteine-type endopeptidase activity involved in apoptotic process (GO:0043281) | 0.003 | 2.52 | -1.23 | 7.14 | CDKN2D;DNAJB6;MYC;LEF1;F2R;NLRP3;PMAIP1;KLF4;THBS1;SNCA;VEGFA | |  | | |
| response to hydrogen peroxide (GO:0042542) | 0.003 | 2.51 | -1.53 | 8.82 | MAPK7;PPIF;ADAM9;TNFAIP3;HBB;ANKZF1;MAPK13 | |  | | |
| Notch signaling pathway (GO:0007219) | 0.003 | 2.49 | -1.74 | 9.98 | NOTCH2;FCER2;NCSTN;MYC;SNAI1;EP300;NRARP;PBX1 | |  | | |
| regulation of inflammatory response (GO:0050727) | 0.003 | 2.48 | -0.93 | 5.30 | DHX9;TNFAIP3;GATA3;PTGS2;KLF4;MAPK13;PER1;MAPK7;BCL6;TNFSF4;CCL5;CCL3;NLRP3;DNASE1;MGLL;SNCA | |  | | |
| positive regulation of protein phosphorylation (GO:0001934) | 0.003 | 2.47 | -1.64 | 9.37 | APP;CDKN1A;CCNK;CCNH;ITGB3;PDGFA;IQGAP1;ADRB2;RASGRP1;TCL1A;RPS6KA5;CCR7;SNCA;ANGPT1;EGF;RIPK2;MMD;F2R;PLAUR;OSM;AXIN2;BMP6;PTK2;VEGFA;DAB2;TWSG1;CDK2AP1;CD27;TNFRSF25;CALM3;EIF4G1 | |  | | |
| regulation of gene expression (GO:0010468) | 0.004 | 2.46 | -1.62 | 9.15 | ZNF573;APP;ZNF253;ZNF493;NAB2;PTPN22;AHR;CD3E;CLU;BACH2;RPS6KA5;MYC;ZC3H12A;EP300;ZNF322;NAT8B;RHOH;SUPT3H;THAP1;HCFC1;DMTF1;MATR3;ZNF780B;ZNF780A;NOTCH2;DHX9;LEF1;TCF7;SPTY2D1;NLK;HIF1A;IWS1;ZFP36L1;SERTAD3;MAPK7;HIST1H3A;ZNF703;CCL3;NLRP3;MAPK6;KLRG1;ZNF585A;ZNF782;EGR1;ZFHX3;PTCD2;BCL11B;EGF;EPHX2;KLF4;CLK2;MAPK13;VEGFA;ZNF33A;PER1;MAFB;ID2;BHLHE40;CD28;ZNF217;SP140L;HSPA1B;PF4;LIMS1;HSPA1A | |  | | |
| regulation of focal adhesion assembly (GO:0051893) | 0.004 | 2.45 | -1.78 | 10.05 | PTPRJ;THBS1;ARHGAP6;PTK2;CORO1C;LIMS1;VEGFA | |  | | |
| sprouting angiogenesis (GO:0002040) | 0.004 | 2.45 | -1.66 | 9.40 | NR4A1;ANGPT1;LEF1;NRARP;THBS1;JMJD6;VEGFA | |  | | |
| regulation of smooth muscle cell proliferation (GO:0048660) | 0.004 | 2.45 | -1.51 | 8.55 | NR4A3;CCL5;ID2;RBPMS2;CTNNB1;TNFAIP3;THBS1 | |  | | |
| cellular iron ion homeostasis (GO:0006879) | 0.004 | 2.45 | -2.35 | 13.22 | ATP6V1A;ALAS2;TFRC;MYC;FTH1;SLC11A2;SLC40A1;BMP6 | |  | | |
| branching involved in blood vessel morphogenesis (GO:0001569) | 0.004 | 2.44 | -2.13 | 11.98 | LEF1;CTNNB1;NRARP;VEGFA | |  | | |
| regulation of lymphocyte differentiation (GO:0045619) | 0.004 | 2.44 | -2.11 | 11.89 | CD2;PRDM1;IKZF3;ZFP36L1 | |  | | |
| apoptotic process (GO:0006915) | 0.004 | 2.42 | -1.78 | 9.90 | NOTCH2;PLK3;CSRNP1;APP;EGLN3;CXCR4;NSG1;AHR;CD2;IL2RA;MYC;BCL2;PPIF;MAL;NLRP3;EP300;PMAIP1;TNFRSF25;IKBKG;PHLDA2 | |  | | |
| negative regulation of macromolecule metabolic process (GO:0010605) | 0.004 | 2.42 | -1.56 | 8.69 | NOTCH2;APP;TRAT1;PTPN22;KLF4;CD3E;HIF1A;VEGFA;ID2;ZC3H12A;CD28;CCL3;LDLR | |  | | |
| IRE1-mediated unfolded protein response (GO:0036498) | 0.004 | 2.40 | -1.34 | 7.38 | HSPA5;PLA2G4B;LMNA;DNAJB11;TPP1;HYOU1;TLN1;SEC31A | |  | | |
| positive regulation of endothelial cell migration (GO:0010595) | 0.004 | 2.38 | -1.46 | 8.00 | SPARC;ANGPT1;ITGB3;ZC3H12A;PLCG1;GATA3;THBS1;HIF1A;VEGFA | |  | | |
| gas homeostasis (GO:0033483) | 0.004 | 2.38 | -3.99 | 21.87 | ALAS2;SOD2;HIF1A | |  | | |
| cellular response to laminar fluid shear stress (GO:0071499) | 0.004 | 2.38 | -2.73 | 14.96 | MAPK7;KLF4;KLF2 | |  | | |
| regulation of cyclic nucleotide metabolic process (GO:0030799) | 0.004 | 2.38 | -2.70 | 14.78 | CALM3;THBS1;VEGFA | |  | | |
| regulation of cell-cell adhesion mediated by integrin (GO:0033632) | 0.004 | 2.38 | -1.73 | 9.47 | DPP4;CCL5;FERMT3 | |  | | |
| protein targeting to ER (GO:0045047) | 0.004 | 2.37 | -1.83 | 10.03 | RPS25;RPL30;RPS27;RPS15A;RPL32;RPL27A;RPL36A;RPS20;RPS21;RPS13;RPL7 | |  | | |
| positive regulation of neutrophil chemotaxis (GO:0090023) | 0.004 | 2.36 | -2.02 | 10.98 | CCR7;PPBP;CXCL5;PF4V1;PF4 | |  | | |
| negative regulation of autophagy (GO:0010507) | 0.005 | 2.35 | -1.78 | 9.59 | CTSA;BCL2;PTPN22;EIF4G2;MCL1;SNCA;EIF4G1 | |  | | |
| regulation of fibroblast proliferation (GO:0048145) | 0.005 | 2.35 | -1.48 | 7.99 | CDKN1A;DHX9;MYC;GSTP1;FTH1;PDGFA;CTNNB1 | |  | | |
| regulation of immune response (GO:0050776) | 0.005 | 2.34 | -1.13 | 6.11 | ITGB1;CD96;BTN3A1;PTPN22;CD3G;PILRB;SLA2;CD3E;CD3D;TREM1;TREML1;CD40LG;BCL6;CD8B;CD8A;TNFSF4;NLRP3;SLAMF6;CD226;CLEC2D;ICOSLG | |  | | |
| nuclear-transcribed mRNA catabolic process, nonsense-mediated decay (GO:0000184) | 0.005 | 2.34 | -1.78 | 9.60 | RPS25;RPL30;RPS27;RPS15A;RPL32;RPL27A;RPL36A;RPS20;RPS21;RPS13;RPL7;EIF4G1 | |  | | |
| negative regulation of peptidyl-threonine phosphorylation (GO:0010801) | 0.005 | 2.33 | -2.46 | 13.20 | DDIT4;CALM3;EIF4G1;SMAD7 | |  | | |
| regulation of B cell differentiation (GO:0045577) | 0.005 | 2.33 | -2.21 | 11.89 | MMP14;CD27;IKZF3;ZFP36L1 | |  | | |
| negative regulation of platelet activation (GO:0010544) | 0.005 | 2.33 | -2.06 | 11.08 | THBD;PDGFA;CD9;ALOX12 | |  | | |
| negative regulation of anoikis (GO:2000811) | 0.005 | 2.33 | -2.00 | 10.72 | ITGB1;BCL2;PTK2;MCL1 | |  | | |
| protein kinase B signaling (GO:0043491) | 0.005 | 2.32 | -1.77 | 9.44 | NR4A1;PLK3;CDKN1A;CCL5;CCL3;ZFP36L1 | |  | | |
| viral process (GO:0016032) | 0.005 | 2.32 | -1.48 | 7.89 | ARF1;RPL30;RPL32;RPL36A;FURIN;RPL7;HCFC1;RPS25;RPS27;RPS15A;CD8B;RPL27A;NUP50;CD28;RPS20;DOCK2;RPS21;CHMP7;RPS13 | |  | | |
| positive regulation of NF-kappaB transcription factor activity (GO:0051092) | 0.01 | 2.30 | -1.14 | 6.06 | MTPN;APP;DHX9;RIPK2;CLU;RPS6KA5;CD40LG;TRAF5;NLRP3;IKBKG;HSPA1B;NFKBIB;HSPA1A | |  | | |
| cellular response to heat (GO:0034605) | 0.01 | 2.29 | -2.03 | 10.74 | HSPA9;HSPH1;HSPA5;HYOU1;THBS1;HSPA1B;HSPA1A | |  | | |
| positive regulation of tumor necrosis factor production (GO:0032760) | 0.01 | 2.29 | -1.60 | 8.43 | CD2;DHX9;CCL3;RASGRP1;THBS1;CLU;PF4 | |  | | |
| positive regulation of interleukin-12 production (GO:0032735) | 0.01 | 2.28 | -2.04 | 10.71 | CD40LG;RIPK2;TNFSF4;CCR7;LTB | |  | | |
| positive regulation of nitric oxide biosynthetic process (GO:0045429) | 0.01 | 2.28 | -1.94 | 10.22 | HBB;PTGS2;KLF4;CLU;KLF2 | |  | | |
| positive regulation of nitric oxide metabolic process (GO:1904407) | 0.01 | 2.28 | -1.87 | 9.82 | HBB;PTGS2;KLF4;CLU;KLF2 | |  | | |
| positive regulation of neutrophil migration (GO:1902624) | 0.01 | 2.28 | -1.84 | 9.64 | CCR7;PPBP;CXCL5;PF4V1;PF4 | |  | | |
| regulation of peptidyl-serine phosphorylation (GO:0033135) | 0.01 | 2.28 | -1.74 | 9.12 | APP;TCL1A;RIPK2;DDIT4;OSM;SNCA;NCK1;EIF4G1;VEGFA;SMAD7 | |  | | |
| response to reactive oxygen species (GO:0000302) | 0.01 | 2.26 | -1.18 | 6.14 | PLK3;JUN;GSTP1;TPM1;ADAM9;HBB;CCR7;SOD2 | |  | | |
| regulation of epithelial cell migration (GO:0010632) | 0.01 | 2.24 | -1.70 | 8.78 | VIL1;DUSP10;PLCG1;HIF1A;RAB11A;PTK2;CORO1C | |  | | |
| lipopolysaccharide-mediated signaling pathway (GO:0031663) | 0.01 | 2.23 | -2.11 | 10.84 | CD6;CCL5;CCL3;PTPN22 | |  | | |
| regulation of chaperone-mediated autophagy (GO:1904714) | 0.01 | 2.22 | -2.81 | 14.35 | CTSA;PLK3;SNCA | |  | | |
| regulation of sensory perception (GO:0051931) | 0.01 | 2.22 | -2.66 | 13.59 | F2R;CCL3;MGLL | |  | | |
| positive regulation of immunoglobulin mediated immune response (GO:0002891) | 0.01 | 2.22 | -2.54 | 12.99 | TFRC;TNFSF4;CD226 | |  | | |
| regulation of amyloid fibril formation (GO:1905906) | 0.01 | 2.22 | -1.68 | 8.60 | APP;LDLR;CLU | |  | | |
| positive regulation of protein complex assembly (GO:0031334) | 0.01 | 2.21 | -1.72 | 8.73 | FOSL1;JUN;TCL1A;TAL1;PLEK;CTNNB1;PMAIP1;EIF4G1;VEGFA;TRABD2A | |  | | |
| positive regulation of granulocyte chemotaxis (GO:0071624) | 0.01 | 2.21 | -1.82 | 9.27 | CCR7;PPBP;CXCL5;PF4V1;PF4 | |  | | |
| regulation of p38MAPK cascade (GO:1900744) | 0.01 | 2.21 | -1.61 | 8.19 | PER1;DUSP10;ZC3H12A;PTPN22;VEGFA | |  | | |
| phosphatidylethanolamine acyl-chain remodeling (GO:0036152) | 0.01 | 2.21 | -1.46 | 7.42 | PLA2G12A;ABHD4;LPCAT4;PLA2G4B;LPCAT3 | |  | | |
| response to growth factor (GO:0070848) | 0.01 | 2.21 | -1.44 | 7.33 | URI1;MAPK7;MYC;CTNNB1;KLF4 | |  | | |
| positive regulation of smooth muscle cell proliferation (GO:0048661) | 0.01 | 2.20 | -1.67 | 8.46 | JUN;NR4A3;CCL5;ID2;RBPMS2;THBS1 | |  | | |
| positive regulation of nucleic acid-templated transcription (GO:1903508) | 0.01 | 2.20 | -1.63 | 8.23 | CIITA;TGFB1I1;LEF1;GATA3;AHR;HIF1A;ETS2;SERTAD3;KAT5;MYC;TP53INP2;NRIP1;CD38;EP300;PIM2;YWHAH;NCOA1;EGR1;JUN;ZFHX3;EGF;F2R;NR1D1;KLF4;KLF2;SUPT3H;HCFC1;DAB2;TAL1;ID2;CAMK4;BCL3;ZNF318;SNAI1;CTNNB1 | |  | | |
| negative regulation of cell proliferation (GO:0008285) | 0.01 | 2.16 | -1.55 | 7.70 | APP;CDKN1A;BTG2;TGFB1I1;GSTP1;SIRPG;TNFAIP3;PTPRJ;GATA3;DUSP10;MYC;FTH1;LMNA;PIM2;CDKN2D;KLF11;F2R;OSM;AXIN2;KLF4;SOD2;ID2;ABI1;BHLHE40;CTNNB1;HSPA1B;HSPA1A | |  | | |
| positive regulation of neuron death (GO:1901216) | 0.01 | 2.15 | -1.84 | 9.10 | EGR1;DDIT4;CCL3;CTNNB1;CLU;SNCA | |  | | |
| regulation of B cell receptor signaling pathway (GO:0050855) | 0.01 | 2.14 | -2.40 | 11.81 | BLK;PTPN22;PAX5;GCSAM | |  | | |
| positive regulation of lymphocyte migration (GO:2000403) | 0.01 | 2.14 | -2.02 | 9.94 | APP;CCL20;CCL5;CCL3 | |  | | |
| regulation of T cell migration (GO:2000404) | 0.01 | 2.14 | -1.54 | 7.57 | APP;CCL20;CCL5;CCR6 | |  | | |
| modulation by host of viral transcription (GO:0043921) | 0.01 | 2.14 | -1.51 | 7.44 | JUN;CCL5;LEF1;CCL3;EP300 | |  | | |
| regulation of phosphorylation (GO:0042325) | 0.01 | 2.14 | -1.09 | 5.38 | BARD1;CDKN2D;CCDC88A;APP;CDKN1A;EGF;CCL5;NUP50;ITGA6;THBS1;PTK2;CORO1C | |  | | |
| iron ion homeostasis (GO:0055072) | 0.01 | 2.14 | -1.41 | 6.94 | ATP6V1A;ALAS2;TFRC;MYC;FTH1;SLC11A2;SLC40A1;BMP6 | |  | | |
| cellular response to interleukin-1 (GO:0071347) | 0.01 | 2.12 | -1.02 | 4.99 | EGR1;PSMD14;CCL20;RIPK2;HIF1A;PSMA7;MAPK13;RPS6KA5;PSMC6;PSMD7;CCL5;ZC3H12A;CCL3;IKBKG | |  | | |
| positive regulation of endocytosis (GO:0045807) | 0.01 | 2.10 | -1.15 | 5.54 | DAB2;SIRPG;HIP1R;CBL;DOCK2;LDLR;CLU;SNCA | |  | | |
| response to interleukin-4 (GO:0070670) | 0.01 | 2.08 | -2.63 | 12.56 | LEF1;TCF7;GATA3 | |  | | |
| positive regulation of cell activation (GO:0050867) | 0.01 | 2.08 | -2.53 | 12.10 | SELP;APP;PLEK | |  | | |
| regulation of sensory perception of pain (GO:0051930) | 0.01 | 2.08 | -2.44 | 11.66 | F2R;CCL3;MGLL | |  | | |
| mesodermal cell differentiation (GO:0048333) | 0.01 | 2.08 | -2.42 | 11.60 | KDM6B;ITGB1;ITGB3 | |  | | |
| cyclic nucleotide catabolic process (GO:0009214) | 0.01 | 2.08 | -2.10 | 10.04 | PDE3B;PDE4B;PDE5A | |  | | |
| cellular response to interleukin-4 (GO:0071353) | 0.01 | 2.08 | -1.77 | 8.46 | LEF1;TCF7;GATA3 | |  | | |
| regulation of transcription from RNA polymerase II promoter in response to hypoxia (GO:0061418) | 0.01 | 2.08 | -1.20 | 5.73 | EGLN3;PSMC6;PSMD7;PSMD14;UBE2D2;EP300;HIF1A;PSMA7;VEGFA | |  | | |
| cellular response to hydrogen peroxide (GO:0070301) | 0.01 | 2.07 | -1.90 | 9.09 | MAPK7;PPIF;TNFAIP3;ANKZF1;MAPK13 | |  | | |
| regulation of receptor internalization (GO:0002090) | 0.01 | 2.07 | -1.90 | 9.09 | ARF1;ANGPT1;EGF;ARRB2;VEGFA | |  | | |
| regulation of mRNA processing (GO:0050684) | 0.01 | 2.07 | -1.68 | 8.04 | PTCD2;DHX9;IWS1;JMJD6;ZFP36L1 | |  | | |
| negative regulation of signal transduction (GO:0009968) | 0.01 | 2.06 | -1.11 | 5.27 | PTPN18;LEPROT;CISH;EGF;PLEK;TNFAIP3;PTPRJ;NRARP;GATA3;NLK;THBS1;SOCS3;MMP14;SMPD1;CCL5;DDIT4;BCL2;UBASH3A;SNCA;NCK1;HBEGF;TRABD2A | |  | | |
| chemokine-mediated signaling pathway (GO:0070098) | 0.01 | 2.06 | -1.26 | 5.97 | CCL20;CCL5;CCL3;PPBP;CXCL5;PF4V1;PF4 | |  | | |
| blood vessel endothelial cell migration (GO:0043534) | 0.01 | 2.05 | -1.65 | 7.79 | NR4A1;ID1;MYH9;VEGFA | |  | | |
| regulation of cell growth (GO:0001558) | 0.01 | 2.04 | -1.00 | 4.69 | CDKN2D;MTPN;CDKN1A;URI1;CISH;LEF1;PTPRJ;CXCL16;SERTAD3;MMP14;BCL6;SERTAD2;BCL2;EIF4G2;HSPA1B;EIF4G1;HSPA1A | |  | | |
| negative regulation of transport (GO:0051051) | 0.01 | 2.04 | -1.51 | 7.09 | YRDC;MCTP1;EGF;TRAT1;ITGB3;SNCA | |  | | |
| phosphatidic acid biosynthetic process (GO:0006654) | 0.01 | 2.04 | -1.41 | 6.62 | PLA2G12A;LPCAT4;PLA2G4B;DGKA;LIPH;AGPAT1 | |  | | |
| regulation of cytokine production (GO:0001817) | 0.01 | 2.03 | -1.10 | 5.16 | PER1;TWSG1;RPS6KA5;BTN3A1;TNFSF4;NLRP3;CCR7;GATA3;AGPAT1;ICOSLG;TRIM56 | |  | | |
| tumor necrosis factor-mediated signaling pathway (GO:0033209) | 0.01 | 2.03 | -1.14 | 5.31 | PSMC6;PSMD7;CD40LG;PSMD14;TNFRSF12A;TNFSF4;CD27;TNFRSF25;TNFRSF13C;LTB;PSMA7;LIMS1 | |  | | |
| B cell proliferation (GO:0042100) | 0.01 | 2.01 | -1.80 | 8.33 | CD79A;CD40LG;LEF1;BCL2;MS4A1 | |  | | |
| phosphatidic acid metabolic process (GO:0046473) | 0.01 | 1.99 | -1.62 | 7.42 | PLA2G12A;LPCAT4;PLA2G4B;DGKA;LIPH;AGPAT1 | |  | | |
| regulation of calcium-mediated signaling (GO:0050848) | 0.01 | 1.99 | -1.33 | 6.11 | P2RX5;TRAT1;RCAN3;PLEK;CCL3;SLA2 | |  | | |
| negative regulation of sequence-specific DNA binding transcription factor activity (GO:0043433) | 0.01 | 1.98 | -1.48 | 6.77 | TRIM40;TNFAIP3;ARRB2;KLF4;SMAD7;TNFSF4;ID2;ZC3H12A;ID1;BHLHE40;FLNA;NLRP3;SIK1 | |  | | |
| regulation of cytoskeleton organization (GO:0051493) | 0.01 | 1.98 | -1.38 | 6.31 | CCDC88A;CLIC4;HIP1R;ARHGAP18;STMN3;NEXN;PDGFA;RHOU;RHOBTB1;PTK2 | |  | | |
| neutrophil activation involved in immune response (GO:0002283) | 0.01 | 1.98 | -1.05 | 4.79 | ASAH1;PSMD14;GSTP1;HBB;PTPRJ;SLC2A3;IQGAP1;GPR84;IQGAP2;RAP1B;NCSTN;NRAS;PGRMC1;PSMD7;PNP;FTH1;STOM;SNAP29;CTSA;DSP;PLAUR;ANO6;PPBP;PRDX6;RAP2B;RAB31;RAB37;DNASE1;DOCK2;YPEL5;VCL;HSPA1B;HSPA1A | |  | | |
| positive regulation of protein kinase activity (GO:0045860) | 0.01 | 1.97 | -1.15 | 5.24 | EGR1;CDKN1A;MMD;ITGB3;NAB2;IQGAP1;ADRB2;CLU;PTK2;VEGFA;TCL1A;CCL5;CALM3;CCR7;SNCA | |  | | |
| negative regulation of lymphocyte activation (GO:0051250) | 0.01 | 1.97 | -2.08 | 9.44 | TNFAIP3;PTPN22;SLA2;LAX1 | |  | | |
| regulation of platelet activation (GO:0010543) | 0.01 | 1.97 | -1.84 | 8.35 | SELP;THBD;PLEK;PDGFA | |  | | |
| cytoplasmic translation (GO:0002181) | 0.01 | 1.97 | -1.50 | 6.79 | RPL30;RPL32;RPL27A;RPL36A;RPS20;RPS21;RPL7 | |  | | |
| negative regulation of homotypic cell-cell adhesion (GO:0034111) | 0.01 | 1.95 | -2.73 | 12.30 | ZNF703;CD9;ALOX12 | |  | | |
| negative regulation of inclusion body assembly (GO:0090084) | 0.01 | 1.95 | -2.54 | 11.44 | DNAJB6;HSPA1B;HSPA1A | |  | | |
| negative regulation of transcription from RNA polymerase II promoter in response to stress (GO:0097201) | 0.01 | 1.95 | -2.52 | 11.36 | JUN;NCK1;HSPA1A | |  | | |
| T cell chemotaxis (GO:0010818) | 0.01 | 1.95 | -2.52 | 11.34 | GPR183;CCL3;CXCL16 | |  | | |
| negative regulation of cell activation (GO:0050866) | 0.01 | 1.95 | -2.29 | 10.33 | THBD;PDGFA;LDLR | |  | | |
| positive regulation of T cell receptor signaling pathway (GO:0050862) | 0.01 | 1.95 | -2.26 | 10.18 | TESPA1;TRAT1;CD226 | |  | | |
| 3'-UTR-mediated mRNA destabilization (GO:0061158) | 0.01 | 1.95 | -2.25 | 10.11 | ZC3H12D;ZC3H12A;ZFP36L1 | |  | | |
| DNA replication-dependent nucleosome assembly (GO:0006335) | 0.01 | 1.95 | -1.91 | 8.61 | HIST1H4A;HIST1H3A;ASF1A | |  | | |
| DNA replication-dependent nucleosome organization (GO:0034723) | 0.01 | 1.95 | -1.47 | 6.64 | HIST1H4A;HIST1H3A;ASF1A | |  | | |
| regulation of blood coagulation (GO:0030193) | 0.01 | 1.95 | -2.03 | 9.10 | THBD;F2R;GP1BA;TFPI;THBS1 | |  | | |
| regulation of cell migration involved in sprouting angiogenesis (GO:0090049) | 0.01 | 1.95 | -1.77 | 7.95 | TBXA2R;PTGS2;KLF4;THBS1;VEGFA | |  | | |
| positive regulation of reactive oxygen species biosynthetic process (GO:1903428) | 0.01 | 1.95 | -1.71 | 7.67 | HBB;PTGS2;KLF4;CLU;KLF2 | |  | | |
| regulation of cell adhesion mediated by integrin (GO:0033628) | 0.01 | 1.95 | -1.66 | 7.46 | DPP4;PDE3B;ADAM9;PTK2;FERMT3 | |  | | |
| negative regulation of stress-activated MAPK cascade (GO:0032873) | 0.01 | 1.95 | -1.62 | 7.27 | PER1;DUSP10;MYC;GSTP1;PTPN22 | |  | | |
| regulation of actin filament polymerization (GO:0030833) | 0.01 | 1.94 | -1.23 | 5.50 | VIL1;MTPN;CTTN;HIP1R;ARHGAP18;RHOH;CCR7;NCK1 | |  | | |
| regulation of epithelial to mesenchymal transition (GO:0010717) | 0.01 | 1.94 | -1.16 | 5.20 | DAB2;TGFB1I1;LEF1;ZNF703;SNAI1;CTNNB1;AXIN2;SMAD7 | |  | | |
| negative regulation of cysteine-type endopeptidase activity involved in apoptotic process (GO:0043154) | 0.01 | 1.94 | -1.08 | 4.84 | CDKN2D;DNAJB6;LEF1;PLAUR;KLF4;THBS1;SNCA;VEGFA | |  | | |
| neutrophil mediated immunity (GO:0002446) | 0.01 | 1.93 | -1.54 | 6.85 | ASAH1;PSMD14;GSTP1;HBB;PTPRJ;SLC2A3;IQGAP1;GPR84;IQGAP2;CXCL5;RAP1B;NCSTN;NRAS;PGRMC1;PSMD7;PNP;FTH1;STOM;SNAP29;CTSA;DSP;PLAUR;ANO6;PPBP;PRDX6;RAP2B;RAB31;RAB37;DOCK2;YPEL5;VCL;HSPA1B;HSPA1A | |  | | |
| nuclear-transcribed mRNA catabolic process (GO:0000956) | 0.01 | 1.93 | -1.06 | 4.72 | RPL30;RPL32;RPL36A;RPL7;ZFP36L1;RPS25;RPS27;RPS15A;RPL27A;ZC3H12A;RPS20;RPS21;RPS13;DCP2;EIF4G1 | |  | | |
| cellular response to oxygen-containing compound (GO:1901701) | 0.01 | 1.93 | -0.98 | 4.37 | P2RY12;EGR1;JUN;GSTP1;TPM1;TNFAIP3;NR1D1;AHR;HSP90B1;ZFP36L1;RAP1B;NR4A3;SESN3;KAT5;TNFSF4;ZC3H12A;NRIP1;ZNF703;PDE4B;ADAM9;NLRP3 | |  | | |
| positive regulation of programmed cell death (GO:0043068) | 0.01 | 1.93 | -1.07 | 4.76 | BARD1;ITGB1;KLF11;JUN;TNFAIP8;ARHGEF12;TNFRSF12A;HIP1R;ARHGEF18;ANO6;GNA13;GZMK;NCSTN;OBSCN;PNMA1;BCL6;CTNNB1;PMAIP1;ITGA6;SNCA | |  | | |
| response to tumor necrosis factor (GO:0034612) | 0.01 | 1.92 | -1.16 | 5.12 | TCL1A;DHX9;CCL20;CCL5;ZC3H12A;CCL3;ADAM9;GATA3;CXCL16;ZFP36L1 | |  | | |
| phosphatidylinositol metabolic process (GO:0046488) | 0.01 | 1.92 | -1.25 | 5.52 | RUFY1;INPP4B;ARF1;PLA2G12A;PITPNM2;PLEK;TTC7B;IP6K1;MTMR6;PI4K2A;PIK3C2B | |  | | |
| regulation of endocytosis (GO:0030100) | 0.01 | 1.91 | -1.32 | 5.78 | RUFY1;DAB2;RAB20;MCTP1;HIP1R;STON2;LDLR;SNCA | |  | | |
| response to organic cyclic compound (GO:0014070) | 0.01 | 1.91 | -1.21 | 5.33 | CDKN2D;CHRNA2;CCL5;BCL2;CCL3;CTNNB1;PMAIP1;AQP3 | |  | | |
| regulation of endothelial cell migration (GO:0010594) | 0.01 | 1.91 | -1.17 | 5.14 | SPARC;ANGPT1;ITGB3;ZC3H12A;GATA3;THBS1;PTK2;VEGFA | |  | | |
| inactivation of MAPK activity (GO:0000188) | 0.01 | 1.89 | -2.36 | 10.30 | DUSP2;DUSP10;LAX1;DUSP16 | |  | | |
| cell projection assembly (GO:0030031) | 0.01 | 1.89 | -2.12 | 9.25 | PDGFA;RHOH;RHOU;PARVB | |  | | |
| negative regulation of macromolecule biosynthetic process (GO:0010558) | 0.01 | 1.89 | -1.92 | 8.39 | CIITA;GSTP1;NR1D1;PF4 | |  | | |
| regulation of regulatory T cell differentiation (GO:0045589) | 0.01 | 1.89 | -1.82 | 7.96 | DUSP10;BCL6;TNFSF4;IL2RA | |  | | |
| regulation of B cell proliferation (GO:0030888) | 0.01 | 1.89 | -1.66 | 7.24 | TFRC;GPR183;BCL2;CD38;AHR;IKZF3 | |  | | |
| negative regulation of protein metabolic process (GO:0051248) | 0.01 | 1.89 | -1.63 | 7.11 | TRIM40;EIF2AK1;ITGB3;FLNA;LDLR;CLU | |  | | |
| positive regulation of interferon-gamma production (GO:0032729) | 0.01 | 1.89 | -1.54 | 6.70 | CD2;TNFSF4;PDE4B;SLAMF6;PTPN22;RASGRP1 | |  | | |
| response to unfolded protein (GO:0006986) | 0.01 | 1.89 | -1.40 | 6.12 | HSPA9;HSPH1;HSPA5;HYOU1;HSPA1B;HSPA1A | |  | | |
| positive regulation of supramolecular fiber organization (GO:1902905) | 0.01 | 1.88 | -1.09 | 4.71 | VIL1;APP;CTTN;PLEK;RHOH;SLAIN2;CCR7;CLU;NCK1 | |  | | |
| calcium-mediated signaling (GO:0019722) | 0.01 | 1.87 | -1.81 | 7.79 | SELP;MCTP1;CCL20;PLA2G4B;CCL3;CXCR4;PLCG1;CCR6 | |  | | |
| cellular macromolecule biosynthetic process (GO:0034645) | 0.01 | 1.85 | -1.38 | 5.88 | CREBZF;ALAS2;RPL30;RPL32;RPL36A;RPL7;HELB;RPS15A;KAT5;NT5M;RPS13;BARD1;NCOA1;EGF;THAP1;KIN;RPS25;RPS27;POLG2;RPL27A;BCL3;RPS20;RPS21;EIF4G2;NFKBIB;EIF4G1 | |  | | |
| regulation of T cell activation (GO:0050863) | 0.01 | 1.85 | -1.37 | 5.82 | CD2;SIT1;CCL5;SIRPG;PTPN22;LAX1 | |  | | |
| positive regulation of fatty acid oxidation (GO:0046321) | 0.01 | 1.84 | -2.92 | 12.38 | ABCD2;NR4A3;MLYCD | |  | | |
| positive regulation of mesenchymal cell proliferation (GO:0002053) | 0.01 | 1.84 | -2.39 | 10.14 | MYC;PDGFA;VEGFA | |  | | |
| regulation of inclusion body assembly (GO:0090083) | 0.01 | 1.84 | -2.25 | 9.55 | DNAJB6;HSPA1B;HSPA1A | |  | | |
| isotype switching (GO:0045190) | 0.01 | 1.84 | -2.22 | 9.42 | CD40LG;CCR6;EXOSC3 | |  | | |
| regulation of actin filament depolymerization (GO:0030834) | 0.01 | 1.84 | -2.17 | 9.22 | VIL1;MTPN;PLEK | |  | | |
| positive regulation of endothelial cell apoptotic process (GO:2000353) | 0.01 | 1.84 | -2.00 | 8.47 | CD40LG;ANO6;THBS1 | |  | | |
| negative regulation of JUN kinase activity (GO:0043508) | 0.01 | 1.84 | -1.68 | 7.13 | DUSP10;GSTP1;PTPN22 | |  | | |
| epithelial cell-cell adhesion (GO:0090136) | 0.01 | 1.84 | -1.52 | 6.45 | ITGB5;KIFC3;VCL | |  | | |
| positive regulation of tumor necrosis factor superfamily cytokine production (GO:1903557) | 0.01 | 1.84 | -1.55 | 6.54 | CD2;CCL3;RASGRP1;CLU;PF4 | |  | | |
| phagosome maturation (GO:0090382) | 0.01 | 1.84 | -1.36 | 5.74 | RAB32;ATP6V1A;RAB20;RAB31;ATP6V1F | |  | | |
| negative regulation of G-protein coupled receptor protein signaling pathway (GO:0045744) | 0.02 | 1.82 | -1.96 | 8.24 | CCL5;PLEK;LY6G6E;SNCA | |  | | |
| positive regulation of receptor internalization (GO:0002092) | 0.02 | 1.82 | -1.55 | 6.49 | ANGPT1;EGF;ARRB2;VEGFA | |  | | |
| neutrophil degranulation (GO:0043312) | 0.02 | 1.81 | -1.53 | 6.38 | ASAH1;PSMD14;GSTP1;HBB;PTPRJ;SLC2A3;IQGAP1;GPR84;IQGAP2;RAP1B;NCSTN;NRAS;PGRMC1;PSMD7;PNP;FTH1;STOM;SNAP29;CTSA;DSP;PLAUR;ANO6;PPBP;PRDX6;RAP2B;RAB31;RAB37;DOCK2;YPEL5;VCL;HSPA1B;HSPA1A | |  | | |
| regulation of epithelial cell proliferation (GO:0050678) | 0.02 | 1.80 | -1.73 | 7.21 | NR4A1;DUSP10;NR4A3;MYC;NR1D1;BMP6;ZFP36L1;VEGFA | |  | | |
| regulation of neuron death (GO:1901214) | 0.02 | 1.80 | -1.30 | 5.40 | EGR1;EGLN3;CCL5;DDIT4;IKBKG;CLU;SNCA;EIF4G1 | |  | | |
| peptidyl-tyrosine autophosphorylation (GO:0038083) | 0.02 | 1.80 | -1.57 | 6.50 | BLK;ITK;GRAP2;GRAP;SLA2;PTK2 | |  | | |
| positive regulation of blood vessel endothelial cell migration (GO:0043536) | 0.02 | 1.80 | -1.47 | 6.12 | ANGPT1;PLCG1;PTGS2;THBS1;HIF1A;VEGFA | |  | | |
| glycerophospholipid biosynthetic process (GO:0046474) | 0.02 | 1.80 | -1.60 | 6.65 | ARF1;PLA2G12A;PCYT1B;PLA2G4B;DGKA;AGPAT1;MTMR6;PIK3C2B;RUFY1;INPP4B;LPCAT4;LIPH;PITPNM2;PI4K2A | |  | | |
| regulation of MAP kinase activity (GO:0043405) | 0.02 | 1.79 | -1.65 | 6.80 | EGF;SMPD1;GSTP1;STK38;PDGFA;PTPRJ;RASGRP1;TRIB2;VEGFA | |  | | |
| regulation of interleukin-12 production (GO:0032655) | 0.02 | 1.78 | -1.68 | 6.90 | CD40LG;RIPK2;TNFSF4;CCR7;THBS1 | |  | | |
| regulation of T cell differentiation (GO:0045580) | 0.02 | 1.78 | -1.49 | 6.10 | CD2;IL2RA;CAMK4;GATA3;PRDM1 | |  | | |
| positive regulation of sequence-specific DNA binding transcription factor activity (GO:0051091) | 0.02 | 1.77 | -1.05 | 4.30 | MTPN;APP;DHX9;RIPK2;ARID5B;CLU;VEGFA;RPS6KA5;CD40LG;TRAF5;CTNNB1;NLRP3;EP300;IKBKG;HSPA1B;NFKBIB;HSPA1A | |  | | |
| negative regulation of protein modification by small protein conjugation or removal (GO:1903321) | 0.02 | 1.76 | -1.75 | 7.08 | CTNNB1;TNFAIP3;ARRB2;HSPA1B;HSPA1A;SMAD7 | |  | | |
| regulation of cellular component movement (GO:0051270) | 0.02 | 1.76 | -1.26 | 5.11 | EGF;ACTN1;ARHGAP18;CD9;VEGFA;HBEGF | |  | | |
| regulation of calcium ion import (GO:0090279) | 0.02 | 1.75 | -1.97 | 7.94 | EGF;CCL3;CTNNB1;SLC30A1 | |  | | |
| regulation of natural killer cell mediated cytotoxicity (GO:0042269) | 0.02 | 1.75 | -1.92 | 7.74 | SLAMF6;CD226;ARRB2;RASGRP1 | |  | | |
| regulation of vascular endothelial growth factor receptor signaling pathway (GO:0030947) | 0.02 | 1.75 | -1.91 | 7.72 | TMEM204;ITGB3;HIF1A;VEGFA | |  | | |
| positive regulation of erythrocyte differentiation (GO:0045648) | 0.02 | 1.75 | -1.70 | 6.86 | TAL1;HIF1A;HSPA1B;HSPA1A | |  | | |
| negative regulation of sprouting angiogenesis (GO:1903671) | 0.02 | 1.75 | -1.65 | 6.68 | TBXA2R;KLF4;THBS1;KLF2 | |  | | |
| protein localization to cell surface (GO:0034394) | 0.02 | 1.75 | -1.61 | 6.51 | ANGPT1;CTNNB1;FLNA;VCL | |  | | |
| iron ion transport (GO:0006826) | 0.02 | 1.74 | -2.51 | 10.07 | TFRC;SLC11A2;SLC40A1 | |  | | |
| positive regulation of vascular endothelial growth factor receptor signaling pathway (GO:0030949) | 0.02 | 1.74 | -2.39 | 9.59 | ITGB3;HIF1A;VEGFA | |  | | |
| regulation of nitric-oxide synthase biosynthetic process (GO:0051769) | 0.02 | 1.74 | -2.29 | 9.21 | FCER2;GSTP1;NAMPT | |  | | |
| regulation of mesenchymal cell proliferation (GO:0010464) | 0.02 | 1.74 | -2.29 | 9.19 | MYC;PDGFA;VEGFA | |  | | |
| cellular response to fluid shear stress (GO:0071498) | 0.02 | 1.74 | -2.26 | 9.08 | MAPK7;KLF4;KLF2 | |  | | |
| negative regulation of histone modification (GO:0031057) | 0.02 | 1.74 | -2.10 | 8.45 | TAF7;JARID2;SNCA | |  | | |
| negative regulation by host of viral transcription (GO:0043922) | 0.02 | 1.74 | -2.08 | 8.36 | JUN;CCL5;CCL3 | |  | | |
| regulation of catabolic process (GO:0009894) | 0.02 | 1.74 | -2.06 | 8.27 | ITGB1;ODC1;FURIN | |  | | |
| polyamine metabolic process (GO:0006595) | 0.02 | 1.74 | -2.02 | 8.12 | SMOX;ODC1;SAT1 | |  | | |
| cell chemotaxis (GO:0060326) | 0.02 | 1.74 | -1.25 | 5.01 | NR4A1;CCL20;LEF1;ARRB2;CCR6;VEGFA;HBEGF | |  | | |
| positive regulation of leukocyte migration (GO:0002687) | 0.02 | 1.73 | -1.42 | 5.66 | PPBP;CXCL5;PF4V1;PF4;VEGFA | |  | | |
| negative regulation of DNA binding (GO:0043392) | 0.02 | 1.73 | -1.36 | 5.42 | JUN;SUMO3;ID2;ID1;LEF1 | |  | | |
| regulation of interleukin-1 beta secretion (GO:0050706) | 0.02 | 1.73 | -1.21 | 4.81 | RIPK2;ZC3H12A;CCL3;NLRP3;CCR7 | |  | | |
| positive regulation of transcription from RNA polymerase II promoter (GO:0045944) | 0.02 | 1.73 | -1.36 | 5.42 | CSRNP1;APP;CCNK;CIITA;CCNH;AHR;CD3D;IKZF3;IRF2BPL;SPIB;RPS6KA5;KAT5;MYC;ZC3H12A;NAMPT;EP300;IKBKG;NCK1;KDM6B;NCOA1;RIPK2;TAL1;ITGA6;ATF3;DHX9;LEF1;GATA3;HIF1A;MAPK7;NRIP1;NLRP3;EGR1;JUN;BCL11B;PCGF5;OSM;NR1D1;KLF4;PBX1;KLF2;BMP6;FOSL2;VEGFA;SMAD7;NR4A1;PER1;NR4A3;POU2AF1;CTNNB1;TAF7;PF4 | |  | | |
| regulation of intrinsic apoptotic signaling pathway (GO:2001242) | 0.02 | 1.72 | -1.30 | 5.15 | URI1;BCL2;PLAUR;PPIF;PMAIP1;MCL1 | |  | | |
| negative regulation of I-kappaB kinase/NF-kappaB signaling (GO:0043124) | 0.02 | 1.72 | -1.27 | 5.01 | PER1;GSTP1;ZC3H12A;RHOH;TNFAIP3;NLRP3 | |  | | |
| protein stabilization (GO:0050821) | 0.02 | 1.71 | -0.99 | 3.90 | CDKN1A;CCNH;HIP1R;NLK;CLU;HCFC1;SMAD7;TMEM88;FLNA;EP300;PIM2;HSPA1B;HSPA1A | |  | | |
| positive regulation of multicellular organismal process (GO:0051240) | 0.02 | 1.71 | -1.27 | 5.00 | TFRC;TGFB1I1;LEF1;AXIN2;AGPAT1;HIF1A;RAB11A;VIL1;DAB2;TNFSF4;ZNF703;SNAI1;CTNNB1;PLCG1;LDLR;EIF4G1 | |  | | |
| positive regulation of protein oligomerization (GO:0032461) | 0.02 | 1.69 | -1.77 | 6.89 | TCL1A;PMAIP1;CLU;TRABD2A | |  | | |
| regulation of protein oligomerization (GO:0032459) | 0.02 | 1.69 | -1.59 | 6.18 | TCL1A;EP300;PMAIP1;TRABD2A | |  | | |
| endothelial cell migration (GO:0043542) | 0.02 | 1.68 | -1.57 | 6.09 | DPP4;NR4A1;ID1;MYH9;VEGFA | |  | | |
| lipid phosphorylation (GO:0046834) | 0.02 | 1.68 | -1.44 | 5.57 | DGKA;TTC7B;IP6K1;PI4K2A;PIK3C2B | |  | | |
| negative regulation of mitotic cell cycle (GO:0045930) | 0.02 | 1.68 | -1.46 | 5.65 | BTG2;BCL6;PPP1R10;CTNNB1;CDC14A;ZFP36L1 | |  | | |
| positive regulation of chemotaxis (GO:0050921) | 0.02 | 1.68 | -1.22 | 4.72 | PPBP;THBS1;CXCL5;PF4V1;PF4;VEGFA | |  | | |
| regulation of hematopoietic stem cell differentiation (GO:1902036) | 0.02 | 1.68 | -1.12 | 4.34 | HIST1H4A;PSMC6;PSMD7;HIST1H3A;PSMD14;TAL1;GATA3;PSMA7 | |  | | |
| protein transport (GO:0015031) | 0.02 | 1.67 | -1.37 | 5.28 | CTSA;ATP6V1A;UNC119B;RABGAP1L;TFRC;CD3G;RAB27B;CLU;RAB11A;STX11;HSP90B1;RAB32;RUFY1;ARFRP1;RAB20;RAB31;CTTN;RAB37;MYH9;RAB6B;SEC31A;YWHAH;ATP6V1F | |  | | |
| second-messenger-mediated signaling (GO:0019932) | 0.02 | 1.67 | -1.53 | 5.88 | MCTP1;PLA2G4B;CCL3;CXCR4;PLCG1;CCR6;KLRG1 | |  | | |
| response to cytokine (GO:0034097) | 0.02 | 1.67 | -0.99 | 3.79 | PTPN18;JUN;CIITA;LEF1;BCL2;CXCR4;ADAM9;CCR7;TRIM56;MCL1;SNCA;CXCL16 | |  | | |
| B cell activation involved in immune response (GO:0002312) | 0.02 | 1.65 | -2.33 | 8.88 | CD40LG;GPR183;EXOSC3 | |  | | |
| regulation of lymphocyte activation (GO:0051249) | 0.02 | 1.65 | -2.32 | 8.83 | SIT1;CCL5;IKZF3 | |  | | |
| cellular response to low-density lipoprotein particle stimulus (GO:0071404) | 0.02 | 1.65 | -2.21 | 8.42 | ITGB1;CD9;LDLR | |  | | |
| regulation of granulocyte chemotaxis (GO:0071622) | 0.02 | 1.65 | -2.15 | 8.18 | CCL5;THBS1;JAM3 | |  | | |
| glycerolipid catabolic process (GO:0046503) | 0.02 | 1.65 | -2.03 | 7.74 | PLA2G4B;PRDX6;MGLL | |  | | |
| regulation of interleukin-1 beta production (GO:0032651) | 0.02 | 1.65 | -1.95 | 7.44 | GSTP1;F2R;CCR7 | |  | | |
| positive regulation of epithelial cell proliferation (GO:0050679) | 0.02 | 1.65 | -1.10 | 4.20 | NR4A1;NRAS;NR4A3;ITGB3;MYC;ZNF703;NRARP;HIF1A;BMP6;VEGFA | |  | | |
| regulation of response to external stimulus (GO:0032101) | 0.02 | 1.65 | -0.99 | 3.76 | FNTA;BCL6;TNFSF4;F2R;CXCR4;TNFAIP3;NLRP3;GP1BA;PTGS2;MGLL | |  | | |
| regulation of hematopoietic progenitor cell differentiation (GO:1901532) | 0.02 | 1.65 | -1.71 | 6.48 | HIST1H4A;PSMC6;PSMD7;HIST1H3A;PSMD14;TAL1;GATA3;PSMA7 | |  | | |
| lymphocyte proliferation (GO:0046651) | 0.02 | 1.63 | -1.41 | 5.30 | CD79A;CD40LG;LEF1;BCL2;MS4A1 | |  | | |
| negative regulation of blood coagulation (GO:0030195) | 0.02 | 1.63 | -1.28 | 4.82 | THBD;PLAUR;PDGFA;GP1BA;TFPI | |  | | |
| mRNA splice site selection (GO:0006376) | 0.02 | 1.63 | -1.92 | 7.19 | SF3A1;PRPF39;LUC7L3;LUC7L | |  | | |
| negative regulation of endothelial cell apoptotic process (GO:2000352) | 0.02 | 1.63 | -1.64 | 6.16 | MAPK7;ANGPT1;TNFAIP3;GATA3 | |  | | |
| regulation of small GTPase mediated signal transduction (GO:0051056) | 0.02 | 1.62 | -1.03 | 3.84 | GNA13;ARHGAP21;ARHGEF12;OBSCN;ARHGAP18;RHOH;ARHGEF18;RHOU;ARHGAP15;ARHGAP26;ARHGAP6;RHOBTB1 | |  | | |
| MAPK cascade (GO:0000165) | 0.03 | 1.60 | -0.87 | 3.21 | PSMD14;ANGPT1;EGF;RIPK2;PDGFA;RASGRP1;PSMA7;PTK2;ZFP36L1;MAPK13;NRAS;PSMC6;PSMD7;SYNGAP1;IL2RA;CCL5;MYC;CCL3;IKBKG;HBEGF | |  | | |
| positive regulation of mitochondrial outer membrane permeabilization involved in apoptotic signaling pathway (GO:1901030) | 0.03 | 1.59 | -1.76 | 6.45 | PPP3CC;BCL2;HIP1R;PMAIP1;YWHAH | |  | | |
| regulation of endothelial cell proliferation (GO:0001936) | 0.03 | 1.59 | -1.13 | 4.13 | NR4A1;NRAS;SPARC;ITGB3;NRARP;THBS1;HIF1A;VEGFA | |  | | |
| response to calcium ion (GO:0051592) | 0.03 | 1.59 | -1.07 | 3.91 | CLIC4;PPIF;ADAM9;CALM3;IQGAP1;THBS1;AQP3;SEC31A | |  | | |
| negative regulation of wound healing (GO:0061045) | 0.03 | 1.57 | -1.72 | 6.23 | THBD;DUSP10;CASK;TFPI | |  | | |
| heterotypic cell-cell adhesion (GO:0034113) | 0.03 | 1.57 | -1.65 | 5.96 | CD2;ITGB1;DSP;ITGB3 | |  | | |
| cellular response to epidermal growth factor stimulus (GO:0071364) | 0.03 | 1.57 | -1.64 | 5.94 | VIL1;PLCG1;IQGAP1;ZFP36L1 | |  | | |
| positive regulation of peptidyl-threonine phosphorylation (GO:0010800) | 0.03 | 1.57 | -1.57 | 5.68 | APP;EGF;RIPK2;CALM3 | |  | | |
| negative regulation of gene expression, epigenetic (GO:0045814) | 0.03 | 1.57 | -1.54 | 5.57 | CREBZF;HIST1H4A;HIST1H3A;JARID2 | |  | | |
| negative regulation of focal adhesion assembly (GO:0051895) | 0.03 | 1.57 | -2.36 | 8.54 | THBS1;ARHGAP6;CORO1C | |  | | |
| regulation of protein modification by small protein conjugation or removal (GO:1903320) | 0.03 | 1.57 | -2.29 | 8.27 | EGR1;HSPA1B;HSPA1A | |  | | |
| negative regulation of interleukin-1 beta production (GO:0032691) | 0.03 | 1.57 | -2.05 | 7.40 | GSTP1;ZC3H12A;NLRP3 | |  | | |
| negative regulation of cell migration involved in sprouting angiogenesis (GO:0090051) | 0.03 | 1.57 | -1.94 | 7.01 | TBXA2R;KLF4;THBS1 | |  | | |
| regulation of lipase activity (GO:0060191) | 0.03 | 1.57 | -1.73 | 6.28 | FURIN;PCSK6;SNCA | |  | | |
| negative regulation of T cell receptor signaling pathway (GO:0050860) | 0.03 | 1.57 | -1.66 | 5.99 | PTPRJ;PTPN22;UBASH3A | |  | | |
| regulation of JAK-STAT cascade (GO:0046425) | 0.03 | 1.57 | -1.34 | 4.85 | SOCS3;LEPROT;CISH;CCL5;BCL3;GP1BA;NLK | |  | | |
| cellular response to reactive oxygen species (GO:0034614) | 0.03 | 1.57 | -1.15 | 4.14 | JUN;MAPK7;TPM1;PPIF;TNFAIP3;ANKZF1;MAPK13 | |  | | |
| arachidonic acid metabolic process (GO:0019369) | 0.03 | 1.56 | -1.68 | 6.06 | PLA2G4B;EPHX2;ALOX12;PTGS2;MGLL;PTGS1 | |  | | |
| regulation of protein serine/threonine kinase activity (GO:0071900) | 0.03 | 1.56 | -1.01 | 3.62 | CDKN2D;CDKN1A;CCNK;TCL1A;CALM3;ADRB2;TRIB2;SNCA | |  | | |
| cellular response to hormone stimulus (GO:0032870) | 0.03 | 1.56 | -1.01 | 3.61 | NCOA1;JUN;URI1;NR4A3;SIK1;TFPI;GAS2L1;ZFP36L1 | |  | | |
| positive regulation of protein metabolic process (GO:0051247) | 0.03 | 1.54 | -1.11 | 3.95 | BARD1;MTPN;APP;MAPK7;TNFAIP3;KLF4;CLU;KLF2;EIF4G1 | |  | | |
| regulation of mRNA stability (GO:0043488) | 0.03 | 1.53 | -1.28 | 4.53 | PSMC6;PSMD7;PSMD14;FASTKD1;AXIN2;EXOSC3;PSMA7;DCP2;ZFP36L1;EIF4G1;HSPA1A | |  | | |
| negative regulation of cell death (GO:0060548) | 0.03 | 1.53 | -1.17 | 4.13 | CTNNB1;IKBKG;CLU;HSPA1B;SNCA;NCK1;EIF4G1;HSPA1A | |  | | |
| cellular response to lipid (GO:0071396) | 0.03 | 1.53 | -0.90 | 3.15 | URI1;GSTP1;TNFAIP3;NR1D1;AHR;TFPI;KAT5;TNFSF4;ZC3H12A;NRIP1;ZNF703;PDE4B;ADAM9;NLRP3 | |  | | |
| regulation of calcium ion transport (GO:0051924) | 0.03 | 1.53 | -1.35 | 4.76 | EGF;CCL5;F2R;BCL2;CCL3;CTNNB1 | |  | | |
| positive regulation of mitotic cell cycle (GO:0045931) | 0.03 | 1.53 | -1.27 | 4.46 | APP;EGF;TAL1;PPP1R10;CD28;CDC25B | |  | | |
| regulation of NF-kappaB import into nucleus (GO:0042345) | 0.03 | 1.52 | -1.69 | 5.91 | APP;BCL3;ZC3H12A;NLRP3 | |  | | |
| regulation of histone acetylation (GO:0035065) | 0.03 | 1.52 | -1.58 | 5.53 | RPS6KA5;TAF7;IWS1;SNCA | |  | | |
| mRNA metabolic process (GO:0016071) | 0.03 | 1.51 | -1.28 | 4.45 | KIN;SF3A1;PCF11;PABPC3;PRPF8;SRSF7;HSPA1B;DCP2;HSPA1A | |  | | |
| cellular response to organonitrogen compound (GO:0071417) | 0.03 | 1.51 | -1.10 | 3.83 | P2RY12;RAP1B;EGR1;SESN3;CTNNB1;AHR;HSP90B1;ZFP36L1;MAPK13 | |  | | |
| negative regulation of protein catabolic process (GO:0042177) | 0.03 | 1.50 | -1.23 | 4.26 | CTSA;RGP1;TRIM40;FLNA;SNCA | |  | | |
| negative regulation of adherens junction organization (GO:1903392) | 0.03 | 1.50 | -2.15 | 7.41 | THBS1;ARHGAP6;CORO1C | |  | | |
| regulation of mRNA metabolic process (GO:1903311) | 0.03 | 1.50 | -2.01 | 6.91 | PTCD2;DHX9;IWS1 | |  | | |
| positive regulation of alpha-beta T cell activation (GO:0046635) | 0.03 | 1.50 | -1.94 | 6.68 | HSPH1;PNP;TNFSF4 | |  | | |
| negative regulation of cellular component movement (GO:0051271) | 0.03 | 1.50 | -1.81 | 6.22 | ACTN1;CD9;GATA3 | |  | | |
| NAD biosynthesis via nicotinamide riboside salvage pathway (GO:0034356) | 0.03 | 1.50 | -1.76 | 6.06 | PNP;NAMPT;PTGS2 | |  | | |
| negative regulation of platelet-derived growth factor receptor signaling pathway (GO:0010642) | 0.03 | 1.50 | -1.60 | 5.51 | PDGFA;PTPRJ;SNCA | |  | | |
| regulation of lymphocyte proliferation (GO:0050670) | 0.03 | 1.50 | -1.59 | 5.48 | PTPN22;AHR;IKZF3 | |  | | |
| positive regulation of protein modification process (GO:0031401) | 0.03 | 1.50 | -0.81 | 2.80 | APP;ITGB3;PLAUR;AXIN2;RASGRP1;PTK2;VEGFA;DAB2;KAT5;SMPD1;CDK2AP1;CALM3;TRABD2A | |  | | |
| positive regulation of phosphatidylinositol 3-kinase signaling (GO:0014068) | 0.03 | 1.49 | -1.24 | 4.27 | SELP;CCL5;F2R;PDGFA;CBL;PTK2 | |  | | |
| positive regulation of protein import into nucleus (GO:0042307) | 0.03 | 1.49 | -1.21 | 4.15 | DAB2;EGF;ZC3H12A;F2R;FLNA;BMP6 | |  | | |
| protein localization to plasma membrane (GO:0072659) | 0.03 | 1.49 | -0.99 | 3.38 | ARFRP1;EHD3;RAB31;TMEM88;RAB37;TTC7B;FLNA;F11R;TSPAN33;ANK1;RAB11A | |  | | |
| phosphatidylcholine acyl-chain remodeling (GO:0036151) | 0.03 | 1.47 | -1.95 | 6.57 | PLA2G12A;LPCAT4;PLA2G4B;LPCAT3 | |  | | |
| muscle fiber development (GO:0048747) | 0.03 | 1.47 | -1.58 | 5.32 | LEF1;NEXN;BEND2;VEGFA | |  | | |
| negative regulation of cell-matrix adhesion (GO:0001953) | 0.03 | 1.47 | -1.53 | 5.16 | CASK;THBS1;ARHGAP6;CORO1C | |  | | |
| phosphatidylinositol phosphorylation (GO:0046854) | 0.03 | 1.47 | -1.29 | 4.37 | TTC7B;IP6K1;PI4K2A;PIK3C2B | |  | | |
| cellular response to oxidative stress (GO:0034599) | 0.03 | 1.46 | -1.48 | 5.00 | JUN;GSTP1;ZC3H12A;TPM1;SOD2;PRDX6;HIF1A;HSPA1B;SNCA;HSPA1A | |  | | |
| positive regulation of Ras protein signal transduction (GO:0046579) | 0.03 | 1.46 | -1.38 | 4.64 | NOTCH2;P2RY10;LPAR6;F2R;RASGRP1 | |  | | |
| androgen receptor signaling pathway (GO:0030521) | 0.03 | 1.46 | -1.33 | 4.47 | NCOA1;KAT5;TGFB1I1;NRIP1;CTNNB1 | |  | | |
| positive regulation of immune response (GO:0050778) | 0.03 | 1.46 | -1.23 | 4.14 | DHX9;TNFSF4;CCL5;NLRP3;CCR7;CD226 | |  | | |
| cellular response to molecule of bacterial origin (GO:0071219) | 0.04 | 1.45 | -1.01 | 3.38 | TNFSF4;GSTP1;ZC3H12A;PDE4B;ADAM9;TNFAIP3;NLRP3;NR1D1 | |  | | |
| negative regulation of systemic arterial blood pressure (GO:0003085) | 0.04 | 1.45 | -3.23 | 10.74 | ADRB2;SOD2 | |  | | |
| regulation of MHC class I biosynthetic process (GO:0045343) | 0.04 | 1.45 | -3.00 | 9.99 | CIITA;HSPH1 | |  | | |
| regulation of response to wounding (GO:1903034) | 0.04 | 1.45 | -2.86 | 9.53 | VIL1;TNFRSF12A | |  | | |
| iron ion import (GO:0097286) | 0.04 | 1.45 | -2.76 | 9.17 | TFRC;SLC11A2 | |  | | |
| positive regulation of deacetylase activity (GO:0090045) | 0.04 | 1.45 | -2.72 | 9.05 | FNTA;VEGFA | |  | | |
| negative regulation of muscle cell apoptotic process (GO:0010656) | 0.04 | 1.45 | -2.70 | 8.98 | ZC3H12A;ALOX12 | |  | | |
| positive regulation of T-helper 2 cell differentiation (GO:0045630) | 0.04 | 1.45 | -2.67 | 8.89 | TNFSF4;NLRP3 | |  | | |
| negative regulation of acute inflammatory response (GO:0002674) | 0.04 | 1.45 | -2.64 | 8.80 | GSTP1;NLRP3 | |  | | |
| positive regulation of blood pressure (GO:0045777) | 0.04 | 1.45 | -2.64 | 8.78 | TBXA2R;ID2 | |  | | |
| acetyl-CoA biosynthetic process (GO:0006085) | 0.04 | 1.45 | -2.61 | 8.70 | MLYCD;ACSS1 | |  | | |
| negative regulation of receptor biosynthetic process (GO:0010871) | 0.04 | 1.45 | -2.56 | 8.53 | ITGB3;NR1D1 | |  | | |
| T-helper 17 cell lineage commitment (GO:0072540) | 0.04 | 1.45 | -2.52 | 8.39 | SLAMF6;LY9 | |  | | |
| immunological synapse formation (GO:0001771) | 0.04 | 1.45 | -2.50 | 8.33 | CD6;CCR7 | |  | | |
| signal complex assembly (GO:0007172) | 0.04 | 1.45 | -2.45 | 8.16 | CD3E;NCK1 | |  | | |
| polyamine biosynthetic process (GO:0006596) | 0.04 | 1.45 | -2.44 | 8.13 | SMOX;SAT1 | |  | | |
| negative regulation of stress-activated protein kinase signaling cascade (GO:0070303) | 0.04 | 1.45 | -2.29 | 7.64 | MYC;GSTP1 | |  | | |
| negative regulation of interleukin-6 secretion (GO:1900165) | 0.04 | 1.45 | -2.29 | 7.63 | ZC3H12A;PTPN22 | |  | | |
| negative regulation of transforming growth factor beta production (GO:0071635) | 0.04 | 1.45 | -2.24 | 7.47 | LAPTM4B;FURIN | |  | | |
| regulation of microtubule nucleation (GO:0010968) | 0.04 | 1.45 | -2.21 | 7.35 | HSPA1B;HSPA1A | |  | | |
| regulation of lysosomal protein catabolic process (GO:1905165) | 0.04 | 1.45 | -2.19 | 7.29 | LAPTM4B;LDLR | |  | | |
| regulation of oxidative stress-induced neuron intrinsic apoptotic signaling pathway (GO:1903376) | 0.04 | 1.45 | -2.17 | 7.22 | HIF1A;MCL1 | |  | | |
| regulation of immunoglobulin mediated immune response (GO:0002889) | 0.04 | 1.45 | -2.17 | 7.22 | TNFSF4;CD226 | |  | | |
| glutamate catabolic process (GO:0006538) | 0.04 | 1.45 | -1.98 | 6.58 | GLUD1;GLUL | |  | | |
| regulation of inositol phosphate biosynthetic process (GO:0010919) | 0.04 | 1.45 | -1.65 | 5.50 | PLEK;SNCA | |  | | |
| regulation of biosynthetic process (GO:0009889) | 0.04 | 1.45 | -1.47 | 4.88 | GSTP1;SNCA | |  | | |
| negative regulation of angiogenesis (GO:0016525) | 0.04 | 1.44 | -1.12 | 3.73 | SPARC;PDE3B;CTNNB1;KLF4;THBS1;KLF2;PF4 | |  | | |
| positive regulation of antigen receptor-mediated signaling pathway (GO:0050857) | 0.04 | 1.43 | -1.96 | 6.43 | TESPA1;TRAT1;CD226 | |  | | |
| positive regulation of leukocyte activation (GO:0002696) | 0.04 | 1.43 | -1.88 | 6.17 | CD2;CD226;THBS1 | |  | | |
| regulation of intrinsic apoptotic signaling pathway in response to DNA damage (GO:1902229) | 0.04 | 1.43 | -1.86 | 6.11 | CDKN2D;SNAI1;CLU | |  | | |
| positive regulation of lymphocyte differentiation (GO:0045621) | 0.04 | 1.43 | -1.86 | 6.11 | MMP14;CD27;GATA3 | |  | | |
| positive regulation of epithelial cell apoptotic process (GO:1904037) | 0.04 | 1.43 | -1.86 | 6.09 | CD40LG;ANO6;THBS1 | |  | | |
| positive regulation of cyclic nucleotide metabolic process (GO:0030801) | 0.04 | 1.43 | -1.81 | 5.95 | CALM3;ADM;PF4 | |  | | |
| neurotransmitter transport (GO:0006836) | 0.04 | 1.43 | -1.79 | 5.89 | SLC38A1;GLUL;SLC18A2 | |  | | |
| ruffle organization (GO:0031529) | 0.04 | 1.43 | -1.77 | 5.80 | TPM1;PLEK;CCR7 | |  | | |
| positive regulation of DNA-templated transcription, initiation (GO:2000144) | 0.04 | 1.43 | -1.64 | 5.37 | FOSL1;JUN;CTNNB1 | |  | | |
| regulation of toll-like receptor 4 signaling pathway (GO:0034143) | 0.04 | 1.43 | -1.58 | 5.17 | TNFAIP3;PTPN22;NR1D1 | |  | | |
| regulation of peptidyl-tyrosine phosphorylation (GO:0050730) | 0.04 | 1.42 | -1.42 | 4.66 | APP;RAP2B;ANGPT1;RIPK2;ITGB3;OSM;PDGFA;VEGFA | |  | | |
| negative regulation of STAT cascade (GO:1904893) | 0.04 | 1.42 | -1.37 | 4.48 | SOCS3;LEPROT;CISH;BCL3;GP1BA | |  | | |
| cellular response to peptide (GO:1901653) | 0.04 | 1.42 | -1.32 | 4.31 | APP;RIPK2;PTPN22;ADRB2;ZFP36L1 | |  | | |
| regulation of NIK/NF-kappaB signaling (GO:1901222) | 0.04 | 1.42 | -1.57 | 5.11 | APP;TRIM40;PTPN22;LIMS1 | |  | | |
| positive regulation of fibroblast proliferation (GO:0048146) | 0.04 | 1.42 | -1.52 | 4.96 | CDKN1A;DHX9;MYC;PDGFA | |  | | |
| positive regulation of calcium-mediated signaling (GO:0050850) | 0.04 | 1.42 | -1.43 | 4.68 | P2RX5;TRAT1;CCL3;CALM3 | |  | | |
| negative regulation of JNK cascade (GO:0046329) | 0.04 | 1.42 | -1.37 | 4.46 | PER1;DUSP10;GSTP1;PTPN22 | |  | | |
| positive regulation of extrinsic apoptotic signaling pathway (GO:2001238) | 0.04 | 1.42 | -1.32 | 4.30 | TNFRSF12A;G0S2;PMAIP1;THBS1 | |  | | |
| regulation of defense response to virus by virus (GO:0050690) | 0.04 | 1.42 | -1.17 | 3.80 | ARF1;CD8B;CD28;DOCK2 | |  | | |
| positive regulation of proteasomal ubiquitin-dependent protein catabolic process (GO:0032436) | 0.04 | 1.42 | -1.14 | 3.73 | PLK3;DAB2;TRIB2;CLU;HSPA1B;HSPA1A;SMAD7 | |  | | |
| Rab protein signal transduction (GO:0032482) | 0.04 | 1.42 | -1.00 | 3.25 | RAB32;RAB20;RAB31;RAB37;RAB27B;RAB6B;RAB11A | |  | | |
| rRNA processing (GO:0006364) | 0.04 | 1.42 | -1.10 | 3.60 | UTP6;RPL30;RPL32;NOP2;RPL36A;RPL7;RPS25;EMG1;RPS27;RPS15A;RPL27A;RPS20;EXOSC3;RPS21;RPS13 | |  | | |
| positive regulation of protein serine/threonine kinase activity (GO:0071902) | 0.04 | 1.40 | -1.26 | 4.05 | CCNK;TCL1A;EGF;CCNH;PDGFA;CALM3;ADRB2;RASGRP1;SNCA;VEGFA | |  | | |
| plasma membrane bounded cell projection organization (GO:0120036) | 0.04 | 1.40 | -0.86 | 2.78 | DBNDD1;APP;BLOC1S5;BTG2;CTTN;TPM1;PLEK;STMN3;CCR7;RAB11A | |  | | |
| cellular defense response (GO:0006968) | 0.04 | 1.39 | -1.37 | 4.39 | ITGB1;FOSL1;ITK;TRAT1;CCR6;KLRG1 | |  | | |
| negative regulation of cellular catabolic process (GO:0031330) | 0.04 | 1.39 | -0.93 | 2.99 | RGP1;BCL2;PTPN22;EIF4G2;MCL1;EIF4G1 | |  | | |
| positive regulation of growth (GO:0045927) | 0.04 | 1.39 | -1.09 | 3.48 | MTPN;MMP14;LEF1;BCL2;EIF4G2;CXCL16;EIF4G1 | |  | | |
| regulation of I-kappaB kinase/NF-kappaB signaling (GO:0043122) | 0.04 | 1.38 | -0.92 | 2.93 | RIPK2;GSTP1;F2R;RHOH;TNFAIP3;ATP2C1;PER1;BCL3;ZC3H12A;TRAF5;FLNA;NLRP3;CCR7;IKBKG;PIM2 | |  | | |
| positive regulation of epidermal growth factor receptor signaling pathway (GO:0045742) | 0.04 | 1.37 | -1.66 | 5.23 | EGF;HIP1R;PLAUR;CBL | |  | | |
| negative regulation of epithelial cell apoptotic process (GO:1904036) | 0.04 | 1.37 | -1.55 | 4.88 | MAPK7;ANGPT1;TNFAIP3;GATA3 | |  | | |
| positive regulation of myeloid cell differentiation (GO:0045639) | 0.04 | 1.37 | -1.44 | 4.53 | TAL1;HIF1A;HSPA1B;HSPA1A | |  | | |
| negative regulation of cysteine-type endopeptidase activity (GO:2000117) | 0.04 | 1.36 | -0.88 | 2.76 | CDKN2D;DNAJB6;LEF1;KLF4;THBS1;SNCA;VEGFA | |  | | |
| pigment granule transport (GO:0051904) | 0.04 | 1.36 | -2.29 | 7.19 | BLOC1S5;RAB27B;RAB11A | |  | | |
| melanosome transport (GO:0032402) | 0.04 | 1.36 | -2.24 | 7.01 | BLOC1S5;RAB27B;RAB11A | |  | | |
| positive regulation of natural killer cell mediated cytotoxicity (GO:0045954) | 0.04 | 1.36 | -2.04 | 6.40 | SLAMF6;CD226;RASGRP1 | |  | | |
| negative regulation of interleukin-8 production (GO:0032717) | 0.04 | 1.36 | -1.83 | 5.74 | BCL3;PTPN22;KLF4 | |  | | |
| positive regulation of focal adhesion assembly (GO:0051894) | 0.04 | 1.36 | -1.75 | 5.49 | PTPRJ;LIMS1;VEGFA | |  | | |
| DNA catabolic process (GO:0006308) | 0.04 | 1.36 | -1.75 | 5.48 | ISG20;MUS81;DNASE1 | |  | | |
| positive regulation of natural killer cell mediated immunity (GO:0002717) | 0.04 | 1.36 | -1.67 | 5.23 | SLAMF6;CD226;RASGRP1 | |  | | |
| positive chemotaxis (GO:0050918) | 0.04 | 1.36 | -1.66 | 5.20 | ANGPT1;PTPRJ;VEGFA | |  | | |
| phospholipid catabolic process (GO:0009395) | 0.04 | 1.36 | -1.63 | 5.10 | PLA2G4B;SMPD1;PRDX6 | |  | | |
| mRNA cis splicing, via spliceosome (GO:0045292) | 0.04 | 1.36 | -1.56 | 4.88 | PRPF39;SNRNP200;WBP11 | |  | | |
| negative regulation of cellular carbohydrate metabolic process (GO:0010677) | 0.04 | 1.36 | -1.47 | 4.59 | PLEK;SIK1;CLK2 | |  | | |
| negative regulation of antigen receptor-mediated signaling pathway (GO:0050858) | 0.04 | 1.36 | -1.35 | 4.23 | PTPRJ;PTPN22;UBASH3A | |  | | |
| positive regulation by host of viral transcription (GO:0043923) | 0.04 | 1.36 | -1.15 | 3.61 | JUN;LEF1;EP300 | |  | | |
| actin filament organization (GO:0007015) | 0.04 | 1.36 | -0.90 | 2.81 | TPM4;ACTN1;ABI1;TPM1;HIP1R;RHOH;FLNA;NEDD9;RHOU;RHOBTB1 | |  | | |
| regulation of stem cell differentiation (GO:2000736) | 0.04 | 1.35 | -1.32 | 4.11 | HIST1H4A;PSMC6;PSMD7;HIST1H3A;PSMD14;TAL1;GATA3;PSMA7 | |  | | |
| negative regulation of ERBB signaling pathway (GO:1901185) | 0.04 | 1.35 | -1.39 | 4.31 | PTPN18;EGF;PTPRJ;CBL;HBEGF | |  | | |
| branching morphogenesis of an epithelial tube (GO:0048754) | 0.04 | 1.35 | -1.32 | 4.08 | MYC;LEF1;CTNNB1;NRARP;VEGFA | |  | | |
| intrinsic apoptotic signaling pathway by p53 class mediator (GO:0072332) | 0.04 | 1.35 | -1.20 | 3.74 | BCL3;DDIT4;EP300;PMAIP1;ZNF385D | |  | | |
| substantia nigra development (GO:0021762) | 0.04 | 1.35 | -1.09 | 3.38 | GLUD1;HSPA5;SEC16A;CALM3;YWHAH | |  | | |
| regulation of cell differentiation (GO:0045595) | 0.05 | 1.34 | -1.25 | 3.84 | HEMGN;JUN;BCL6;PLEKHB1;SIK1;NR1D1;KLF4;SKIL;PTK2;SMAD7 | |  | | |
| positive regulation of inflammatory response (GO:0050729) | 0.05 | 1.33 | -1.26 | 3.87 | CD6;DHX9;TNFSF4;OSM;CCL3;SNCA;MAPK13 | |  | | |
| lymphocyte differentiation (GO:0030098) | 0.05 | 1.33 | -1.09 | 3.36 | BLK;ITGB1;CD79B;CD79A;RHOH;PTPN22;CD3D | |  | | |
| regulation of toll-like receptor 3 signaling pathway (GO:0034139) | 0.05 | 1.33 | -3.44 | 10.56 | TNFAIP3;PTPN22 | |  | | |
| regulation of hormone biosynthetic process (GO:0046885) | 0.05 | 1.33 | -3.28 | 10.06 | EGR1;HIF1A | |  | | |
| negative regulation of macrophage activation (GO:0043031) | 0.05 | 1.33 | -3.13 | 9.62 | ZC3H12A;LDLR | |  | | |
| positive regulation of tumor necrosis factor-mediated signaling pathway (GO:1903265) | 0.05 | 1.33 | -2.99 | 9.18 | HSPA1B;HSPA1A | |  | | |
| regulation of alpha-beta T cell differentiation (GO:0046637) | 0.05 | 1.33 | -2.77 | 8.51 | PNP;PRDM1 | |  | | |
| positive regulation of neuroblast proliferation (GO:0002052) | 0.05 | 1.33 | -2.73 | 8.39 | CTNNB1;VEGFA | |  | | |
| positive regulation of B cell differentiation (GO:0045579) | 0.05 | 1.33 | -2.65 | 8.14 | MMP14;CD27 | |  | | |
| oxygen transport (GO:0015671) | 0.05 | 1.33 | -2.60 | 7.99 | MYC;HBB | |  | | |
| regulation of lymphocyte migration (GO:2000401) | 0.05 | 1.33 | -2.56 | 7.85 | CCR6;GCSAM | |  | | |
| regulation of microglial cell activation (GO:1903978) | 0.05 | 1.33 | -2.51 | 7.70 | APP;LDLR | |  | | |
| positive regulation of macrophage migration (GO:1905523) | 0.05 | 1.33 | -2.49 | 7.66 | CCL5;THBS1 | |  | | |
| regulation of chromatin assembly or disassembly (GO:0001672) | 0.05 | 1.33 | -2.48 | 7.63 | TAL1;SPTY2D1 | |  | | |
| negative regulation of transporter activity (GO:0032410) | 0.05 | 1.33 | -2.47 | 7.59 | PPIF;SNCA | |  | | |
| regulation of cyclic-nucleotide phosphodiesterase activity (GO:0051342) | 0.05 | 1.33 | -2.46 | 7.54 | MAPK7;CALM3 | |  | | |
| positive regulation of macrophage chemotaxis (GO:0010759) | 0.05 | 1.33 | -2.33 | 7.17 | CCL5;THBS1 | |  | | |
| regulation of interleukin-13 production (GO:0032656) | 0.05 | 1.33 | -2.33 | 7.14 | TNFSF4;LEF1 | |  | | |
| membrane raft distribution (GO:0031580) | 0.05 | 1.33 | -2.21 | 6.77 | CD2;MAL | |  | | |
| membrane raft polarization (GO:0001766) | 0.05 | 1.33 | -2.20 | 6.77 | CD2;MAL | |  | | |
| positive regulation of lamellipodium morphogenesis (GO:2000394) | 0.05 | 1.33 | -2.18 | 6.68 | VIL1;CORO1C | |  | | |
| regulation of natural killer cell chemotaxis (GO:2000501) | 0.05 | 1.33 | -2.15 | 6.59 | CCL5;CCL3 | |  | | |
| microglial cell activation (GO:0001774) | 0.05 | 1.33 | -2.13 | 6.55 | CLU;SNCA | |  | | |
| cAMP catabolic process (GO:0006198) | 0.05 | 1.33 | -1.93 | 5.93 | PDE3B;PDE4B | |  | | |
| pri-miRNA transcription from RNA polymerase II promoter (GO:0061614) | 0.05 | 1.33 | -1.89 | 5.80 | FOSL1;KLF4 | |  | | |
| positive regulation of transmembrane transport (GO:0034764) | 0.05 | 1.33 | -1.87 | 5.75 | CA2;ANO6 | |  | | |
| regulation of epidermal growth factor receptor signaling pathway (GO:0042058) | 0.05 | 1.33 | -1.23 | 3.76 | APP;EGF;HIP1R;PLAUR;PTPRJ;CBL | |  | | |
| positive regulation of B cell proliferation (GO:0030890) | 0.05 | 1.32 | -2.07 | 6.30 | TFRC;GPR183;BCL2;CD38 | |  | | |
| regulation of leukocyte chemotaxis (GO:0002688) | 0.05 | 1.32 | -1.62 | 4.95 | PPBP;CXCL5;PF4V1;PF4 | |  | | |
| regulation of protein insertion into mitochondrial membrane involved in apoptotic signaling pathway (GO:1900739) | 0.05 | 1.32 | -1.60 | 4.88 | PPP3CC;BCL2;PMAIP1;YWHAH | |  | | |
| positive regulation of protein insertion into mitochondrial membrane involved in apoptotic signaling pathway (GO:1900740) | 0.05 | 1.32 | -1.16 | 3.54 | PPP3CC;BCL2;PMAIP1;YWHAH | |  | | |
| response to estradiol (GO:0032355) | 0.05 | 1.32 | -1.09 | 3.32 | KAT5;NRIP1;ZNF703;CTNNB1 | |  | | |
| positive regulation of phosphorylation (GO:0042327) | 0.05 | 1.32 | -1.07 | 3.25 | APP;PFKFB3;EGF;ITGB3;PLAUR;AXIN2;RASGRP1;THBS1;HIF1A;PTK2;VEGFA;DAB2;CCL5;CDK2AP1;ITGA6 | |  | | |
| regulation of primary metabolic process (GO:0080090) | 0.05 | 1.31 | -0.85 | 2.57 | NCOA1;PSMC6;PSMD7;PSMD14;ID2;ODC1;G0S2;PLIN2;NR1D1;LDLR;PSMA7 | |  | | |
| lymphocyte chemotaxis (GO:0048247) | 0.05 | 1.31 | -1.10 | 3.33 | CCL20;GPR183;CCL5;CCL3;CXCL16 | |  | | |
| regulation of G-protein coupled receptor protein signaling pathway (GO:0008277) | 0.05 | 1.31 | -0.98 | 2.95 | FNTA;GRK5;GNG7;CCL5;PLEK;MGLL;RGS6 | |  | | |
| response to endoplasmic reticulum stress (GO:0034976) | 0.05 | 1.31 | -0.96 | 2.89 | JUN;HSPA5;BCL2;TXNDC11;HYOU1;THBS1;ATF3;NCK1;HSP90B1 | |  | | |
| peptide biosynthetic process (GO:0043043) | 0.05 | 1.30 | -1.16 | 3.48 | RPL30;RPL32;RPL36A;FURIN;RPL7;RPS25;RPS27;RPS15A;RPL27A;RPS20;RPS21;RPS13;EIF4G1 | |  | | |
| cell-matrix adhesion (GO:0007160) | 0.05 | 1.30 | -1.06 | 3.17 | ITGB1;CD96;CTTN;ITGB5;ITGB3;ACTN1;ADAM9;VCL | |  | | |
| positive regulation of response to external stimulus (GO:0032103) | 0.05 | 1.30 | -1.01 | 3.02 | THBD;DHX9;TNFSF4;CCL3;THBS1;BMP6;SNCA;MAPK13 | |  | | |
